# Positive Selection in Aggression-Linked Genes and Their Protein Interaction Networks

**DOI:** 10.3390/life16010015

**Published:** 2025-12-22

**Authors:** Asma Awadi, Zelalem Gebremariam Tolesa, Hichem Ben Slimen

**Affiliations:** 1 Laboratory of Functional Physiology and Valorization of Bioresources, Higher Institute of Biotechnology of Béja, University of Jendouba, Béja 9000, Tunisia; awadiasma@gmail.com; 2 Department of Microbial Cellular Molecular Biology, Addis Ababa University, Addis Ababa 1176, Ethiopia; basliel2018@gmail.com

**Keywords:** aggressive behavior, human, SNP, positive selection, adaptation

## Abstract

Aggressive behavior is a complex and multifactorial trait influenced by several genes and shaped by societal and cultural constraints. To trace adaptation signals and identify potential new genes related to aggressive behavior, we explored variations in nine genes previously linked to aggressive behavior, as well as their 74 interacting genes retrieved from the STRING database. We identified 15 SNPs under positive selection in four genes (*SEC24B*, *NCOA2*, *CTNNA1*, and *ALDH3A2*), with selection consistently confirmed by both iHS and xp-EHH analyses. Among these, 15 SNPs showed high pairwise F_ST_ values and pronounced allele frequency differences between populations, suggesting their potential role in the local adaptation of the studied populations. The functional importance of these SNPs was confirmed by ten acting as eQTLs and five located in transcription factor binding sequences. The observed selection signatures may reflect adaptation in diverse biological processes, including protein trafficking and signal transduction, cell proliferation and differentiation, endocrine regulation, and lipid and aldehyde detoxification. Although these processes are not directly linked to aggression, they may have downstream effects on neurodevelopmental and hormonal regulation that could indirectly influence behavioral phenotypes. Experimental validation is required to confirm these signals and to clarify their functional and biological significance.

## 1. Introduction

Over the past few years, the increase in genomic data being generated has improved our understanding of the genetic architecture and evolution of complex traits across diverse human populations. Among these traits, aggressive behavior has received increased attention because of its harmful impact on society [1]. The causes of this phenomenon arising from anger or antipathy and resulting in hostile or violent behavior [2] are complex and multifactorial. Indeed, aggressive behavior is shaped by societal and cultural constraints such as the availability of firearms, exposure to violence and experiences of sexual abuse [3,4]. In addition, several studies in both healthy and clinical populations have identified multiple genes associated with aggression (e.g., [5,6,7]). This genetic contribution is further corroborated by studies reporting that pathological aggression, associated with antisocial behavior or violence, is highly heritable [2]. For instance, Tuvblad and Baker [8] reported that approximately 50% of the variation in aggression is due to genetic factors. Among the genes related to aggression, there has been a focus on the monoamine oxidase A (*MAOA*) gene. Genetic deficiencies within this gene in both animal and human models were first identified through the presence of mutations, deletions, single-nucleotide polymorphisms (SNPs), and variable numbers of tandem repeats (VNTRs) [9,10]. Low expression levels of this gene have been linked to impulsive aggression and potentially increased propensities for violent behavior [6,11]. Other genes related to aggressive behavior include *MAOB*, *SLC63A* (*DAT1*), *DRD2* [12], *CDH13* [6], *OXTR* [13], *COMT*, *SLC6A4* [7] and *NR3C1* [14]. However, conflicting results have also been reported as Zammit et al. [5] found no evidence that functional polymorphisms within the *MAOA*, *MAOB*, or *COMT* genes are risk factors for aggressive behavior.

The human evolution from a hunter-gatherer lifestyle to a modern lifestyle included substantial changes to an increased level of social tolerance and a remarkable ability for prosocial behavior, including helping and cooperating with others [15]. Throughout this evolutionary process, social control has played a crucial role in reducing aggression in hunter-gatherer populations [16]. This reduced tendency for reactive aggressiveness is thought to have arisen as an incidental genetic consequence [17]. Understanding whether these behavioral changes, shaped by both genetic and cultural factors, were also influenced by natural selection is therefore essential. Garland et al. [18] suggested that behavior may often be a fairly direct target of natural or sexual selection. However, the effect of natural selection on the evolution of complex traits remains controversial. Aggressive behavior, for instance, is considered as an evolutionarily conserved heritable trait that is essential for survival and fitness [19]. Therefore, it might be shaped by negative selection as suggested by Zietsch [20] for the genetic architecture of complex traits. However, Wrangham [17] viewed aggressive behavior as adaptive and shaped by natural selection, a view supported by findings from several studies showing that variation in personality traits is generally under strong balancing selection [21,22,23].

Several mechanisms may have shaped the evolution of aggressive behavior. Among these, hormones were suggested to possibly shape behavioral traits across different life stages [18] with gonadal steroids, especially testosterone, exerting a strong effect on aggressive behavior. Testosterone has also been suggested to promote aggressive behavior in males, while engagement in competitive or aggressive interactions can, in turn, increase testosterone secretion [24]. On the other hand, alcohol and drug use have been consistently linked with aggression [4]. Dopamine levels can increase in the brain as a consequence of alcohol or amphetamine consumption which might result in aggressive behavior [25]. In addition, alcohol consumption can lead to changes in DNA methylation, which may alter gene expression and thereby influence the likelihood of developing alcohol use disorder [26,27].

In this study, we employed the integrated haplotype score (iHS) and the cross-population extended haplotype homozygosity (xp-EHH) to detect recent positive selection in nine genes previously linked to aggressive behavior as well as their 74 interacting genes retrieved from the STRING database across human populations with different ancestries. Candidate SNPs for positive selection showing significant genetic differentiation (F_ST_ values) and significant differences in allele frequency between populations are considered advantageous for a specific group. In addition, we incorporated functional genetic data to assess whether these variants could play a significant role in the adaptation to aggressive behavior or other complex traits in human populations. We believe that combining population genetic and functional approaches can increase our understanding of the genetics of aggressive behavior.

## 2. Materials and Methods

### 2.1. Investigated Genomic Regions

We selected nine genes that were suggested by the literature to be directly involved in aggressive behavior: *MAOA*, *MAOB*, *SLC63A*, *DRD2*, *CDH13*, *COMT*, *OXTR*, *SLC6A4* and *NR3C1* [6,7,12,13,14]. We also included their 74 interacting genes (Supplementary Table S1) as suggested by the exploration of protein–protein interactions reported in the String database (http://string-db.org/ (accessed on 12 June 2023)). Exploration of these additional genes offers an interesting starting point to identify new genes potentially involved in aggressive behavior.

### 2.2. Genomic Data

We retrieved single nucleotide polymorphism (SNP) data from 1198 individuals belonging to 12 human groups from the genome-wide genotype data from the 1KGP genomes project phase 3 (http://ftp.1000genomes.ebi.ac.uk/vol1/ftp/release/20130502/ (accessed on 10 June 2023)) [28]. Analyses were based on combining these groups according to their four genetic ancestries: African ancestry (AFR), European ancestry (EUR), South-Asian ancestry (SAS) and East-Asian ancestry (EAS) (Table 1). We used the PLINK 1.9 [29] and VCFtools v0.1.14 software [30] to process the variant call format (VCF) files. We also eliminated any variants that showed deviation from Hardy–Weinberg expectation with a significance level of *p*-value < 1 × 10^−6^.

For comparison purposes, we downloaded data on early Europeans [31] from the Reich lab webpage as well as sequence data from the Neanderthal and Denisovan genomes at http://cdna.eva.mpg.de/neandertal/altai/ (accessed on 20 June 2023) and https://bioinf.eva.mpg.de/download/HighCoverageDenisovaGenome/DenHC_catalog/ (accessed on 20 June 2023).

### 2.3. Population Genetics Analysis

Haplotype-based statistics such as iHS are affected by population demographic differences in contrary to cross-population methods like xp-EHH. Therefore, positive selection was detected within each population using iHS score [32] and between populations using xp-EHH. Both tests were calculated for each SNP with the software program Selscan version 1.2.0a [33]. The iHS is a statistic that compares the extended haplotype homozygosity (EHH) values between derived and ancestral alleles within a population. The xp-EHH method evaluates differences in haplotype homozygosity decay between two populations by comparing EHH at specific loci. A positive xp-EHH score indicates that haplotypes in the test population remain homozygous over a greater distance, suggesting stronger recent positive selection compared to the reference population. In contrast, a negative score points to stronger selection in the reference population. Our selection analyses were performed on phased chromosome data using the default parameters as implemented in the software Selscan. The obtained iHS and xp-EHH scores were then standardized over the whole genome using the script ‘*norm*’, provided with the Selscan software. Empirical *p*-values for the both scores were calculated across all chromosomes using the R program version 4.1.0 [34]. Accordingly, we considered iHS scores > 2.75 or <−2.75 and xp-EHH scores > 2.806 or <−2.806 as statistically significant (*p* < 0.01). For, iHS, potential chromosome regions under positive selection were identified as genomic regions encompassing clusters of ≥20 SNPs with |iHS| > 2.75 in nonoverlapping windows of 100 kb. SNPs were classified as candidates for positive selection when they exhibited statistically significant signals in both iHS and xp-EHH analyses, ensuring concordant evidence across complementary haplotype-based tests.

In order to evaluate potential confounding population substructure within each group, we used fastmixture [35] to estimate ancestry proportions and ancestral allele frequencies. Analysis was then performed across K = 2 to 20 on all populations and in the merged chromosomes. The highest likelihood score determined the optimal K which was used for final visualization. Population stratification was assessed using Principal Component Analysis (PCA) implemented in PLINK 1.9 [29]. Because recombination can influence haplotype structure and mimic signals of selection, we examined linkage disequilibrium (LD) decay patterns to account for recombination effects when interpreting selection signatures. All chromosomes were concatenated prior to LD estimation. LD was calculated using PLINK, with markers thinned using the — thin 0.01 option, and pairwise LD (r^2^) computed within a maximum physical distance of 200 kb. A custom Python script was then used to summarize LD decay by averaging r^2^ values in non-overlapping 5 kb distance bins, representing the mean LD for each 5 kb interval.

Patterns of differentiation among the four populations were investigated by calculating pairwise F_ST_ [36] between the different populations in each of the SNPs under positive selection with the PLINK program. Negative F_ST_ values were set to zero. Empirical *p*-values for pairwise F_ST_ were calculated for the entire genome to determine the significance level (*p* < 0.05) for outlier loci.

We calculated pairwise linkage disequilibrium (LD) between SNPs potentially under positive selection and the different SNPs of our query genes: MAOA, MAOB, SLC63A, DRD2, CDH13, and COMT, which are considered to have an effect on aggressive behavior. High LD (r^2^ > 0.95) is considered an indication of a plausible association between any two genes.

### 2.4. Functional Data

SNPs may be correlated with the expression of nearby genes, and hence can be considered as cis-eQTLs. To explore if any of the SNPs identified as being under positive selection also function as eQTLs, we utilized the expression quantitative trait loci data from the GTEx Portal V8 Release (https://www.gtexportal.org/home/ (accessed on 12 September 2023)) [37]. Our hypothesis was that SNPs influencing gene expression are more likely to be involved in adaptation affecting behavioral or other complex traits, compared with SNPs without such effects. In addition, we consulted the RegulomeDB database (https://regulomedb.org/ (accessed on 13 September 2023)) [38] to collect data on chromatin states, which encompass various known classes of genomic elements [39]. FunMotifs DB [40] was used to investigate the presence of transcription factor (TF) motifs within SNPs under selection. Analysis was based on genomic region including the concerned SNP with 10 positions up and downstream. Furthermore, we used the Reactome Database (https://reactome.org/ (accessed on 14 September 2023); [41]) to identify the different biological pathways in which the genes currently detected under positive selection are involved. Finally, trait data associated with our SNPs under positive selection were obtained from NHGRIEBI GWAS Catalog (https://www.ebi.ac.uk/gwas/ (accessed on 15 September 2023)) [42].

## 3. Results

### 3.1. Positive Selection and Genetic Differentiation

**Figure 1:**
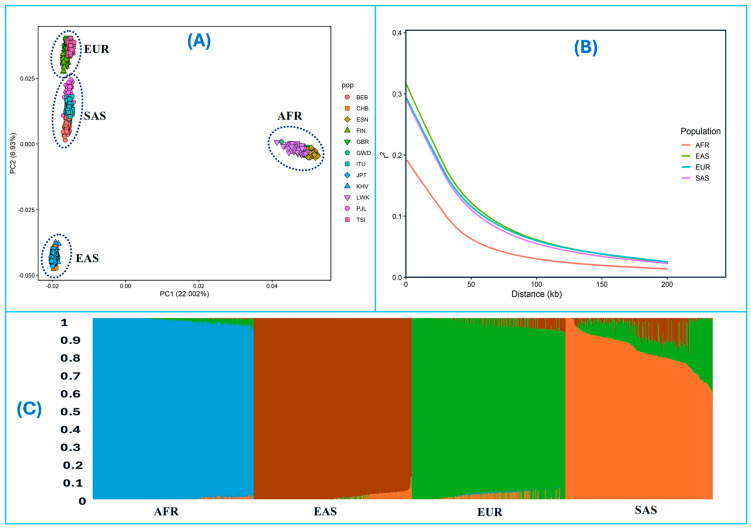
Population genetics analysis of African (AFR), East Asian (EAS), European (EUR), and South Asian (SAS) populations. (**A**) Principal component analysis (PCA) of the first two components showing the clustering of individuals from different populations. Each point represents an individual, colored according to population. (**B**) Linkage disequilibrium (LD) decay curves for the four populations. The average $r^{2}$ values are plotted against the physical distance between SNPs. (**C**) fast structure results assuming four genetic clusters (K = 4; model likelihood = −7,054,226,911.3). Each vertical bar represents an individual, and colors indicate the proportion of ancestry assigned to each cluster.

Principal component analysis (PCA) and fastmixture analyses support the genetic differentiation among the AFR, EUR, EAS, and SAS groups, with individuals clustering according to their superpopulation assignments. While admixture was generally limited, some individuals from the SAS population showed higher levels of admixture (Figure 1A,B). In addition, LD decay curves (Figure 1C) computed separately for the four groups reveal the expected differences in haplotype structure across the 1000 Genomes superpopulations [28], with African populations showing the most rapid LD decay and European and East Asian populations exhibiting slower decay. These results are consistent with known demographic histories and recombination patterns [28]. Together, these results indicate that the extended haplotype signals identified in this study are unlikely to be driven by abnormal LD structure or recombination rate anomalies. Therefore, haplotype-based selection statistics, including iHS and xp-EHH, can be reliably interpreted within and between these populations.

**Figure 2:**
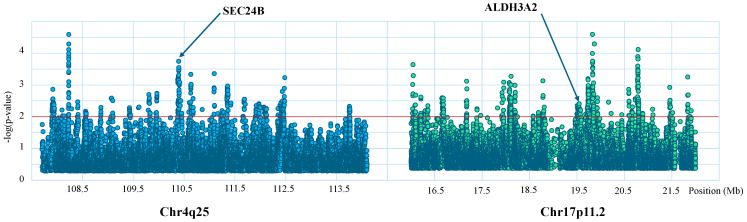
iHS values (*y*-axis) plotted across human chromosomal regions 4.q25 and 17.p11.2 for the African population (*x*-axis). Positions for the chromosomal regions are indicated in Mb, red lines indicate threshold for significant iHS scores (iHS > −2.75 or iHS > 2.75) and the positions of *SEC24B* and *ALDH3A2* genes are marked with arrows.

Using the integrated haplotype score (iHS) we identified signatures of positive selection in five genes: *ADH1B*, *SEC24B*, *CTNNA1*, *NCOA2* and *ALDH3A2* (Supplementary Table S2). While all the loci were under positive selection in Africans, *SEC24B* and *NCOA2* were also under positive selection in Europeans and the locus *CTNNA1* was also under positive selection in South Asian populations. The number of SNPs identified under positive selection per locus ranged from 1 (*ADH1B*) to 62 (*NCOA2*) with a total of 158 SNPs. SNPs under positive selection and their frequencies in the various populations are shown in Supplementary Table S2. iHS scores for the human chromosomal regions 4.q25 and 17.p11.2 in the African population are shown in Figure 2.

Signals of population-specific selection were further investigated using the xp-EHH test. The number of SNPs identified as being under positive selection per locus ranged from 5 (*NCOA2*) to 20 (*CTNNA1*) with a total of 42 SNPs (Supplementary Table S2) also showing consistent signals of positive selection in the iHS analysis. Significant xp-EHH signals were observed in Europeans at *SEC24B*, in South Asian populations at *CTNNA1*, and in East Asian population in *NCOA2*, using Africans as a reference population. In addition, evidence of positive selection was observed in East Asians at *ALDH3A2* when using Europeans as the test population. Among these, 15 SNPs exhibited significant pairwise FST values and were retained as candidates for adaptation (Table 2). This filtering ensures that our subsequent analyses focus on the most robust signals of selection.

Pairwise F_ST_ values for candidate SNPs under positive selection, calculated across all four populations, ranged from 0.00 to 0.730 (overall including all SNPs). Population pairwise F_ST_-values for all SNPs under positive selection are given in Supplementary Table S2. Patterns of differentiation among the 15 SNPs retained as candidates for positive selection varied across genes. For example, the positively selected SNPs in *ALDH3A2* differentiated EAS from AFR and EUR. In contrast, SNPs in *SEC24B*, *CTNNA1* and *NCOA2* genes differentiated the AFR population from all other populations, AFR from EAS, and AFR from EUR populations, respectively.

**Figure 3:**
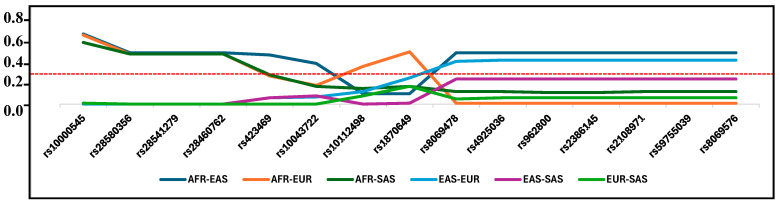
Variation in pairwise FST values for the 15 positively selected SNPs with significant differentiation. Dashed red line indicated the significance level for outlier FST > 0.3.

**Figure 4:**
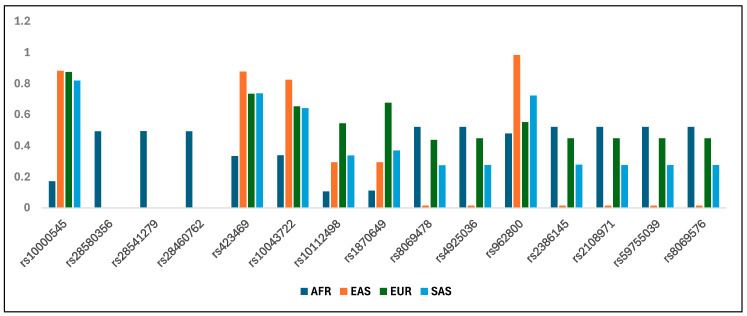
Allele frequency bar chart of the 15 SNPs under positive selection in the different populations.

SNPs with high and significant F_ST_ values between specific population pairs also clearly differed in allele frequency across the studied populations (Table 3, Figure 3). The most significant difference in allele frequency was observed for the *SEC24B* gene in all SNPs under positive selection in the population with African ancestry. The frequency of the derived beneficial alleles of these SNPs reaches almost 50% in African populations. In contrast, these alleles are very rare (0.2%) in European ancestry and are absent in East and South Asian ancestries. All beneficial alleles are new as they are absent or very rare in ancient Eurasians (Supplementary Table S2) and are absent in the Neanderthal and Denisovan genomes. Conversely, the beneficial alleles of SNP rs10000545 in *SEC24B* have high frequencies in EAS, EUR and SAS (>80%), whereas the AFR population show low frequencies of this allele. In the *ALDH3A2* gene, the frequency of beneficial alleles reaches almost 50% in populations with African and European ancestries. In the Asian populations, these alleles are very rare in the EAS population (1.6%) and are more common (28%) in the SAS population. Finally, SNPs of the *CTNNA1* and *NCOA2* genes which are under selection in the African population, had frequencies of 0.33 and 0.1, respectively (Table 3, Figure 4).

### 3.2. Functional Analyses Results

**Figure 5:**
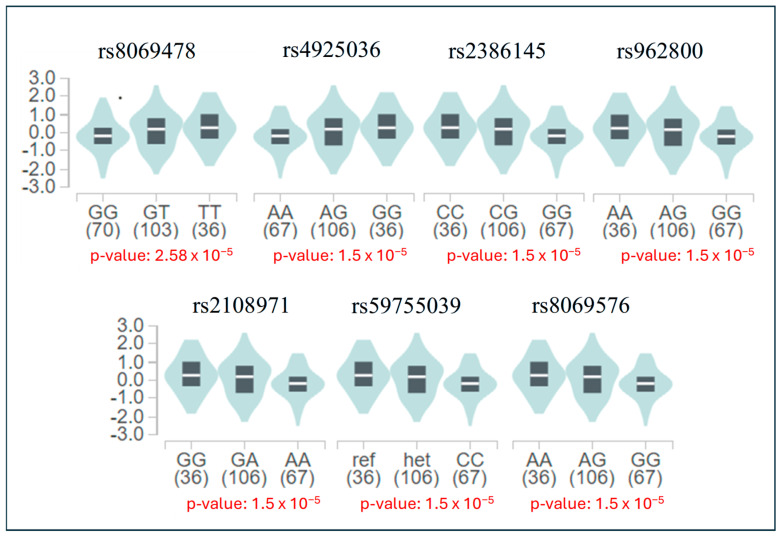
The significant eQTLs for the *ALDH3A2* variants shown in violin plots. All beneficial alleles were associated with increased expression of *ALDH3A2* gene in the brain except for rs4925036.

From the GTEx database, we identified a total of 65 positively selected SNPs that function as cis-eQTLs in different human tissue types for a total of 23 genes (Supplementary Table S3). Among our selected SNPs (Table 2), 10 were identified as eQTLs for a total of 9 genes (*SEC24B*, *SEC24B-AS1*, *RBMXP4*, *SETP20*, *AC034243.1*, *CTNNA1*, *SIL1*, *RP11-311F12.1*, and *ALDH3A2*). Beneficial alleles on these SNPs are associated with significant changes in gene expression, either increased or decreased, in various human tissues (Supplementary Table S3). Specifically, these alleles are associated with increased expression of *CTNNA1*, *RP11-311F12.1* and *ALDH3A2* in specific brain tissues or with decreased expression of *SIL1* and *SEC24B*. SNPs associated with significant variation in ALDH3A2 gene in the brain are indicated in Figure 5.

We further analyzed the chromatin state using data from the RegulomeDB webserver. The results obtained indicated that SNPs under positive selection are associated with different chromatin state such as Enhancers, Weak transcription, Weak enhancer, Active TSS… (Table 4). Furthermore, eight SNPs were located within binding regions for various transcription factors (TFs). These TFs are involved in a wide range of important biological pathways, as suggested from the reactome database, including gene expression (*BCL6*, *GATA1*), developmental biology (*ISL1*, *DUXA*), immune system (*BCL6*, *STAT4*, *STAT2*, *STAT5A*, *STAT5B*, *IRF1*, *BATF*, *BATF3*), signal transduction (*FOSL1*), disease (*STAT2*), protein metabolism (*ISL1*), and hemostasis (*GATA1*).

Our linkage disequilibrium results indicated that rs79293802 under positive selection in NCOA2 is in complete LD (D’ = 1) with the MAOB variant rs767099578. In addition, 14 out of the 31 selected SNPs in the *CTNNA1* gene are in LD with the *MAOB* variant rs2147154680 (Supplementary Table S2). Finally, among the 15 selected SNPs, only the variant rs10043722 in *CTNNA1* gene was associated with risk-taking behavior and insomnia as indicated by the GWAS Catalog.

## 4. Discussion

In humans, complex traits such as aggressive behavior are determined by the complex interplay between genetic variants and environmental factors. Experimental analyses of aggressive and antisocial behavior in human populations and different animal species have related them to various brain regions, neural networks, neurotransmitters, hormones, and specific genes [43]. These genes might have been shaped during human evolution by natural selection to promote regional behavioral adaptation under particular lifestyle and environmental conditions.

In the present study, we identified 15 SNPs under positive selection in four genes: *SEC24B*, *CTNNA1*, *NCOA2*, and A*LDH3A2*. All these genes encode proteins that perform essential functions in human tissues. *SEC24B* encodes a protein that belongs to the SEC24 subfamily within the SEC23/SEC24 family, which plays a crucial role in vesicular transport. Mutations in the *SEC24B* gene, which encode a component of the COPII vesicle, have been linked to Neural Tube Defects in humans [44]. The COPII-mediated vesicle transport has a key role in enabling the proper cellular localization of thousands of proteins [45], influencing diverse cellular processes. Therefore, signatures of positive selection in *SEC24B* may reflect adaptive changes affecting COPII-mediated trafficking, with potential downstream effects on metabolic or developmental processes. *CTNNA1* encodes α-catenin, which is part of the E-cadherin-catenin complex, involved in various cellular processes, including signal transduction, cell proliferation, differentiation, immune responses, and inflammation [46]. Positive selection in *CTNNA1* may reflect adaptive pressures on epithelial integrity, cell–cell adhesion, or signaling functions critical for tissue organization. *NCOA2*, known as nuclear receptor coactivator 2, is significant in numerous physiological processes, including endocrine regulation, metabolic regulation, and tumorigenesis [47]. Signatures of positive selection in *NCOA2* may therefore reflect evolutionary pressures acting on nuclear hormone receptor–mediated pathways. Given the central role of steroid hormones in growth, energy balance, and fertility, adaptive variation in *NCOA2* could have influenced metabolic and hormonal regulation in response to changing environmental conditions. Finally, *ALDH3A2* plays an important role in detoxifying long-chain aliphatic aldehydes produced during lipid peroxidation and oxidative stress [48]. Over 70 mutations in the *ALDH3A2* gene have been identified in patients diagnosed with Sjögren–Larsson syndrome (SLS), who exhibit a wide range of neurological manifestations alongside the skin symptom of ichthyosis [49]. Signatures of positive selection in *ALDH3A2* may therefore reflect adaptive pressures related to oxidative stress management and cellular detoxification, potentially enhancing resilience to environmental stressors.

Although the SNPs identified under positive selection are not directly associated with aggression, related variants in *SEC24B*, *CTNNA1*, and *NCOA2* have been linked to traits such as testosterone levels, risk-taking behavior, and sex hormone-binding globulin concentrations, which are known to influence aggression in a complex and context-dependent manner. Indeed, data from GWAS catalog reported that rs535305978-C and rs187325356 *SEC24B* are associated with total testosterone levels, rs114916656-T and rs10043722-A *CTNNA1* being associated with risk taking behavior. Finally, *NCOA2* rs75349541-C, rs72663955-T were suggested to be associated with sex hormone-binding globulin levels. These associations, reported in the GWAS Catalog, suggest that genetic variation in these loci may influence behavioral phenotypes indirectly through hormonal and behavioral pathways.

Among SNPs under positive selection, only rs10000545 in *SEC24B* was reported to be under selection in EUR population; all the others were under selection in the African population. Despite being located in non-coding regions, the importance of these selected SNPs is highlighted by their function as eQTLs (ten SNPs) for several genes as revealed by the GTEx database. The derived beneficial alleles are associated with significant variation (increase or decrease) in the expression of eGene in different human tissues. Specifically, several SNPs variants (Table 2) are associated with expression differences in *SEC24B*, *SIL1*, *RP11-311F12.1* and *ALDH3A2* in brain tissues, suggesting possible regulatory effects in neural contexts. Indeed, the brain has been identified as a crucial region associated with aggressive behavior (e.g., [6,50]), supporting the relevance of investigating regulatory variation affecting gene expression in this tissue. The seven positively selected SNPs, that function as eQTLs for the *ALDH3A2* gene, exhibit significant differential expression across multiple tissues, including brain, lung, testis, and thyroid, with alleles inferred to be under positive selection generally associated with increased expression, except for rs962800. Within the brain, significant expression differences were observed specifically in the cerebellum, a region increasingly recognized for its involvement in anger and aggressive behavior in human [51]. Increased expression of *ALDH3A2* in brain tissues may therefore enhance the clearance of neurotoxic aldehydes arising from oxidative stress or monoamine metabolism, including pathways involving dopamine, norepinephrine, and serotonin [52]. While these findings suggest a potential role for *ALDH3A2* expression in neural processes, the functional implications remain unclear. One possible explanation is that increased *ALDH3A2* expression could enhance detoxification of reactive aldehydes generated through endogenous metabolic processes, including lipid peroxidation and the metabolism of monoamine neurotransmitters such as dopamine, norepinephrine, and serotonin [6,52]. This interpretation is consistent with GTEx data showing no significant genotype-dependent differences in *ALDH3A2* expression in the liver or whole blood, indicating that tissue-specific regulation in the brain may be particularly relevant. However, further functional studies are needed to confirm whether these expression patterns represent an adaptive response.

Aggressive behavior is currently viewed as adaptive or derived from adaptive strategies [17] and therefore was shaped by natural selection during human evolution. However, the direction of evolution towards lower or higher aggressive behavior is still debated although Wrangham [17] (see also [15]) suggested that humans have evolved towards lower aggressive behavior as evidenced from genetic comparisons of *H. sapiens*, Neandertals and Denisovans. Similarly, our comparison of alleles in aggression-related-genes revealed differences between modern human populations and ancestral Neandertals and Denisovan variants. In addition, almost all alleles under positive selection represent derived variants compared to the ancestral Neandertals and Denisovan alleles which support their potential role during human adaptation to a modern lifestyle. Overall, the presence of these derived alleles under positive selection highlights ongoing adaptation in modern human populations, affecting a variety of cellular and physiological processes not necessarily linked to aggressive behavior.

## 5. Conclusions

Among all studied genes linked to aggressive behavior and their interacting genes, only 15 SNPs in four genes (*CTNNA1*, *NCOA2*, *ALDH3A2*, and *SEC24B*) were consistently identified under positive selection by both methods and also exhibited significant pairwise FST values. While these genes are not canonical aggression-related genes, functional evidence from eQTLs and GWAS data suggests they may influence biological pathways that could indirectly affect aggression-related traits. Further analyses, including experimental validation, in both pathological and healthy human populations, are essential to confirm the identified genetic signals and substantiate their biological and clinical relevance.

## Figures and Tables

**Table 1:** The 1KPG populations included in the current study and their genetic ancestries (http://ftp.1000genomes.ebi.ac.uk/vol1/ftp/release/20130502/ (accessed on 10 June 2023)).

Population (Code)	Regional Population Description (Code)	Number of Individuals
Africa (AFR)	Luhya in Webuye, Kenya (LWK)	99
	Gambians from The Gambia (GWD)	113
	Esan in Nigeria (ESN)	99
Europe (EUR)	British in England and Scotland (GBR)	91
	Finnish in Finland (FIN)	99
	Toscani in Italia (TSI)	107
South Asian (SAS)	Bengali from Bangladesh (BEB)	86
	Indian Telugu from the UK (ITU)	102
	Punjabi from Lahore, Pakistan (PJL)	96
East Asian (EAS)	Han Chinese in Beijing, China (CHB)	103
	Japanese in Tokyo, Japan (JPT)	104
	Kinhin Ho Chi Minh City, Vietnam (KHV)	99

**Table 2:** SNPs under positive selection in human populations with different ancestries (AFR: Africa, EUR: Europe, SAS: South Asia, EAS: East Asia) and their iHS and xp-EHH scores. Observed alleles for the Neanderthal (N) and Denisovan (D) genomes.

Beneficial Allele/Alternate Allele	Location	iHS		Xp-EHH		
		Score	Pop.	Score	Pop.	N/D
rs10000545-G/C	Intron, SEC24B	−2.96	EUR	3.59	EAS-AFR EUR-AFR	C
rs28580356-T/C	Intron, SEC24B	3.06	AFR	3.87		C
rs28541279-G/T	Intron, SEC24B	3.04	AFR	3.85		T
rs28460762-C/A	Intron, SEC24B	4.07	AFR	3.85		A
rs423469-G/C	Intron, CTNNA1	−3.22	AFR	3.30	EAS-AFR	C
rs10043722-A/G	Intron, CTNNA1	−3.16	AFR	3.26		G
rs10112498-G/T	Intron, NCOA2	4.32	AFR	2.93	EUR-AFR	T
rs1870649-C/G	Intron, NCOA2	−3.50	AFR	3.21		G
rs8069478-T/G	Intron, ALDH3A2	3.00	AFR	−3.23	EUR-EAS	G
rs4925036-G/A	Intron, ALDH3A2	3.00	AFR	−3.21		A
rs962800-G/A	Intron, ALDH3A2	−3.07	AFR	−2.99		G
rs2386145-C/G	Intron, ALDH3A2	−2.93	AFR	−3.06		G
rs2108971-G/A	Intron, ALDH3A2	−2.81	AFR	−3.20		A
rs59755039-CT/C	Intron, ALDH3A2	−2.85	AFR	−3.41		C
rs8069576-A/G	Intron, ALDH3A2	−3.07	AFR	−3.11		G

**Table 3:** Pairwise F_ST_ values, allele frequency in % for the derived beneficial allele/alternate allele in the different ancestries (AFR: Africa, EUR: Europe, SAS: South Asia, EAS: East Asia) and observed alleles for the Neanderthal (N) and Denisovan (D) genomes.

Beneficial Allele/Alternate Allele	Pairwise FST						Frequency Beneficial			
	AFR-EAS	AFR-EUR	AFR-SAS	EAS-EUR	EAS-SAS	EUR-SAS	AFR	EAS	EUR	SAS
rs10000545-G/C	0.67	0.66	0.59	0.00	0.01	0.01	0.172	0.882	0.874	0.819
rs28580356-T/C	0.49	0.48	0.48	0.00	-	0.00	0.492	0.000	0.002	0.000
rs28541279-G/T	0.49	0.48	0.48	0.00	-	0.00	0.494	0.000	0.002	0.000
rs28460762-C/A	0.49	0.48	0.48	0.00	-	0.00	0.492	0.000	0.002	0.000
rs423469-G/C	0.47	0.27	0.28	0.06	0.06	0.00	0.334	0.877	0.734	0.738
rs10043722-A/G	0.39	0.18	0.17	0.07	0.08	0.00	0.339	0.824	0.653	0.641
rs10112498-G/T	0.10	0.36	0.15	0.12	0.00	0.08	0.106	0.294	0.544	0.338
rs1870649-C/G	0.10	0.50	0.17	0.25	0.01	0.17	0.111	0.294	0.677	0.37
rs8069478-T/G	0.49	0.01	0.12	0.41	0.24	0.05	0.521	0.016	0.438	0.275
rs4925036-G/A	0.49	0.01	0.12	0.42	0.24	0.06	0.521	0.016	0.449	0.276
rs962800-G/A	0.49	0.01	0.11	0.42	0.24	0.06	0.479	0.984	0.551	0.722
rs2386145-C/G	0.49	0.01	0.11	0.42	0.24	0.06	0.521	0.016	0.449	0.278
rs2108971-G/A	0.49	0.01	0.12	0.42	0.24	0.06	0.521	0.016	0.449	0.276
rs59755039-CT/C	0.49	0.01	0.12	0.42	0.24	0.06	0.521	0.016	0.449	0.276
rs8069576-A/G	0.49	0.01	0.12	0.42	0.24	0.06	0.521	0.016	0.449	0.276

**Table 4:** GTEx and RegulomeDB data on SNPs under positive selection. GTEx eQTLs–eGene interaction with *p* < 0.0001. The RegulomeDB probability score ranges from 0 to 1, with 1 being most likely to be a regulatory variant (for details see [38]). RegulomeDB rank: 1f: eQTL/caQTL + TF binding/chromatin accessibility peak; 1b: eQTL/caQTL + TF binding + any motif + Footprint + chromatin accessibility peak; 5: TF binding + DNase peak; 6: motif hit; 7: other. TF motifs were predicted from the funMotifs database for blood tissue.

eQTL	eGene	RegulomeDB		Chromatin State	Motifs
		Score	Rank		
rs10000545	*SEC24B*, *SEC24B-AS1*, *RBMXP4*, *SETP20*	0.66703	1f	Active TSS, Flanking active TSS, Weak enhancer, Weak transcription, Quiescent/Low	*BCL6*, *ISL1*, *STAT4*, *STAT5A*, *STAT5B*
rs28580356	*-*	0.51392	7	Strong transcription, Weak transcription, Quiescent/Low,	*-*
rs28541279	*-*	0.51392	7	Strong transcription, Weak transcription, Quiescent/Low,	*-*
rs28460762	*-*	0.51392	7	Enhancers, Strong transcription, Weak transcription, Quiescent/Low	*-*
rs423469	*AC034243.1*, *CTNNA1*, *SIL1*	0.22271	1f	Strong transcription, Weak transcription, Quiescent/Low	*-*
rs10043722	*AC034243.1*, *CTNNA1*, *SIL1*	0.94667	1b	Genic enhancers, Enhancers, Strong transcription, Weak transcription, Quiescent/Low	*-*
rs10112498	*-*	0.58955	5	Enhancers, Weak transcription, Quiescent/Low	*-*
rs1870649	*-*	0.18412	7	Active TSS, Enhancers, Weak transcription, Quiescent/Low	*ZNF384*
rs8069478	*RP11-311F12.1*, *ALDH3A2*	0.55436	1f	Active TSS, Flanking active TSS, Enhancers, Weak transcription, Bivalent/Poised TSS, Weak repressed polycomb, Quiescent/Low	*-*
rs4925036	*RP11-311F12.1*, *ALDH3A2*	0.66703	1f	Active TSS, Flanking active TSS, Enhancers, Weak transcription, Bivalent/Poised TSS, Weak repressed polycomb, Quiescent/Low	*BATF*, *BATF3*, *DUXA*, *FOSL1*
rs962800	*RP11-311F12.1*, *ALDH3A2*	0.66703	1f	Genic enhancers, Transcr. at 5’ and 3’, Strong transcription, Weak transcription, Repressed polycomb, Weak repressed polycomb, Heterochromatin, Quiescent/Low	*GATA1*
rs2386145	*RP11-311F12.1*, *ALDH3A2*	0.55436	1f	Genic enhancers, Enhancers, Strong transcription, Weak transcription, Weak repressed polycomb, Quiescent/Low	*-*
rs2108971	*RP11-311F12.1*, *ALDH3A2*	0.51392	7	Genic enhancers, Strong transcription, Weak transcription, Weak repressed polycomb, Quiescent/Low	*-*
rs59755039	*RP11-311F12.1*, *ALDH3A2*	0.55436	1f	Genic enhancers, Strong transcription, Weak transcription, Weak repressed polycomb, Quiescent/Low	*IRF1*, *STAT2*
rs8069576	*RP11-311F12.1*, *ALDH3A2*	0.51392	7	Strong transcription, Weak transcription, Weak repressed polycomb, Quiescent/Low	

## Data Availability

The data that supports the findings of this study are available in the Supplementary Material of this article.

## Supplementary Materials

Additional supporting information can be found online in the Supporting Information section: https://www.mdpi.com/article/10.3390/life16010015/s1 (Supporting Information). Table S1: List of investigated genes during the current study. Table S2: List of all SNPs under positive selection for each gene. Table S3: Identified eGene for each SNPs, *p*-values and tissues where these eGenes are expressed.
