# Supplementary material for: Positive Selection in Aggression-Linked Genes and Their Protein Interaction Networks
Source: Life (Basel). 2025 Dec 22;16(1):15. doi: 10.3390/life16010015 (PMC12842650; doi:10.3390/life16010015)
Supplement: Supplementary file 1 [file life-16-00015-s001.zip › life-4012880-supplementary.pdf]

Positive Selection in Aggression-Linked Genes and Their Protein Interaction Networks

Asma Awadi[1], Zelalem G. Tolesa[2], Hichem Ben Slimen[1]

[1] Laboratory of Functional Physiology and Valorization of Bioresources, Higher Institute of Biotechnology of Béja, 9000 Béja, University of Jendouba, Tunisia.

[2] Department of Microbial Cellular Molecular Biology, Addis Ababa University, Ethiopia

Table S1: List of investigated genes during the current study

Gene	Position	Genomic region
**YWHAZ**	chr8:101,930,804-101,965,221	q22.3
**ASMT**	chrX:1,733,941-1,761,974	p22.33
**DDC**	chr7:50,526,134-50,628,768	p12.1
**MAOA**	chrX:43,514,155-43,606,071	p11.3
**MAOB**	chrX:43,625,857-43,741,721	p11.3
**IDO2**	chr8:39,792,474-39,873,910	p11.21
**INMT**	chr7:30,791,751-30,797,218	p14.3
**IDO1**	chr8:39,771,328-39,786,309	p11.21
**SLC38A2**	chr12:46,751,971-46,766,645	q13.11
**BHLHE40**	chr3:5,021,097-5,026,865	p26.1
**SNCA**	chr4:90,645,250-90,759,447	q22.1
**DRD2**	chr11:113,280,317-113,346,001	q23.2
**PICK1**	chr22:38,453,262-38,471,708	q13.1
**STX1A**	chr7:73,113,535-73,134,017	q11.23
**SLC18A2**	chr10:119,000,584-119,038,941	q25.3
**FLOT1**	chr6:30,695,511-30,710,453	p21.33
**SYNGR3**	chr16:2,039,946-2,044,276	p13.3
**TH**	chr11:2,185,159-2,193,035	p15.5
**FLOT2**	chr17:27,206,357-27,224,715	q11.2
**CDH1**	chr16:68,771,195-68,869,444	q22.1
**COMT**	chr22:19,929,263-19,957,498	q11.21
**AOC2**	chr17:40,996,609-41,002,724	p13.3
**DBH**	chr9:136,501,485-136,524,466	q34.2
**CYP2D6**	chr22:42,522,501-42,526,883	q13.2
**HNMT**	chr2:138,721,808-138,773,934	q22.1
**PNMT**	chr17:37,824,706-37,826,728	q12
**ALDH2**	chr12:112,204,691-112,247,789	q24.12
**ALDH3B2**	chr11:67,429,633-67,448,685	q13.2
**ALDH3A2**	chr17:19,552,064-19,580,908	p11.2
**NCS1**	chr9:132,934,857-132,999,583	q34.11
**CNR1**	chr6:88,849,585-88,875,767	q15
**ARRB2**	chr17:4,613,789-4,624,795	p13.2
**SLC6A3**	chr5:1,392,905-1,445,543	p15.33
**GRIN2B**	chr12:13,714,410-14,133,022	p13.1
**ADRBK1 (GRK2)**	chr11:67,033,905-67,054,029	q13.2
**SIGMAR1**	chr9:34,634,719-34,637,768	p13.3
**DISC1**	chr1:231,762,561-232,177,019	q42.2
**SAG**	chr2:234,216,309-234,255,701	q37.1
**OXTR**	chr3:8,792,095-8,811,300	p25.3
**ADIPOQ**	chr3:186,560,463-186,576,252	q27.3
**HSPA5**	chr9:127,997,127-128,003,666	q33.3
**ADIPOR2**	chr12:1,800,247-1,897,845	p13.33
**CTNNB1**	chr3:41,240,942-41,281,939	p22.1

**ADIPOR1**	chr1:202,909,960-202,927,524	q32.1
**CTNND1**	chr11:57,529,234-57,586,652	q12.1
**CTNNA1**	chr5:138,089,107-138,270,723	q31.2
**RASSF1**	chr3:50,367,217-50,378,367	p21.31
**APC2**	chr19:1,450,148-1,473,243	p13.3
**JUP**	chr17:39,910,859-39,942,964	q21.2
**CDH13**	chr16:82,660,399-83,830,215	q23.3
**CYP1B1**	chr2:38,294,746-38,303,323	p22.2
**CYP1A1**	chr15:75,011,883-75,017,877	q24.1
**ADH1B**	chr4:100,227,527-100,242,572	q23
**FKBP4**	chr12:2,904,108-2,914,587	p13.33
**HSP90AA1**	chr14:102,547,075-102,606,086	q32.31
**NCOA1**	chr2:24,807,345-24,993,570	p32.3
**NCOA2**	chr8:71,024,267-71,316,020	q13.3
**FKBP5**	chr6:35,541,362-35,656,719	p21.31
**JUN**	chr1:59,246,463-59,249,785	p32.1
**SMARCA4**	chr19:11,071,598-11,172,958	p13.2
**EP300**	chr22:41,488,614-41,576,081	q13.2
**CREBBP**	chr16:3,775,056-3,930,121	p13.3
**HSPA4**	chr5:132,387,662-132,440,709	q31.1
**OXT**	chr20:3,052,266-3,053,162	p13
**GNAQ**	chr9:80,335,191-80,646,219	q21.2
**AVP**	chr20:3,063,202-3,065,370	p13
**GNAS 4 et 2**	chr20:57,414,795-57,486,250	q13.32
**GNAI2**	chr3:50,273,647-50,296,786	p21.31
**GNAI3**	chr1:110,091,186-110,138,454	p13.3
**GNAI1**	chr7:79,764,140-79,848,725	q21.11
**NPY**	chr7:24,323,807-24,331,484	p15.3
**HTR1A**	chr5:63,255,875-63,258,119	q12.3
**PPP2R4 (PTPA)**	chr9:131,873,593-131,911,225	q34.11
**CANX**	chr5:179,125,930-179,158,639	q35.3
**TPH1**	chr11:18,042,084-18,062,335	p15.1
**SEC24C**	chr10:75,504,131-75,531,933	q22.2
**HTR2A**	chr13:47,405,677-47,471,211	q14.2
**TPH2**	chr12:72,332,626-72,426,221	q21.1
**BDNF**	chr11:27,676,442-27,722,600	p14.1
**SEC24B**	chr4:110,354,971-110,461,615	4q25
**SLC6A4**	chr17:28,521,337-28,562,986	q11.2
**NR3C1**	chr5:142,657,496-142,784,045	q31.3

Table S2: List of all SNPs under positive selection for each gene. Their positions, iHS values and the populations where they were detected under positive selection are provided. Pairwise FST and allele frequencies for the alternate (ALT) allele are also included. Ancestral alleles are given in hunter-gatherer populations (Mathieson et al., 2015) as well as Neandertal and Denisovan genomes. Functional data such as Gtex eGene, RegulomeDB scores, Chromatin state, TF motif and GWAS information are also included. LD results are also provided when possible. For more details see M&M section.

Allele	POS	REF	ALT	iHS	Population	Xp-EHH values	Pairwise FST						Frequency of the alternate allele (ALT)				Alleles in hunther-gatherer			Ancient alleles	
							AFR-EAS	AFR-EUR	AFR-SAS	EAS-EUR	EAS-SAS	EUR-SAS	AFR	EAS	EUR	SAS	n individuals	allele 1	allele 2	Neandertal	DENISOVAN
rs1579513969	100238002	C	CT	2.77898	EAS		0,642	0,118	0,390	0,383	0,107	0,129	0,084	0,763	0,276	0,535				C	C
rs72896632	110354973	C	T	-2.83993	EUR	3.77438	0,035	0,044	0,007	0	0,009	0,014	0,236	0,131	0,120	0,183				T	T
rs10000545	110357878	G	C	-2.96359	EUR	3.59182	0,671	0,660	0,589	0	0,014	0,010	0,828	0,118	0,126	0,181				C	C
rs11942858	110358103	A	G	-2.83961	EUR	3.79509	0,081	0,079	0,029	0	0,013	0,012	0,285	0,118	0,120	0,180				G	G
rs11933971	110358288	C	T	-2.83961	EUR	3.72029	0,083	0,080	0,029	0	0,013	0,012	0,286	0,118	0,120	0,180				T	T
rs34836476	110361998	T	C	-2.99972	EUR	1.22476	0,081	0,079	0,030	0	0,013	0,012	0,285	0,118	0,120	0,178				C	C
rs13103034	110362373	G	C	-2.99972	EUR	1.22671	0,081	0,079	0,029	0	0,013	0,012	0,285	0,118	0,120	0,180				C	C
rs6817954	110362752	T	A	2.90881	EUR	1.26079	0,730	0,719	0,654	0	0,014	0,010	0,124	0,882	0,874	0,819				T	T
.	110365370	CA	C	-3.40724	EUR	1.38271	0,011	0,001	0,007	0,026	0,000	0,019	0,133	0,083	0,160	0,092				C	C
rs17040436	110365426	A	G	-3.07735	EUR	1.45177	0,123	0,114	0,056	0	0,014	0,011	0,333	0,118	0,125	0,181				G	G
rs17040439	110365515	A	T	-2.98544	EUR	1.43289	0,081	0,079	0,029	0	0,013	0,012	0,285	0,118	0,120	0,180				T	T
.	110365628	ATT	A	-3.69745	EUR	1.58291	0,589	0,646	0,562	0,008	0,000	0,017	0,765	0,123	0,081	0,141				A	A
.	110373232	GA	G	-2.97514	EUR	1.08267	0,042	0,075	0,034	0,004	0,000	0,007	0,386	0,247	0,204	0,261				G	G
rs76264283	110373544	T	C	2.7538	AFR	0.276055	0,085	0,079	0,082	0,000	nan	0,000	0,087	0,000	0,002	0,000				T	T
rs10009463	110373682	A	G	4.57244	AFR	0.359298	0,487	0,480	0,478	0,000	nan	0,000	0,490	0,000	0,002	0,000	164	A:1	G:0	A	A
.	110375694	CA	C	4.46638	AFR	3.22781	0,386	0,406	0,353	0,000	0,001	0,007	0,477	0,046	0,034	0,062				C	C
rs28597954	110376162	C	T	4.50719	AFR	0.300086	0,487	0,480	0,472	0,000	0,002	-0,001	0,490	0,000	0,002	0,004				C	C
rs28663368	110376396	A	G	4.50719	AFR	0.297289	0,487	0,480	0,478	0,000	nan	0,000	0,490	0,000	0,002	0,000				A	A
rs112585058	110378131	G	A	3.29749	AFR	0.144541	0,080	0,079	0,077	nan	nan	nan	0,082	0,000	0,000	0,000				G	G
rs34132975	110378352	G	A	2.91389	AFR	0.121607	0,069	0,000	-0,001	0,084	0,062	0,002	0,071	0,000	0,084	0,062				G	G
rs77911134	110379266	A	G	2.98576	AFR	-0.252708	0,007	0,018	0,060	0,001	0,029	0,017	0,082	0,049	0,035	0,007				A	A
rs79567241	110379367	A	T	3.26434	AFR	-0.218631	0,085	0,084	0,082	nan	nan	nan	0,087	0,000	0,000	0,000				A	A
rs76784285	110379670	G	T	2.97934	AFR	-0.340876	0,007	0,018	0,060	0,001	0,029	0,017	0,082	0,049	0,035	0,007				G	G
.	110381786	CTG	C	3.25659	AFR	0.41927	0,085	0,084	0,082	nan	nan	nan	0,087	0,000	0,000	0,000				C	C
rs7697001	110381952	G	A	4.71835	AFR	0.428297	0,487	0,480	0,478	0,000	nan	0,000	0,490	0,000	0,002	0,000				G	G
rs78653746	110382720	T	G	3.26342	AFR	0.268654	0,085	0,084	0,082	nan	nan	nan	0,087	0,000	0,000	0,000				T	T
rs28680423	110385187	C	G	4.87268	AFR	0.48412	0,489	0,482	0,480	0,000	nan	0,000	0,492	0,000	0,002	0,000				C	C
rs6840561	110385601	A	G	4.87268	AFR	0.519852	0,489	0,482	0,480	0,000	nan	0,000	0,492	0,000	0,002	0,000				A	A
rs17040454	110385758	T	A	2.99827	AFR	0.448505	0,007	0,018	0,060	0,001	0,029	0,017	0,082	0,049	0,035	0,007				T	T
rs12643881	110386443	T	A	2.99416	AFR	0.426679	0,007	0,018	0,060	0,001	0,029	0,017	0,082	0,049	0,035	0,007				T	T
rs113756500	110387074	T	G	3.55806	AFR	0.45027	0,056	0,055	0,054	nan	nan	nan	0,058	0,000	0,000	0,000				T	T
rs28412073	110387569	G	C	4.91237	AFR	0.634658	0,473	0,466	0,464	0,000	nan	0,000	0,476	0,000	0,002	0,000				G	G
rs77573441	110387757	A	G	3.00205	AFR	0.534222	0,034	0,005	0,000	0,064	0,035	0,005	0,066	0,013	0,096	0,065				A	A
.	110388248	C	CGAG	3.0073	AFR	1.29372	0,007	0,018	0,060	0,001	0,029	0,017	0,082	0,049	0,035	0,007				C	C
rs184928998	110388254	G	A	3.28327	AFR	0.234967	0,001	0,084	0,002	0,066	0,009	0,112	0,087	0,069	0,000	0,111				A	G
rs10706780	110389283	AC	A	4.69846	AFR	0.72189	0,467	0,459	0,457	0,000	nan	0,000	0,469	0,000	0,002	0,000				A	A
rs34813537	110389580	C	A	2.92266	AFR	0.463103	0,069	0,000	-0,001	0,084	0,062	0,002	0,071	0,000	0,084	0,062				C	C
rs74761343	110390687	G	A	3.27106	AFR	0.49976	0,085	0,084	0,082	nan	nan	nan	0,087	0,000	0,000	0,000				G	G
rs112761722	110390833	A	G	3.55693	AFR	0.47152	0,056	0,055	0,054	nan	nan	nan	0,058	0,000	0,000	0,000				A	A
rs7681265	110391962	C	A	4.53559	AFR	0.97701	0,487	0,480	0,478	0,000	nan	0,000	0,490	0,000	0,002	0,000	220	C:0.990909	A:0.00909091	C	C
rs10606064	110392793	CAT	C	4.52313	AFR	1.18345	0,473	0,466	0,464	0,000	nan	0,000	0,476	0,000	0,002	0,000				C	C
rs182286635	110392795	T	C	5.94667	AFR	1.30546	0,077	0,137	0,139	0,024	0,028	0,000	0,146	0,031	0,002	0,000				T	T
rs17040466	110393650	A	G	5.08981	AFR	1.04678	0,467	0,459	0,457	0,000	nan	0,000	0,469	0,000	0,002	0,000				A	A
rs67518796	110393749	TACTC	T	5.33514	AFR	0.79172	0,069	0,000	0,000	0,084	0,062	0,002	0,071	0,000	0,084	0,062				T	T
rs10516556	110395146	T	C	5.50689	AFR	0.402465	0,201	0,181	0,078	-0,001	0,040	0,028	0,076	0,350	0,328	0,218	150	T:0.613333	C:0.386667	T	T

rs10516557	110395406	G	A	5.46904	AFR	1.23152	0,007	0,018	0,060	0,001	0,029	0,017	0,082	0,049	0,035	0,007				G	G
rs78262578	110396045	C	T	5.6565	AFR	1.27577	0,085	0,084	0,082	nan	nan	nan	0,087	0,000	0,000	0,000				C	C
.	110397146	CT	C	-3.1188	EUR	1.12153	0,548	0,578	0,514	0,000	0,001	0,007	0,793	0,178	0,155	0,204				C	C
.	110397721	A	G	3.96133	AFR	1.49334	0,062	0,001	-0,002	0,084	0,062	0,002	0,064	0,000	0,084	0,062				A	A
rs34320670	110397724	T	A	3.96133	AFR	1.49334	0,062	0,001	-0,002	0,084	0,062	0,002	0,064	0,000	0,084	0,062				T	T
rs28580356	110398287	C	T	3.05877	AFR	3.86950	0,489	0,482	0,480	0,000	nan	0,000	0,492	0,000	0,002	0,000				C	C
rs28541279	110398890	T	G	3.04427	AFR	3.84760	0,491	0,484	0,482	0,000	nan	0,000	0,494	0,000	0,002	0,000				T	T
rs35866656	110399231	A	G	3.97254	AFR	3.78803	0,062	0,001	-0,002	0,084	0,062	0,002	0,064	0,000	0,084	0,062				A	A
rs6851756	110399303	G	A	4.40988	AFR	3.81350	0,085	0,084	0,082	nan	nan	nan	0,087	0,000	0,000	0,000				G	G
.	110399441	TA	T	-2.97648	EUR	2.39443	0,026	0,015	0,000	0,000	0,016	0,007	0,241	0,147	0,167	0,220				T	T
rs28460762	110399613	A	C	4.06811	AFR	3.84864	0,489	0,482	0,480	0,000	nan	0,000	0,492	0,000	0,002	0,000				A	A
rs700626	138117938	A	G	-2.89197	SAS	2.69273	0,154	0,002	0,003	0,118	0,114	0,000	0,699	0,926	0,739	0,745	132	A:0.409091	G:0.590909	G	G
rs13173618	138139780	T	G	2.89332	SAS	2.71935	0,025	0,163	0,146	0,235	0,220	0,000	0,039	0,005	0,246	0,227				T	T
rs13154573	138148064	T	C	2.78867	SAS	2.65517	0,025	0,163	0,146	0,235	0,220	0,000	0,039	0,005	0,246	0,227				T	T
rs167009	138200457	G	C	-3.24447	AFR	3.82237	0,185	0,004	-0,001	0,235	0,209	0,001	0,785	0,989	0,739	0,768				C	C
rs28363433	138200997	C	T	3.20063	AFR	3.6402	0,086	0,085	0,083	nan	nan	nan	0,088	0,000	0,000	0,000				C	C
rs2292268	138204481	A	G	2.89905	AFR	3.22163	-0,002	0,075	0,005	0,072	0,004	0,041	0,233	0,229	0,086	0,187	222	A:0.855856	G:0.144144	A	A
rs288040	138204894	A	T	3.98994	AFR	3.24117	0,185	0,004	-0,001	0,235	0,209	0,001	0,215	0,011	0,261	0,232				A	A
rs288037	138208441	A	G	3.716	AFR	3.06108	0,178	0,004	-0,001	0,227	0,201	0,001	0,215	0,015	0,261	0,232				A	A
rs288036	138210895	C	A	3.716	AFR	2.97346	0,185	0,004	-0,001	0,235	0,211	0,000	0,215	0,011	0,261	0,234				C	C
rs73790375	138212294	C	G	3.08196	AFR	3.04354	0,056	0,166	0,109	0,058	0,014	0,024	0,170	0,060	0,000	0,025				C	C
rs288035	138212417	A	T	3.71865	AFR	3.08818	0,185	0,004	-0,001	0,235	0,209	0,001	0,215	0,011	0,261	0,232				A	A
.	138213168	C	T	3.27612	AFR	3.12513	0,088	0,086	0,085	nan	nan	nan	0,090	0,000	0,000	0,000				C	C
rs116775683	138213866	T	A	3.2797	AFR	3.24551	0,088	0,086	0,085	nan	nan	nan	0,090	0,000	0,000	0,000				T	T
rs288032	138215328	G	A	3.6793	AFR	3.1761	0,185	0,004	-0,001	0,235	0,209	0,001	0,215	0,011	0,261	0,232				G	G
rs58416876	138215483	G	A	3.19474	AFR	3.19583	0,054	0,054	0,052	nan	nan	nan	0,056	0,000	0,000	0,000				G	G
rs57948908	138215910	T	C	3.19942	AFR	3.18479	0,054	0,164	0,108	0,058	0,014	0,024	0,169	0,060	0,000	0,025				T	T
.	138216625	G	GA	3.73594	AFR	2.32512	0,056	0,031	0,015	0,154	0,125	0,001	0,161	0,054	0,266	0,234				G	G
.	138216635	A	AC	3.01273	AFR	1.37615	0,088	0,086	0,085	nan	nan	nan	0,090	0,000	0,000	0,000				A	A
rs288029	138217305	T	C	3.9892	AFR	2.84692	0,095	0,056	0,037	0,235	0,209	0,001	0,125	0,011	0,261	0,232				T	T
rs288028	138221989	G	C	3.09401	AFR	2.93367	0,185	0,004	-0,001	0,235	0,209	0,001	0,215	0,011	0,261	0,232				G	G
rs288026	138222594	A	G	3.09401	AFR	2.93726	0,185	0,004	-0,001	0,235	0,209	0,001	0,215	0,011	0,261	0,232				A	A
rs288024	138223792	A	G	3.0956	AFR	3.13978	0,185	0,004	-0,001	0,235	0,209	0,001	0,215	0,011	0,261	0,232				A	A
rs288021	138224283	C	T	3.09816	AFR	3.31849	0,185	0,004	-0,001	0,235	0,209	0,001	0,215	0,011	0,261	0,232				C	C
rs76723662	138227636	G	A	3.28271	AFR	3.22947	0,038	0,144	0,088	0,058	0,014	0,024	0,148	0,060	0,000	0,025				G	G
rs288009	138228662	G	A	2.96765	AFR	3.2134	0,185	0,004	-0,001	0,235	0,209	0,001	0,215	0,011	0,261	0,232				G	G
rs111232820	138229204	A	G	3.10605	AFR	3.2747	0,061	0,172	0,115	0,058	0,014	0,024	0,177	0,060	0,000	0,025				A	A
.	138231192	TG	T	-2.84924	AFR	1.03381	0,458	0,188	0,192	0,123	0,121	-0,002	0,590	0,075	0,269	0,266				T	T
.	138232773	G	GC	-3.14591	AFR	1.96327	0,389	0,180	0,171	0,071	0,078	-0,002	0,662	0,176	0,347	0,356				G	G
rs423469	138234116	G	C	-3.21844	AFR	3.30143	0,470	0,275	0,279	0,062	0,060	-0,002	0,666	0,123	0,266	0,262				C	C
rs10043722	138236941	A	G	-3.15695	AFR	3.2609	0,387	0,178	0,165	0,071	0,081	-0,001	0,661	0,176	0,347	0,359				G	G
rs113680826	138249584	G	T	2.96137	AFR	3.2686	0,107	0,106	0,103	nan	nan	nan	0,109	0,000	0,000	0,000				G	G
rs144366481	71216945	T	G	2.85518	AFR	0.62653	0,054	0,054	0,052	nan	nan	nan	0,056	0,000	0,000	0,000				T	T
rs114243388	71221038	C	A	3.12879	AFR	0.997642	0,080	0,079	0,077	nan	nan	nan	0,082	0,000	0,000	0,000				C	C
rs76431592	71233067	T	C	3.67489	AFR	1.67724	0,081	0,080	0,079	nan	nan	nan	0,084	0,000	0,000	0,000				T	T
rs73288536	71233143	G	A	3.01681	AFR	1.70535	0,125	0,123	0,121	nan	nan	nan	0,127	0,000	0,000	0,000				G	G
rs74523703	71233371	T	C	3.19679	AFR	1.70851	0,113	0,112	0,110	nan	nan	nan	0,116	0,000	0,000	0,000				T	T
rs138384029	71234175	C	A	3.54427	AFR	1.64811	0,141	0,139	0,136	nan	nan	nan	0,143	0,000	0,000	0,000				C	C
rs111719427	71234264	T	C	3.54427	AFR	1.64991	0,141	0,139	0,136	nan	nan	nan	0,143	0,000	0,000	0,000				T	T
rs80039445	71234845	G	A	4.48333	AFR	1.60741	0,081	0,080	0,079	nan	nan	nan	0,084	0,000	0,000	0,000				G	G
rs75543073	71235491	G	A	3.57532	AFR	1.62099	0,139	0,137	0,135	nan	nan	nan	0,141	0,000	0,000	0,000				G	G
rs55995694	71236102	C	T	3.44276	AFR	1.34197	0,030	0,000	0,008	0,020	0,069	0,016	0,068	0,016	0,056	0,107				C	C
rs75065769	71237437	A	G	3.35305	AFR	0.938158	0,099	0,098	0,096	nan	nan	nan	0,101	0,000	0,000	0,000				A	A

rs16936922	71239363	C	T	3.16295/2.91292	AFR EUR	0.737432	0,079	-0,001	0,000	0,066	0,059	-0,002	0,076	0,221	0,086	0,092	118	C:0.864407	T:0.135593	C	C
rs10504472	71240478	C	T	3.16222/3.09691	AFR EUR	0.800088	0,014	-0,002	-0,001	0,015	0,010	-0,001	0,076	0,129	0,074	0,083				C	C
rs1586508779	71241921	GATAA	G	3.37178/3.09905	AFR EUR	1.95388	0,013	-0,002	-0,001	0,015	0,010	-0,001	0,077	0,129	0,074	0,083				G	G
rs78388270	71243381	G	A	3.85163	AFR	1.41243	0,112	0,110	0,108	nan	nan	nan	0,114	0,000	0,000	0,000				G	G
.	71243496	AAAAC	A	2.86792	AFR	1.95357	0,116	0,115	0,113	nan	nan	nan	0,119	0,000	0,000	0,000				A	A
rs10217005	71244150	T	A	4.04749	AFR	1.5358	0,054	0,053	0,052	nan	nan	nan	0,056	0,000	0,000	0,000				T	T
rs75603603	71244263	T	C	3.1849	AFR	1.68338	0,141	0,134	0,136	0,000	nan	0,000	0,143	0,000	0,002	0,000				T	T
rs11777228	71245290	G	A	2.9132	EUR	1.5401	0,046	0,060	0,089	0,000	0,010	0,003	0,000	0,047	0,061	0,086				G	G
rs112333542	71245404	A	G	3.1849	AFR	1.65762	0,141	0,139	0,136	nan	nan	nan	0,143	0,000	0,000	0,000				A	A
rs756551488	71246139	C	CTCCT	4.88139	AFR	2.09523	0,080	0,079	0,077	nan	nan	nan	0,082	0,000	0,000	0,000				C	C
rs73288540	71246569	C	G	4.41634	AFR	3.946553	0,112	0,110	0,108	nan	nan	nan	0,114	0,000	0,000	0,000				C	C
rs66548983	71246718	G	A	4.17392	AFR	4.072363	0,075	0,030	-0,001	0,013	0,063	0,021	0,122	0,020	0,051	0,107				G	G
rs73288542	71246784	C	T	4.52719	AFR	3.930703	0,112	0,110	0,108	nan	nan	nan	0,114	0,000	0,000	0,000				C	C
rs35045592	71247253	T	G	-2.75248	AFR	3.931013	0,004	0,238	0,025	0,295	0,053	0,125	0,637	0,686	0,269	0,523				G	G
rs57462399	71247613	G	A	3.29772	AFR	1.74062	0,116	0,115	0,113	nan	nan	nan	0,119	0,000	0,000	0,000				G	G
rs79433673	71247785	C	A	3.12327	AFR	1.58794	0,142	0,140	0,138	nan	nan	nan	0,145	0,000	0,000	0,000				C	C
rs75587782	71248005	T	C	3.15015	AFR	1.44366	0,141	0,139	0,136	nan	nan	nan	0,143	0,000	0,000	0,000				T	T
rs78768200	71249114	G	T	3.65197	AFR	1.36541	0,099	0,098	0,096	nan	nan	nan	0,101	0,000	0,000	0,000				G	G
rs557591972	71249278	GTTTATA	G	4.14579	AFR	1.72505	0,069	0,068	0,066	nan	nan	nan	0,071	0,000	0,000	0,000				G	G
	71250363	T	TAA	4.39371	AFR	1.23011	0,081	0,080	0,079	nan	nan	nan	0,084	0,000	0,000	0,000				T	T
rs115740408	71252006	G	C	4.10193	AFR	1.51072	0,107	0,105	0,103	nan	nan	nan	0,109	0,000	0,000	0,000				G	G
rs117782794	71252037	T	C	3.0558	AFR	1.43396	0,009	0,000	-0,002	0,017	0,012	-0,001	0,088	0,134	0,074	0,083				T	T
rs61194854	71252189	G	C	3.17283	AFR	1.5046	0,073	0,072	0,071	nan	nan	nan	0,076	0,000	0,000	0,000				G	G
rs74546406	71254676	T	C	2.85114	AFR	1.48924	0,141	0,139	0,136	nan	nan	nan	0,143	0,000	0,000	0,000				T	T
rs11993436	71255607	C	G	4.24153	AFR	1.6247	0,108	0,107	0,105	nan	nan	nan	0,111	0,000	0,000	0,000				C	C
rs11453909	71255608	C	CA	2.8909	AFR	1.58794	0,113	0,112	0,110	nan	nan	nan	0,116	0,000	0,000	0,000				C	C
rs79293802	71256037	C	A	3.66061	AFR	1.49002	0,051	0,055	0,054	0,000	0,000	nan	0,058	0,002	0,000	0,000				C	C
rs10112498	71257142	T	G	4.3232	AFR	2.930010	0,103	0,360	0,145	0,119	0,003	0,081	0,106	0,294	0,544	0,338				T	T
rs76565478	71257323	C	T	2.85882	AFR	1.44368	0,141	0,139	0,136	nan	nan	nan	0,143	0,000	0,000	0,000				C	C
rs6472518	71258201	T	C	2.92893	AFR	1.33086	0,113	0,112	0,110	nan	nan	nan	0,116	0,000	0,000	0,000				T	T
rs55794475	71259536	G	A	2.99667	AFR	1.72505	0,113	0,112	0,110	nan	nan	nan	0,116	0,000	0,000	0,000				G	G
rs79495809	71260518	C	A	3.63756	AFR	1.50005	0,069	0,068	0,066	nan	nan	nan	0,071	0,000	0,000	0,000				C	C
rs77672497	71260647	T	C	4.54373	AFR	1.37792	0,080	0,079	0,077	nan	nan	nan	0,082	0,000	0,000	0,000				T	T
rs1870649	71260985	C	G	-3.50358	AFR	3.207244	0,098	0,503	0,169	0,254	0,011	0,171	0,889	0,706	0,323	0,630				G	G
rs58356753	71261083	C	T	4.439	AFR	1.35505	0,080	0,079	0,077	nan	nan	nan	0,082	0,000	0,000	0,000				C	C
.	71261262	AG	A	2.80296	AFR	1.11321	0,096	0,095	0,093	nan	nan	nan	0,098	0,000	0,000	0,000				A	A
rs10099636	71264008	T	A	3.3495	AFR	1.04447	0,066	0,023	-0,001	0,013	0,058	0,017	0,116	0,021	0,054	0,106				T	T
rs73288551	71268207	G	T	3.73657	AFR	1.403	0,128	0,126	0,124	nan	nan	nan	0,130	0,000	0,000	0,000				G	G
rs73684284	71269293	T	C	2.83504	AFR	1.48867	0,113	0,112	0,110	nan	nan	nan	0,116	0,000	0,000	0,000				T	T
rs16936942	71273368	T	C	3.29289	AFR	1.70038	0,019	-0,002	-0,001	0,017	0,012	-0,001	0,072	0,134	0,074	0,083				T	T
rs114036699	71273400	T	A	2.99777	AFR	1.67124	0,057	0,057	0,055	nan	nan	nan	0,059	0,000	0,000	0,000				T	T
rs11985355	71273481	T	C	3.85072	AFR	1.55586	0,123	0,121	0,119	nan	nan	nan	0,125	0,000	0,000	0,000				T	T
rs73288553	71276195	T	G	3.71034	AFR	3.918418	0,129	0,128	0,125	nan	nan	nan	0,132	0,000	0,000	0,000				T	T
rs1227276	71277079	T	C	3.89515	AFR	1.74984	0,105	0,100	0,102	0,000	nan	0,000	0,108	0,000	0,002	0,000				T	C
rs16936953	71281666	T	C	3.28069	AFR	1.47911	0,158	0,156	0,153	nan	nan	nan	0,161	0,000	0,000	0,000				T	C
rs115339496	71283948	G	A	3.82201	AFR	1.32849	0,080	0,079	0,077	nan	nan	nan	0,082	0,000	0,000	0,000				G	G
rs73288557	71286090	T	G	3.15576	AFR	1.25341	0,129	0,128	0,125	nan	nan	nan	0,132	0,000	0,000	0,000				T	T
rs73288558	71286897	G	A	3.32344	AFR	1.25946	0,113	0,112	0,110	nan	nan	nan	0,116	0,000	0,000	0,000				G	G
	71287222	GA	G	3.5752	AFR	1.51329	0,102	0,101	0,099	nan	nan	nan	0,105	0,000	0,000	0,000				G	G
rs116638669	71289016	C	T	3.92915	AFR	1.40987	0,080	0,079	0,077	nan	nan	nan	0,082	0,000	0,000	0,000				C	C
rs56016016	71291178	C	G	3.1564	AFR	1.56491	0,054	0,054	0,052	nan	nan	nan	0,056	0,000	0,000	0,000				C	C
rs8069478	19553248	G	T	3.00031	AFR	-3.23234	0,487	0,012	0,117	0,406	0,241	0,055	0,521	0,016	0,438	0,275				G	G
rs4925036	19553659	A	G	2.99963	AFR	-3.21356	0,487	0,008	0,115	0,418	0,243	0,061	0,521	0,016	0,449	0,276				A	A

rs962800	19559468	A	G	-3.07148	AFR	-2.98766	0,487	0,008	0,114	0,418	0,245	0,060	0,479	0,984	0,551	0,722	292	A:0.390411	G:0.609589	G	G
.	19562061	TA	T	-3.04805	AFR	-1.15574	0,395	0,002	0,092	0,353	0,172	0,061	0,498	0,944	0,540	0,715				T	T
rs2386145	19563018	C	G	-2.93033	AFR	-3.05574	0,487	0,008	0,114	0,418	0,245	0,060	0,479	0,984	0,551	0,722				G	G
rs2108971	19565945	G	A	-2.80531	AFR	-3.20382	0,487	0,008	0,115	0,418	0,243	0,061	0,479	0,984	0,551	0,724	130	G:0.323077	A:0.676923	A	A
rs59755039	19568486	CT	C	-2.85284	AFR	-3.41374	0,487	0,008	0,115	0,418	0,243	0,061	0,479	0,984	0,551	0,724				C	C
rs8069576	19570320	A	G	-3.06948	AFR	-3.10700	0,487	0,008	0,115	0,418	0,243	0,061	0,479	0,984	0,551	0,724				G	G

Table S2. continued

Allele	POS	Genetic region	Gtex/eGene	RegulomeDB		chromatin state	motif	LD			GWAS Catalog
				Score	Rank			Gene variant	R2	population	
rs1579513969	100238002	intron_variant,genic_upstream_transcript_variant									
rs72896632	110354973	non_coding_transcript_variant,genic_upstream_transcript_variant,5_prime_UTR_variant	SEC24B, SEC24B-AS1, RBMXP4	0.82368	1b	Active TSS	E2F6, EWSR1				
rs10000545	110357878	genic_upstream_transcript_variant,intron_variant	SEC24B, SEC24B-AS1, RBMXP4, SETP20	0.66703	1f	Weak enhancer, Weak transcription, Quiescent Low	BCL6, ISL1, STAT4STAT5A, STAT5B				
rs11942858	110358103	genic_upstream_transcript_variant,intron_variant	SEC24B, SEC24B-AS1, RBMXP4	0.83785	1f	Weak enhancer, Weak transcription, Quiescent Low	ZKSCAN5				
rs11933971	110358288	genic_upstream_transcript_variant,intron_variant	SEC24B, SEC24B-AS1, RBMXP4	0.55324	1f	Weak enhancer, Weak transcription, Quiescent Low	HNF4A, HNF4G				
rs34836476	110361998	ntron_variant,genic_upstream_transcript_variant	SEC24B, SEC24B-AS1, RBMXP4	0.55436	1f	Weak enhancer, Weak transcription, Quiescent Low	LIN54				
rs13103034	110362373	genic_upstream_transcript_variant,intron_variant	SEC24B, SEC24B-AS1, RBMXP4	0.55436	1f	Weak enhancer, Weak transcription, Quiescent Low					
rs6817954	110362752	genic_upstream_transcript_variant,intron_variant	SEC24B, SEC24B-AS1, RBMXP4	0.22271	1f	Weak enhancer, Weak transcription, Quiescent Low					
.	110365370										
rs17040436	110365426	intron_variant,genic_upstream_transcript_variant	SEC24B, SEC24B-AS1, RBMXP4	1.0	1b	Weak enhancer, Weak transcription					
rs17040439	110365515	intron_variant,genic_upstream_transcript_variant	SEC24B, SEC24B-AS1, RBMXP4	0.55436	1f	Weak enhancer, Weak transcription, Quiescent Low					
.	110365628										
.	110373232										
rs76264283	110373544	intron_variant,genic_upstream_transcript_variant		0.13454	5	Weak enhancer, Weak transcription, Quiescent Low					
rs10009463	110373682	genic_upstream_transcript_variant,intron_variant		0.51392	7	Weak transcription, Quiescent Low					
.	110375694										
rs28597954	110376162	intron_variant,genic_upstream_transcript_variant		0.55436	1f	Weak transcription, Quiescent Low					
rs28663368	110376396	intron_variant,genic_upstream_transcript_variant		0.22271	1f	Weak transcription, Quiescent Low					
rs112585058	110378131	intron_variant,genic_upstream_transcript_variant		0.23589	5	Weak transcription, Quiescent Low					
rs34132975	110378352	intron_variant,genic_upstream_transcript_variant	SEC24B, SEC24B-AS1, RBMXP4	0.22271	1f	Strong transcription, Weak transcription	KLF1, KLF2, KLF6, KLF9				
rs77911134	110379266	intron_variant,genic_upstream_transcript_variant	RBMXP4	0.51392	7	Strong transcription, Weak transcription					
rs79567241	110379367	intron_variant,genic_upstream_transcript_variant		0.08912	6	Weak transcription, Quiescent Low, strong transcription					
rs76784285	110379670	intron_variant,genic_upstream_transcript_variant	RBMXP4	0.55324	1f	Weak transcription, Quiescent Low, strong transcription					
.	110381786										
rs7697001	110381952	genic_upstream_transcript_variant,intron_variant		0.58766	6	Weak transcription, Quiescent Low, strong transcription	TEAD1, TEAD2				
rs78653746	110382720	intron_variant,genic_upstream_transcript_variant		0.13454	5	Weak transcription, Quiescent Low					
rs28680423	110385187	intron_variant,genic_upstream_transcript_variant		0.51392	7	Weak transcription, Quiescent Low					
rs6840561	110385601	genic_upstream_transcript_variant,intron_variant		0.08	1f	Weak transcription, Quiescent Low, weak enhancer, Active enhancer					

rs17040454	110385758	intron_variant,genic_upstream_transcript_variant	RBMXP4	0.51392	7	Weak transcription, Quiescent Low, strong transcription					
rs12643881	110386443	genic_upstream_transcript_variant,intron_variant	RBMXP4	0.22271	1f	Weak transcription, Quiescent Low, strong transcription					
rs113756500	110387074	intron_variant,genic_upstream_transcript_variant		0.18412	7	Weak transcription, Quiescent Low	ZFX				
rs28412073	110387569	intron_variant,genic_upstream_transcript_variant		0.81357	5	Weak transcription, Quiescent Low, strong transcription					
rs77573441	110387757	intron_variant,genic_upstream_transcript_variant	SEC24B, SEC24B-AS1, RBMXP4	0.55436	1f	Weak transcription, Quiescent Low	NFIC				
.	110388248										
rs184928998	110388254	intron_variant,genic_upstream_transcript_variant		0.63796	2b	Weak transcription, Quiescent Low					
rs10706780	110389283	genic_upstream_transcript_variant,intron_variant		0.51392	7	Weak transcription, Quiescent Low, strong transcription	IRF1, RREB1, SOX3				
rs34813537	110389580	intron_variant,genic_upstream_transcript_variant	SEC24B, SEC24B-AS1, RBMXP4	0.22271	1f	Weak transcription, Quiescent Low, strong transcription					
rs74761343	110390687	intron_variant,genic_upstream_transcript_variant		0.18412	7	Weak transcription, Quiescent Low	FOXD3, FOXL1				
rs112761722	110390833	intron_variant,genic_upstream_transcript_variant		0.0	6	Weak transcription, Quiescent Low	ARID5A, DMBX1				
rs7681265	110391962	genic_upstream_transcript_variant,intron_variant		0.03154	1f	Weak transcription, Quiescent Low, strong transcription	POU5F1				
rs10606064	110392793	genic_upstream_transcript_variant,intron_variant		0.22271	1f	Weak transcription, Quiescent Low, strong transcription					
rs182286635	110392795	intron_variant,genic_upstream_transcript_variant		0.22271	1f	Weak transcription, Quiescent Low					
rs17040466	110393650	intron_variant,genic_upstream_transcript_variant		0.55436	1f	Strong tansciption, Weak transcription					
rs67518796	110393749	intron_variant,genic_upstream_transcript_variant	SEC24B, SEC24B-AS1, RBMXP4	1.0	1b	Strong tansciption, Weak transcription					
rs10516556	110395146	genic_upstream_transcript_variant,intron_variant	PLA2G12A 6,3emoins8	0.22271	1f	Weak transcription, Quiescent Low, strong transcription					
rs10516557	110395406	genic_upstream_transcript_variant,intron_variant		0.22271	1f	Weak transcription, Quiescent Low, strong transcription					
rs78262578	110396045	intron_variant,genic_upstream_transcript_variant		0.58955	5	Weak transcription, Quiescent Low, strong transcription					
.	110397146										
.	110397721										
rs34320670	110397724	intron_variant,genic_upstream_transcript_variant	SEC24B, SEC24B-AS1, RBMXP4	0.51392	7	Weak transcription, Quiescent Low, strong transcription					
rs28580356	110398287	intron_variant,genic_upstream_transcript_variant		0.51392	7	Weak transcription, Quiescent Low, strong transcription					
rs28541279	110398890	intron_variant,genic_upstream_transcript_variant		0.51392	7	Weak transcription, Quiescent Low					
rs35866656	110399231	intron_variant,genic_upstream_transcript_variant	SEC24B, SEC24B-AS1, RBMXP4	0.51392	7	Weak transcription, Quiescent Low					
rs6851756	110399303	genic_upstream_transcript_variant,intron_variant		0.18412	7	Weak transcription, Quiescent Low					
.	110399441										
rs28460762	110399613	intron_variant,genic_upstream_transcript_variant		0.51392	7	Weak transcription, Quiescent Low					
rs700626	138117938	genic_upstream_transcript_variant,intron_variant	AC034243.1, CTNNA1, SIL1	0.22271	1f	Flanking TSS upstream, Active TSS, Weak transcription, Enhancers	IRF3				
rs13173618	138139780	intron_variant,genic_upstream_transcript_variant	AC034243.1, CTNNA1, SIL1	0.55436	1f	Weak transcription, Quiescent Low, Enhancers	VDR, ZNF135, ZNF164	MAOB43685616	1	EAS	
rs13154573	138148064	intron_variant,genic_upstream_transcript_variant	AC034243.1, CTNNA1, SIL1	0.55324	1f	Strong transcription, Weak transcription, Quiescent Low		MAOB43685617	1	EAS	
rs167009	138200457	genic_upstream_transcript_variant,intron_variant	AC034243.1, CTNNA1, SIL1	0.14583	6	Quiescent Low, Weak transcription, Weak Enhancer		MAOB43685618	1	EAS	
rs28363433	138200997	genic_upstream_transcript_variant,intron_variant		0.85691	2b	Strong transcription, Weak transcription, Quiescent Low					
rs2292268	138204481	500B_downstream_variant,genic_upstream_transcript_variant,intron_variant,downstream_transcript_variant		0.55324	1f	Quiescent Low, Weak transcription, Weak Enhancer					
rs288040	138204894	3_prime_UTR_variant,genic_upstream_transcript_variant,intron_variant		0.55436	1f	Quiescent Low, Weak transcription, Weak Enhancer		MAOB43685618	1	EAS	

rs288037	138208441	3_prime_UTR_variant,genic_upstream_transcript_variant,intron_variant	AC034243.1, CTNNA1, SIL1	0.22271	1f	Quiescent Low, Weak transcription, Weak Enhancer, TSS		MAOB43685618	1	EAS	
rs288036	138210895	genic_upstream_transcript_variant,5_prime_UTR_variant,intron_variant,upstream_transcript_variant	AC034243.1, CTNNA1, SIL1	0.64348	1b	Weak transcription		MAOB43685618	1	EAS	
rs73790375	138212294	2KB_upstream_variant,intron_variant,upstream_transcript_variant	AC034243.1	0.51392	7	Weak transcription, Quiescent Low					
rs288035	138212417	intron_variant,2KB_upstream_variant,upstream_transcript_variant	AC034243.1, CTNNA1, SIL1	0.22271	1f	Weak transcription, Quiescent Low		MAOB43685618	1	EAS	
.	138213168										
rs116775683	138213866	intron_variant		0.0	5	Weak transcription, Quiescent Low	EHF, ELF1, ETV1, GABPA				
rs288032	138215328	intron_variant	AC034243.1, CTNNA1, SIL1	0.22271	1f	Weak transcription, Quiescent Low, Strong transcription		MAOB43685618	1	EAS	
rs58416876	138215483	intron_variant		0.22365	6	Weak transcription, Quiescent Low, Strong transcription					
rs57948908	138215910	5_prime_UTR_variant,intron_variant	AC034243.1	0.55436	1f	Quiescent Low, Weak transcription	ZNF423				
.	138216625										
.	138216635										
rs288029	138217305	intron_variant	AC034243.1, CTNNA1, SIL1	0.71	1f	Quiescent Low, Weak transcription, Weak Enhancer	NFE2L2	MAOB43685618	1	EAS	acute myeloid leukemia
rs288028	138221989	intron_variant	AC034243.1, CTNNA1, SIL1	0.51392	7	Weak transcription, Quiescent Low, Strong transcription		MAOB43685618	1	EAS	acute myeloid leukemia
rs288026	138222594	intron_variant	AC034243.1, CTNNA1, SIL1	0.51392	7	Quiescent Low, Weak transcription, Heterochromatin	OSR2	MAOB43685618	1	EAS	
rs288024	138223792	intron_variant	AC034243.1, CTNNA1, SIL1	0.51392	7	Weak transcription, Quiescent Low	FOXD1, FOXI1, FOXL1, FOXO4	MAOB43685618	1	EAS	
rs288021	138224283	intron_variant	AC034243.1, CTNNA1, SIL1	0.51392	7	Weak transcription, Quiescent Low, Strong transcription	BHLHA15, MEIS1, POU2F2	MAOB43685618	1	EAS	
rs76723662	138227636	intron_variant	AC034243.1	0.80877	6	Weak transcription, Quiescent Low, Strong transcription	INSM1				
rs288009	138228662	intron_variant	AC034243.1, CTNNA1, SIL1	0.55324	1f	Weak transciption, Enhancers, TSS		MAOB43685618	1	EAS	
rs111232820	138229204	intron_variant	AC034243.1	0.14083	6	Weak transcription, Quiescent Low, Strong transcription, Enhancers		rs2147154680			
.	138231192										
.	138232773										
rs423469	138234116	intron_variant	AC034243.1, CTNNA1, SIL1	0.22271	1f	Weak transcription, Quiescent Low, Strong transcription					
rs10043722	138236941	intron_variant	AC034243.1, CTNNA1, SIL1	0.94667	1b	Strong transcription, Weak transcription, Quiescent Low					risk-taking behaviour, Insomnia
rs113680826	138249584	intron_variant		0.13454	5	Strong transcription, Weak transcription, Quiescent Low					
rs144366481	71216945	genic_upstream_transcript_variant,intron_variant		0.60906	4	Quiescent Low, Weak transcription, Enhancers	OSR2				
rs114243388	71221038	intron_variant,genic_upstream_transcript_variant		0.64596	5	Quiescent Low, Weak transcription, Enhancers	MAFK				
rs76431592	71233067	intron_variant,genic_upstream_transcript_variant		0.13454	5	Quiescent Low, Weak transcription, Enhancers, Flanking TSS downstream					
rs73288536	71233143	intron_variant,genic_upstream_transcript_variant		0.13454	5	Quiescent Low, Weak transcription, Enhancers	BACH2, BATF, FOS,				
rs74523703	71233371	intron_variant,genic_upstream_transcript_variant		0.18412	7	Quiescent Low, Weak transcription, Enhancers, TSS	EWSR1				
rs138384029	71234175	genic_upstream_transcript_variant,intron_variant		0.63479	5	Quiescent Low, Weak transcription, Enhancers					
rs111719427	71234264	intron_variant,genic_upstream_transcript_variant		0.52415	5	Quiescent Low, Weak transcription, Enhancers					
rs80039445	71234845	intron_variant,genic_upstream_transcript_variant		0.13454	5	Quiescent Low, Weak transcription, Enhancers					
rs75543073	71235491	intron_variant,genic_upstream_transcript_variant		0.60906	4	Quiescent Low, Weak transcription, Enhancers, TSS					
rs55995694	71236102	intron_variant,genic_upstream_transcript_variant		0.13454	5	Quiescent Low, Weak transcription, Enhancers, TSS					

rs75065769	71237437	intron_variant,genic_upstream_transcript_variant		0.60906	4	Quiescent Low, Weak transcription, Enhancers, TSS					
rs16936922	71239363	intron_variant,genic_upstream_transcript_variant		0.02937	1f	Quiescent Low, Weak transcription, Enhancers, TSS					body height, body mass index
rs10504472	71240478	genic_upstream_transcript_variant,upstream_transcript_variant,intron_variant		0.60906	4	Quiescent Low, Weak transcription, Enhancers, TSS					
rs1586508779	71241921	genic_upstream_transcript_variant,intron_variant,upstream_transcript_variant		0.58955	5	Quiescent Low, Weak transcription, Enhancers, Heterochromatin					
rs78388270	71243381	intron_variant,genic_upstream_transcript_variant		0.60906	4	Quiescent Low, Weak transcription, Enhancers, Heterochromatin					
.	71243496										
rs10217005	71244150	genic_upstream_transcript_variant,intron_variant		0.60906	4	Quiescent Low, Weak transcription, Enhancers, Heterochromatin, Flanking TSS upstream					
rs75603603	71244263	intron_variant,genic_upstream_transcript_variant		0.60906	4	Quiescent Low, Weak transcription, Enhancers					
rs11777228	71245290	genic_upstream_transcript_variant,intron_variant		0.60906	4	Quiescent Low, Weak transcription, Enhancers					
rs112333542	71245404	intron_variant,genic_upstream_transcript_variant		0.86817	2b	Quiescent Low, Weak transcription, Enhancers					
rs756551488	71246139	genic_upstream_transcript_variant,intron_variant		0.13454	5	Quiescent Low, Weak transcription, Enhancers					
rs73288540	71246569	intron_variant,genic_upstream_transcript_variant		0.13454	5	Quiescent Low, Weak transcription, Enhancers					
rs66548983	71246718	intron_variant,genic_upstream_transcript_variant		0.13454	5	Quiescent Low, Weak transcription, Enhancers, Flanking TSS upstream					
rs73288542	71246784	intron_variant,genic_upstream_transcript_variant		0.66703	1f	Quiescent Low, Weak transcription, Enhancers, Flanking TSS upstream					
rs35045592	71247253	intron_variant,genic_upstream_transcript_variant		0.60906	4	Quiescent Low, Weak transcription, Enhancers, Heterochromatin					
rs57462399	71247613	intron_variant,genic_upstream_transcript_variant		0.60906	4	Quiescent Low, Weak transcription, Enhancers, Heterochromatin					
rs79433673	71247785	intron_variant,genic_upstream_transcript_variant		0.60906	4	Quiescent Low, Weak transcription, Enhancers					
rs75587782	71248005	intron_variant,genic_upstream_transcript_variant		0.26465	6	Quiescent Low, TSS, Weak transcription, Enhancers					
rs78768200	71249114	intron_variant,genic_upstream_transcript_variant		0.18412	7	Quiescent Low, Weak transcription, Enhancers, TSS	PBX2				
rs557591972	71249278	genic_upstream_transcript_variant,intron_variant		0.18412	7	Quiescent Low, Weak transcription, Enhancers, Flanking TSS upstream					
	71250363			0.18412	7	Quiescent Low, Weak transcription, Enhancers	GFI1B, RUNX1				
rs115740408	71252006	intron_variant,genic_upstream_transcript_variant		0.60906	4	Quiescent Low, Weak transcription, Enhancers					
rs117782794	71252037	genic_upstream_transcript_variant,intron_variant		0.58955	5	Quiescent Low, Weak transcription, Enhancers					
rs61194854	71252189	intron_variant,genic_upstream_transcript_variant		0.58955	5	Quiescent Low, Weak transcription, Enhancers	FOXP3				
rs74546406	71254676	intron_variant,genic_upstream_transcript_variant		0.18412	7	Quiescent Low, Weak transcription, Enhancers	ZNF140				
rs11993436	71255607	intron_variant,genic_upstream_transcript_variant		0.60906	4	Quiescent Low, Weak transcription, Enhancers					
rs11453909	71255608	genic_upstream_transcript_variant,intron_variant		0.60906	4	Quiescent Low, Weak transcription, Enhancers					
rs79293802	71256037	intron_variant,genic_upstream_transcript_variant		0.60906	4	Quiescent Low, Weak transcription, Enhancers, Flanking TSS upstream		MAOB43650108	1	AFR	
rs10112498	71257142	genic_upstream_transcript_variant,intron_variant		0.58955	5	Quiescent Low, Weak transcription, Enhancers, Flanking TSS upstream, Heterochromatin					
rs76565478	71257323	intron_variant,genic_upstream_transcript_variant		0.60906	4	Quiescent Low, Weak transcription, Enhancers, Heterochromatin					
rs6472518	71258201	genic_upstream_transcript_variant,intron_variant		0.60906	4	Quiescent Low, Weak transcription, Enhancers, Heterochromatin					
rs55794475	71259536	intron_variant,genic_upstream_transcript_variant		0.55436	1f	Quiescent Low, Weak transcription, Enhancers					
rs79495809	71260518	intron_variant,genic_upstream_transcript_variant		0.60906	4	Quiescent Low, Weak transcription, Enhancers					
rs77672497	71260647	intron_variant,genic_upstream_transcript_variant		0.88556	5	Quiescent Low, Weak transcription, Enhancers					

rs1870649	71260985	genic_upstream_transcript_variant,intron_variant		0.18412	7	Quiescent Low, Weak transcription, Weak Enhancer	ZNF384				
rs58356753	71261083	intron_variant,genic_upstream_transcript_variant		0.13454	5	Quiescent Low, Weak transcription, Weak Enhancer, Heterochromatin					
.	71261262										
rs10099636	71264008	genic_upstream_transcript_variant,intron_variant		0.60906	4	Quiescent Low, Weak transcription, Enhancers, Flanking TSS upstream, Heterochromatin	THAP11				
rs73288551	71268207	intron_variant,genic_upstream_transcript_variant		0.60906	4	Quiescent Low, Weak transcription, Enhancers	IRF1, NFATC2, ZNF384				
rs73684284	71269293	intron_variant,genic_upstream_transcript_variant		0.81512	2a	Quiescent Low, Weak transcription, Enhancers, Flanking TSS downstream					
rs16936942	71273368	intron_variant,genic_upstream_transcript_variant		0.01	5	Quiescent Low, Weak transcription, Heterochromatin	MSANTD3				
rs114036699	71273400	intron_variant,genic_upstream_transcript_variant		0.00125	5	Quiescent Low, Weak transcription, Weak Enhancer, Heterochromatin	HNF4A				
rs11985355	71273481	intron_variant,genic_upstream_transcript_variant		0.60906	4	Quiescent Low, Weak transcription, Enhancers	ARNT, MLXIP				
rs73288553	71276195	intron_variant,genic_upstream_transcript_variant		0.49716	6	Quiescent Low, Weak transcription, Enhancers, Flanking TSS downstream					
rs1227276	71277079	genic_upstream_transcript_variant,intron_variant		0.58955	5	Quiescent Low, Weak transcription, Weak Enhancer, Heterochromatin					
rs16936953	71281666	intron_variant,genic_upstream_transcript_variant		0.60906	4	Quiescent Low, Weak transcription, Heterochromatin					
rs115339496	71283948	genic_upstream_transcript_variant,intron_variant		0.70497	4	Quiescent Low, Weak transcription, Heterochromatin					
rs73288557	71286090	intron_variant,genic_upstream_transcript_variant		0.60906	4	Quiescent Low, Weak transcription, Enhancers					
rs73288558	71286897	intron_variant,genic_upstream_transcript_variant		0.14	5	Quiescent Low, Weak transcription, Enhancers, Heterochromatin					
	71287222										
rs116638669	71289016	genic_upstream_transcript_variant,intron_variant		0.70497	4	Quiescent Low, Weak transcription, Enhancers, TSS	PBX2				
rs56016016	71291178	intron_variant,genic_upstream_transcript_variant		0.60906	4	Quiescent Low, Weak transcription, Enhancers, Flanking TSS upstream					
allele	POS	Genetic region		Regulome DB		Chromatin state	motif				
rs8069478	19553248	intron_variant,genic_upstream_transcript_variant	RP11-311F12.1, ALDH3A2	0.55436	1f	TSS, Weak transciption, Enhancers, Quiescent Low					
rs4925036	19553659	intron_variant,genic_upstream_transcript_variant	RP11-311F12.1, ALDH3A2	0.66703	1f	TSS, Weak transciption, Enhancers, Quiescent Low	BATF, BATF3, DUXA, FOSL1				
rs962800	19559468	intron_variant	RP11-311F12.1, ALDH3A2	0.66703	1f	Strong transcription, Weak transcription, Enhancers	GATA1				
.	19562061										
rs2386145	19563018	intron_variant	RP11-311F12.1, ALDH3A2	0.55436	1f	Weak transcription, Strong transcription, Quiescent Low, Enhancers					
rs2108971	19565945	intron_variant	RP11-311F12.1, ALDH3A2	0.51392	7	Strong transcription, Weak transcription, Enhancers, Quiescent Low					
rs59755039	19568486	intron_variant	RP11-311F12.1, ALDH3A2	0.55436	1f	Weak transcription, Strong transcription, Quiescent Low, Enhancers	IRF1, STAT2				
rs8069576	19570320	intron_variant	RP11-311F12.1, ALDH3A2	0.51392	7	Strong transcription, Weak transcription, Quiescent Low, Enhancers					

Table S3: Identified eGene for each SNPs, p-values and tissues where these eGenes are expressed. Positive NES values suggest that the alternative allele has high expression relative to the reference allele. Negative NES values indicate the opposite.

Gene	SNP Id	Gencode Id	Gene Symbol	Variant Id	P-Value	NES	Tissue
		ENSG00000138802.11	SEC24B	chr4_109433817_C_T_b38	1.0e-15	0.32	Cells - Cultured fibroblasts
		ENSG00000138802.11	SEC24B	chr4_109433817_C_T_b38	1.4e-11	0.16	Artery - Tibial
		ENSG00000247950.6	SEC24B-AS1	chr4_109433817_C_T_b38	2.8e-10	0.34	Skin - Sun Exposed (Lower leg)
		ENSG00000249465.1	RBMXP4	chr4_109433817_C_T_b38	3.0e-10	0.40	Skin - Not Sun Exposed (Suprapubic)
		ENSG00000249465.1	RBMXP4	chr4_109433817_C_T_b38	6.5e-10	0.38	Skin - Sun Exposed (Lower leg)
		ENSG00000138802.11	SEC24B	chr4_109433817_C_T_b38	3.7e-9	0.41	Ovary
		ENSG00000247950.6	SEC24B-AS1	chr4_109433817_C_T_b38	3.8e-9	0.35	Skin - Not Sun Exposed (Suprapubic)
		ENSG00000138802.11	SEC24B	chr4_109433817_C_T_b38	2.2e-8	0.12	Muscle - Skeletal
		ENSG00000138802.11	SEC24B	chr4_109433817_C_T_b38	6.1e-8	0.16	Esophagus - Mucosa
		ENSG00000138802.11	SEC24B	chr4_109433817_C_T_b38	4.7e-7	0.23	Pancreas
		ENSG00000188517.14	COL25A1	chr4_109433817_C_T_b38	0.0000038	0.41	Brain - Cortex
		ENSG00000138802.11	SEC24B	chr4_109433817_C_T_b38	0.000015	0.11	Adipose - Subcutaneous
		ENSG00000225094.3	SETP20	chr4_109433817_C_T_b38	0.000024	0.31	Skin - Sun Exposed (Lower leg)
	rs72896632	ENSG00000138802.11	SEC24B	chr4_109433817_C_T_b38	0.000077	0.11	Thyroid
		ENSG00000138802.11	SEC24B	chr4_109436722_G_C_b38	8.8e-15	0.30	Cells - Cultured fibroblasts
		ENSG00000249465.1	RBMXP4	chr4_109436722_G_C_b38	9.8e-15	0.50	Skin - Not Sun Exposed (Suprapubic)
		ENSG00000247950.6	SEC24B-AS1	chr4_109436722_G_C_b38	1.5e-13	0.44	Skin - Not Sun Exposed (Suprapubic)
		ENSG00000249465.1	RBMXP4	chr4_109436722_G_C_b38	6.0e-12	0.43	Skin - Sun Exposed (Lower leg)
		ENSG00000138802.11	SEC24B	chr4_109436722_G_C_b38	2.4e-11	0.15	Artery - Tibial
		ENSG00000138802.11	SEC24B	chr4_109436722_G_C_b38	4.8e-10	0.18	Esophagus - Mucosa
		ENSG00000247950.6	SEC24B-AS1	chr4_109436722_G_C_b38	5.1e-10	0.34	Skin - Sun Exposed (Lower leg)
		ENSG00000225094.3	SETP20	chr4_109436722_G_C_b38	7.7e-8	0.39	Skin - Sun Exposed (Lower leg)
		ENSG00000138802.11	SEC24B	chr4_109436722_G_C_b38	4.0e-7	0.11	Muscle - Skeletal
		ENSG00000138802.11	SEC24B	chr4_109436722_G_C_b38	0.0000010	0.21	Pancreas
		ENSG00000138802.11	SEC24B	chr4_109436722_G_C_b38	0.0000011	0.35	Ovary
		ENSG00000005059.15	MCUB	chr4_109436722_G_C_b38	0.0000023	0.17	Nerve - Tibial
		ENSG00000138802.11	SEC24B	chr4_109436722_G_C_b38	0.0000033	0.12	Adipose - Subcutaneous
		ENSG00000225094.3	SETP20	chr4_109436722_G_C_b38	0.000021	0.34	Esophagus - Muscularis
		ENSG00000138802.11	SEC24B	chr4_109436722_G_C_b38	0.000026	0.33	Brain - Hypothalamus
		ENSG00000247950.6	SEC24B-AS1	chr4_109436722_G_C_b38	0.000071	-0.21	Adipose - Subcutaneous
	rs10000545	ENSG00000188517.14	COL25A1	chr4_109436722_G_C_b38	0.000075	0.36	Brain - Cortex
		ENSG00000138802.11	SEC24B	chr4_109436947_A_G_b38	3.9e-13	0.29	Cells - Cultured fibroblasts
		ENSG00000138802.11	SEC24B	chr4_109436947_A_G_b38	4.8e-11	0.15	Artery - Tibial
		ENSG00000247950.6	SEC24B-AS1	chr4_109436947_A_G_b38	2.5e-10	0.37	Skin - Not Sun Exposed (Suprapubic)
		ENSG00000249465.1	RBMXP4	chr4_109436947_A_G_b38	1.7e-9	0.39	Skin - Not Sun Exposed (Suprapubic)
		ENSG00000138802.11	SEC24B	chr4_109436947_A_G_b38	2.2e-9	0.13	Muscle - Skeletal
		ENSG00000249465.1	RBMXP4	chr4_109436947_A_G_b38	2.5e-9	0.37	Skin - Sun Exposed (Lower leg)
		ENSG00000138802.11	SEC24B	chr4_109436947_A_G_b38	2.9e-9	0.41	Ovary
		ENSG00000247950.6	SEC24B-AS1	chr4_109436947_A_G_b38	3.9e-9	0.32	Skin - Sun Exposed (Lower leg)
		ENSG00000138802.11	SEC24B	chr4_109436947_A_G_b38	1.6e-7	0.15	Esophagus - Mucosa
		ENSG00000138802.11	SEC24B	chr4_109436947_A_G_b38	3.0e-7	0.23	Pancreas
		ENSG00000188517.14	COL25A1	chr4_109436947_A_G_b38	7.3e-7	0.43	Brain - Cortex
		ENSG00000138802.11	SEC24B	chr4_109436947_A_G_b38	0.0000026	0.12	Adipose - Subcutaneous
	rs11942858	ENSG00000225094.3	SETP20	chr4_109436947_A_G_b38	0.000014	0.32	Skin - Sun Exposed (Lower leg)
		ENSG00000138802.11	SEC24B	chr4_109437132_C_T_b38	3.9e-13	0.29	Cells - Cultured fibroblasts
		ENSG00000138802.11	SEC24B	chr4_109437132_C_T_b38	2.7e-11	0.15	Artery - Tibial
		ENSG00000247950.6	SEC24B-AS1	chr4_109437132_C_T_b38	2.5e-10	0.37	Skin - Not Sun Exposed (Suprapubic)
		ENSG00000138802.11	SEC24B	chr4_109437132_C_T_b38	1.6e-9	0.13	Muscle - Skeletal
		ENSG00000249465.1	RBMXP4	chr4_109437132_C_T_b38	1.7e-9	0.39	Skin - Not Sun Exposed (Suprapubic)
		ENSG00000249465.1	RBMXP4	chr4_109437132_C_T_b38	1.7e-9	0.37	Skin - Sun Exposed (Lower leg)
		ENSG00000138802.11	SEC24B	chr4_109437132_C_T_b38	2.9e-9	0.41	Ovary
		ENSG00000247950.6	SEC24B-AS1	chr4_109437132_C_T_b38	8.4e-9	0.31	Skin - Sun Exposed (Lower leg)
		ENSG00000138802.11	SEC24B	chr4_109437132_C_T_b38	1.9e-7	0.15	Esophagus - Mucosa
		ENSG00000138802.11	SEC24B	chr4_109437132_C_T_b38	3.0e-7	0.23	Pancreas
		ENSG00000188517.14	COL25A1	chr4_109437132_C_T_b38	7.3e-7	0.43	Brain - Cortex
		ENSG00000138802.11	SEC24B	chr4_109437132_C_T_b38	0.0000056	0.12	Adipose - Subcutaneous
	rs11933971	ENSG00000225094.3	SETP20	chr4_109437132_C_T_b38	0.000010	0.33	Skin - Sun Exposed (Lower leg)
		ENSG00000138802.11	SEC24B	chr4_109440842_T_C_b38	3.9e-13	0.29	Cells - Cultured fibroblasts
		ENSG00000138802.11	SEC24B	chr4_109440842_T_C_b38	4.8e-11	0.15	Artery - Tibial
		ENSG00000247950.6	SEC24B-AS1	chr4_109440842_T_C_b38	2.5e-10	0.37	Skin - Not Sun Exposed (Suprapubic)
		ENSG00000249465.1	RBMXP4	chr4_109440842_T_C_b38	1.7e-9	0.39	Skin - Not Sun Exposed (Suprapubic)
		ENSG00000138802.11	SEC24B	chr4_109440842_T_C_b38	2.2e-9	0.13	Muscle - Skeletal
		ENSG00000249465.1	RBMXP4	chr4_109440842_T_C_b38	2.5e-9	0.37	Skin - Sun Exposed (Lower leg)
		ENSG00000138802.11	SEC24B	chr4_109440842_T_C_b38	2.9e-9	0.41	Ovary
		ENSG00000247950.6	SEC24B-AS1	chr4_109440842_T_C_b38	3.9e-9	0.32	Skin - Sun Exposed (Lower leg)
		ENSG00000138802.11	SEC24B	chr4_109440842_T_C_b38	1.6e-7	0.15	Esophagus - Mucosa
		ENSG00000138802.11	SEC24B	chr4_109440842_T_C_b38	3.0e-7	0.23	Pancreas
		ENSG00000188517.14	COL25A1	chr4_109440842_T_C_b38	7.3e-7	0.43	Brain - Cortex
		ENSG00000138802.11	SEC24B	chr4_109440842_T_C_b38	0.0000026	0.12	Adipose - Subcutaneous
	rs34836476	ENSG00000225094.3	SETP20	chr4_109440842_T_C_b38	0.000014	0.32	Skin - Sun Exposed (Lower leg)
		ENSG00000138802.11	SEC24B	chr4_109441217_G_C_b38	3.9e-13	0.29	Cells - Cultured fibroblasts
		ENSG00000138802.11	SEC24B	chr4_109441217_G_C_b38	4.8e-11	0.15	Artery - Tibial
		ENSG00000247950.6	SEC24B-AS1	chr4_109441217_G_C_b38	2.5e-10	0.37	Skin - Not Sun Exposed (Suprapubic)
		ENSG00000249465.1	RBMXP4	chr4_109441217_G_C_b38	1.7e-9	0.39	Skin - Not Sun Exposed (Suprapubic)
		ENSG00000138802.11	SEC24B	chr4_109441217_G_C_b38	2.2e-9	0.13	Muscle - Skeletal
		ENSG00000249465.1	RBMXP4	chr4_109441217_G_C_b38	2.5e-9	0.37	Skin - Sun Exposed (Lower leg)
		ENSG00000138802.11	SEC24B	chr4_109441217_G_C_b38	2.9e-9	0.41	Ovary
		ENSG00000247950.6	SEC24B-AS1	chr4_109441217_G_C_b38	3.9e-9	0.32	Skin - Sun Exposed (Lower leg)
SEC24B	rs13103034	ENSG00000138802.11	SEC24B	chr4_109441217_G_C_b38	1.6e-7	0.15	Esophagus - Mucosa

		ENSG00000138802.11	SEC24B	chr4_109441217_G_C_b38	3.0e-7	0.23	Pancreas
		ENSG00000188517.14	COL25A1	chr4_109441217_G_C_b38	7.3e-7	0.43	Brain - Cortex
		ENSG00000138802.11	SEC24B	chr4_109441217_G_C_b38	0.0000026	0.12	Adipose - Subcutaneous
		ENSG00000225094.3	SETP20	chr4_109441217_G_C_b38	0.000014	0.32	Skin - Sun Exposed (Lower leg)
		ENSG00000138802.11	SEC24B	chr4_109441596_T_A_b38	1.9e-15	-0.31	Cells - Cultured fibroblasts
		ENSG00000249465.1	RBMXP4	chr4_109441596_T_A_b38	5.0e-15	-0.50	Skin - Not Sun Exposed (Suprapubic)
		ENSG00000247950.6	SEC24B-AS1	chr4_109441596_T_A_b38	3.8e-13	-0.43	Skin - Not Sun Exposed (Suprapubic)
		ENSG00000249465.1	RBMXP4	chr4_109441596_T_A_b38	5.8e-12	-0.43	Skin - Sun Exposed (Lower leg)
		ENSG00000138802.11	SEC24B	chr4_109441596_T_A_b38	5.8e-11	-0.15	Artery - Tibial
		ENSG00000247950.6	SEC24B-AS1	chr4_109441596_T_A_b38	2.6e-10	-0.35	Skin - Sun Exposed (Lower leg)
		ENSG00000138802.11	SEC24B	chr4_109441596_T_A_b38	7.9e-10	-0.18	Esophagus - Mucosa
		ENSG00000225094.3	SETP20	chr4_109441596_T_A_b38	1.1e-7	-0.39	Skin - Sun Exposed (Lower leg)
		ENSG00000138802.11	SEC24B	chr4_109441596_T_A_b38	3.5e-7	-0.23	Pancreas
		ENSG00000138802.11	SEC24B	chr4_109441596_T_A_b38	0.0000011	-0.35	Ovary
		ENSG00000138802.11	SEC24B	chr4_109441596_T_A_b38	0.0000026	-0.10	Muscle - Skeletal
		ENSG00000005059.15	MCUB	chr4_109441596_T_A_b38	0.0000038	-0.17	Nerve - Tibial
		ENSG00000138802.11	SEC24B	chr4_109441596_T_A_b38	0.0000074	-0.12	Adipose - Subcutaneous
		ENSG00000138802.11	SEC24B	chr4_109441596_T_A_b38	0.000034	-0.32	Brain - Hypothalamus
		ENSG00000188517.14	COL25A1	chr4_109441596_T_A_b38	0.000041	-0.37	Brain - Cortex
	rs6817954	ENSG00000225094.3	SETP20	chr4_109441596_T_A_b38	0.000082	-0.32	Esophagus - Muscularis
		ENSG00000138802.11	SEC24B	chr4_109444270_A_G_b38	4.5e-14	0.30	Cells - Cultured fibroblasts
		ENSG00000138802.11	SEC24B	chr4_109444270_A_G_b38	2.9e-12	0.16	Artery - Tibial
		ENSG00000247950.6	SEC24B-AS1	chr4_109444270_A_G_b38	9.8e-10	0.36	Skin - Not Sun Exposed (Suprapubic)
		ENSG00000138802.11	SEC24B	chr4_109444270_A_G_b38	1.6e-9	0.42	Ovary
		ENSG00000138802.11	SEC24B	chr4_109444270_A_G_b38	2.7e-9	0.17	Esophagus - Mucosa
		ENSG00000138802.11	SEC24B	chr4_109444270_A_G_b38	4.1e-9	0.13	Muscle - Skeletal
		ENSG00000247950.6	SEC24B-AS1	chr4_109444270_A_G_b38	1.9e-8	0.30	Skin - Sun Exposed (Lower leg)
		ENSG00000249465.1	RBMXP4	chr4_109444270_A_G_b38	2.3e-8	0.36	Skin - Not Sun Exposed (Suprapubic)
		ENSG00000249465.1	RBMXP4	chr4_109444270_A_G_b38	6.7e-8	0.33	Skin - Sun Exposed (Lower leg)
		ENSG00000138802.11	SEC24B	chr4_109444270_A_G_b38	6.8e-8	0.14	Adipose - Subcutaneous
		ENSG00000138802.11	SEC24B	chr4_109444270_A_G_b38	1.1e-7	0.23	Pancreas
		ENSG00000225094.3	SETP20	chr4_109444270_A_G_b38	9.3e-7	0.36	Skin - Sun Exposed (Lower leg)
		ENSG00000188517.14	COL25A1	chr4_109444270_A_G_b38	0.0000011	0.41	Brain - Cortex
		ENSG00000005059.15	MCUB	chr4_109444270_A_G_b38	0.000028	0.15	Nerve - Tibial
	rs17040436	ENSG00000225094.3	SETP20	chr4_109444270_A_G_b38	0.000056	0.32	Esophagus - Muscularis
		ENSG00000138802.11	SEC24B	chr4_109444359_A_T_b38	3.9e-13	0.29	Cells - Cultured fibroblasts
		ENSG00000138802.11	SEC24B	chr4_109444359_A_T_b38	4.8e-11	0.15	Artery - Tibial
		ENSG00000247950.6	SEC24B-AS1	chr4_109444359_A_T_b38	2.5e-10	0.37	Skin - Not Sun Exposed (Suprapubic)
		ENSG00000249465.1	RBMXP4	chr4_109444359_A_T_b38	1.7e-9	0.39	Skin - Not Sun Exposed (Suprapubic)
		ENSG00000138802.11	SEC24B	chr4_109444359_A_T_b38	2.2e-9	0.13	Muscle - Skeletal
		ENSG00000249465.1	RBMXP4	chr4_109444359_A_T_b38	2.5e-9	0.37	Skin - Sun Exposed (Lower leg)
		ENSG00000138802.11	SEC24B	chr4_109444359_A_T_b38	2.9e-9	0.41	Ovary
		ENSG00000247950.6	SEC24B-AS1	chr4_109444359_A_T_b38	3.9e-9	0.32	Skin - Sun Exposed (Lower leg)
		ENSG00000138802.11	SEC24B	chr4_109444359_A_T_b38	1.6e-7	0.15	Esophagus - Mucosa
		ENSG00000138802.11	SEC24B	chr4_109444359_A_T_b38	3.0e-7	0.23	Pancreas
		ENSG00000188517.14	COL25A1	chr4_109444359_A_T_b38	7.3e-7	0.43	Brain - Cortex
		ENSG00000138802.11	SEC24B	chr4_109444359_A_T_b38	0.0000026	0.12	Adipose - Subcutaneous
	rs17040439	ENSG00000225094.3	SETP20	chr4_109444359_A_T_b38	0.000014	0.32	Skin - Sun Exposed (Lower leg)
	rs10009463	ENSG00000249465.1	RBMXP4	chr4_109452526_A_G_b38	0.000031	0.59	Skin - Not Sun Exposed (Suprapubic)
	rs28597954	ENSG00000249465.1	RBMXP4	chr4_109455006_C_T_b38	0.000031	0.59	Skin - Not Sun Exposed (Suprapubic)
	rs28663368	ENSG00000249465.1	RBMXP4	chr4_109455240_A_G_b38	0.000031	0.59	Skin - Not Sun Exposed (Suprapubic)
		ENSG00000249465.1	RBMXP4	chr4_109457196_G_A_b38	1.9e-23	0.71	Skin - Sun Exposed (Lower leg)
		ENSG00000249465.1	RBMXP4	chr4_109457196_G_A_b38	3.5e-20	0.68	Skin - Not Sun Exposed (Suprapubic)
		ENSG00000247950.6	SEC24B-AS1	chr4_109457196_G_A_b38	6.7e-11	0.41	Skin - Sun Exposed (Lower leg)
		ENSG00000138802.11	SEC24B	chr4_109457196_G_A_b38	1.4e-10	0.30	Cells - Cultured fibroblasts
		ENSG00000247950.6	SEC24B-AS1	chr4_109457196_G_A_b38	2.5e-10	0.44	Skin - Not Sun Exposed (Suprapubic)
		ENSG00000138802.11	SEC24B	chr4_109457196_G_A_b38	4.2e-9	0.16	Artery - Tibial
		ENSG00000138802.11	SEC24B	chr4_109457196_G_A_b38	3.1e-8	0.46	Ovary
		ENSG00000138802.11	SEC24B	chr4_109457196_G_A_b38	3.0e-7	0.26	Pancreas
		ENSG00000138802.11	SEC24B	chr4_109457196_G_A_b38	0.0000041	0.14	Adipose - Subcutaneous
		ENSG00000138802.11	SEC24B	chr4_109457196_G_A_b38	0.000016	0.11	Muscle - Skeletal
		ENSG00000249965.2	CDC42P4	chr4_109457196_G_A_b38	0.000088	0.40	Adipose - Visceral (Omentum)
	rs34132975	ENSG00000247950.6	SEC24B-AS1	chr4_109457196_G_A_b38	0.000099	-0.24	Adipose - Subcutaneous
		ENSG00000249465.1	RBMXP4	chr4_109458110_A_G_b38	1.6e-11	-1.1	Testis
		ENSG00000249465.1	RBMXP4	chr4_109458110_A_G_b38	9.0e-10	-0.68	Nerve - Tibial
		ENSG00000249465.1	RBMXP4	chr4_109458110_A_G_b38	4.3e-7	-1.1	Brain - Cortex
		ENSG00000249465.1	RBMXP4	chr4_109458110_A_G_b38	0.0000013	-0.47	Adipose - Subcutaneous
	rs77911134	ENSG00000138802.11	SEC24B	chr4_109458110_A_G_b38	0.0000017	0.24	Esophagus - Mucosa
		ENSG00000249465.1	RBMXP4	chr4_109458514_G_T_b38	7.2e-12	-1.1	Testis
		ENSG00000249465.1	RBMXP4	chr4_109458514_G_T_b38	7.8e-10	-0.68	Nerve - Tibial
		ENSG00000249465.1	RBMXP4	chr4_109458514_G_T_b38	5.3e-7	-1.1	Brain - Cortex
		ENSG00000249465.1	RBMXP4	chr4_109458514_G_T_b38	0.0000018	-0.46	Adipose - Subcutaneous
		ENSG00000138802.11	SEC24B	chr4_109458514_G_T_b38	0.0000019	0.23	Esophagus - Mucosa
		ENSG00000249465.1	RBMXP4	chr4_109458514_G_T_b38	0.000016	-0.70	Heart - Atrial Appendage
	rs76784285	ENSG00000249465.1	RBMXP4	chr4_109458514_G_T_b38	0.000020	-0.95	Brain - Frontal Cortex (BA9)
	rs7697001	ENSG00000249465.1	RBMXP4	chr4_109460796_G_A_b38	0.000029	0.61	Skin - Not Sun Exposed (Suprapubic)
	rs28680423	ENSG00000249465.1	RBMXP4	chr4_109464031_C_G_b38	0.000031	0.59	Skin - Not Sun Exposed (Suprapubic)
	rs6840561	ENSG00000249465.1	RBMXP4	chr4_109464445_A_G_b38	0.000031	0.59	Skin - Not Sun Exposed (Suprapubic)
		ENSG00000249465.1	RBMXP4	chr4_109464602_T_A_b38	7.2e-12	-1.1	Testis
		ENSG00000249465.1	RBMXP4	chr4_109464602_T_A_b38	7.8e-10	-0.68	Nerve - Tibial
		ENSG00000249465.1	RBMXP4	chr4_109464602_T_A_b38	5.3e-7	-1.1	Brain - Cortex
	rs17040454	ENSG00000249465.1	RBMXP4	chr4_109464602_T_A_b38	0.0000018	-0.46	Adipose - Subcutaneous

		ENSG00000138802.11	SEC24B	chr4_109464602_T_A_b38	0.0000019	0.23	Esophagus - Mucosa
		ENSG00000249465.1	RBMXP4	chr4_109464602_T_A_b38	0.000016	-0.70	Heart - Atrial Appendage
		ENSG00000249465.1	RBMXP4	chr4_109464602_T_A_b38	0.000020	-0.95	Brain - Frontal Cortex (BA9)
		ENSG00000249465.1	RBMXP4	chr4_109465287_T_A_b38	7.2e-12	-1.1	Testis
		ENSG00000249465.1	RBMXP4	chr4_109465287_T_A_b38	7.8e-10	-0.68	Nerve - Tibial
		ENSG00000249465.1	RBMXP4	chr4_109465287_T_A_b38	5.3e-7	-1.1	Brain - Cortex
		ENSG00000249465.1	RBMXP4	chr4_109465287_T_A_b38	0.0000018	-0.46	Adipose - Subcutaneous
		ENSG00000138802.11	SEC24B	chr4_109465287_T_A_b38	0.0000019	0.23	Esophagus - Mucosa
		ENSG00000249465.1	RBMXP4	chr4_109465287_T_A_b38	0.000016	-0.70	Heart - Atrial Appendage
	rs12643881	ENSG00000249465.1	RBMXP4	chr4_109465287_T_A_b38	0.000020	-0.95	Brain - Frontal Cortex (BA9)
		ENSG00000249465.1	RBMXP4	chr4_109466601_A_G_b38	1.0e-23	0.72	Skin - Sun Exposed (Lower leg)
		ENSG00000249465.1	RBMXP4	chr4_109466601_A_G_b38	1.5e-21	0.71	Skin - Not Sun Exposed (Suprapubic)
		ENSG00000247950.6	SEC24B-AS1	chr4_109466601_A_G_b38	6.2e-12	0.44	Skin - Sun Exposed (Lower leg)
		ENSG00000138802.11	SEC24B	chr4_109466601_A_G_b38	8.5e-10	0.29	Cells - Cultured fibroblasts
		ENSG00000247950.6	SEC24B-AS1	chr4_109466601_A_G_b38	2.7e-9	0.42	Skin - Not Sun Exposed (Suprapubic)
		ENSG00000138802.11	SEC24B	chr4_109466601_A_G_b38	7.5e-8	0.45	Ovary
		ENSG00000138802.11	SEC24B	chr4_109466601_A_G_b38	0.0000020	0.13	Artery - Tibial
		ENSG00000138802.11	SEC24B	chr4_109466601_A_G_b38	0.000020	0.22	Pancreas
		ENSG00000138802.11	SEC24B	chr4_109466601_A_G_b38	0.000032	0.13	Adipose - Subcutaneous
	rs77573441	ENSG00000225094.3	SETP20	chr4_109466601_A_G_b38	0.000090	0.34	Skin - Sun Exposed (Lower leg)
	rs10706780	ENSG00000249465.1	RBMXP4	chr4_109468127_AC_A_b38	0.000013	0.61	Skin - Not Sun Exposed (Suprapubic)
		ENSG00000249465.1	RBMXP4	chr4_109468424_C_A_b38	1.9e-23	0.71	Skin - Sun Exposed (Lower leg)
		ENSG00000249465.1	RBMXP4	chr4_109468424_C_A_b38	3.5e-20	0.68	Skin - Not Sun Exposed (Suprapubic)
		ENSG00000247950.6	SEC24B-AS1	chr4_109468424_C_A_b38	6.7e-11	0.41	Skin - Sun Exposed (Lower leg)
		ENSG00000138802.11	SEC24B	chr4_109468424_C_A_b38	1.4e-10	0.30	Cells - Cultured fibroblasts
		ENSG00000247950.6	SEC24B-AS1	chr4_109468424_C_A_b38	2.5e-10	0.44	Skin - Not Sun Exposed (Suprapubic)
		ENSG00000138802.11	SEC24B	chr4_109468424_C_A_b38	4.2e-9	0.16	Artery - Tibial
		ENSG00000138802.11	SEC24B	chr4_109468424_C_A_b38	3.1e-8	0.46	Ovary
		ENSG00000138802.11	SEC24B	chr4_109468424_C_A_b38	3.0e-7	0.26	Pancreas
		ENSG00000138802.11	SEC24B	chr4_109468424_C_A_b38	0.0000041	0.14	Adipose - Subcutaneous
		ENSG00000138802.11	SEC24B	chr4_109468424_C_A_b38	0.000016	0.11	Muscle - Skeletal
		ENSG00000249965.2	CDC42P4	chr4_109468424_C_A_b38	0.000088	0.40	Adipose - Visceral (Omentum)
	rs34813537	ENSG00000247950.6	SEC24B-AS1	chr4_109468424_C_A_b38	0.000099	-0.24	Adipose - Subcutaneous
	rs7681265	ENSG00000249465.1	RBMXP4	chr4_109470806_C_A_b38	0.000051	0.60	Skin - Not Sun Exposed (Suprapubic)
	rs10606064	ENSG00000249465.1	RBMXP4	chr4_109471637_CAT_C_b38	0.000013	0.61	Skin - Not Sun Exposed (Suprapubic)
	rs17040466	ENSG00000249465.1	RBMXP4	chr4_109472494_A_G_b38	0.000013	0.61	Skin - Not Sun Exposed (Suprapubic)
		ENSG00000249465.1	RBMXP4	chr4_109472593_TACTC_T_b38	1.1e-20	0.65	Skin - Sun Exposed (Lower leg)
		ENSG00000249465.1	RBMXP4	chr4_109472593_TACTC_T_b38	6.9e-19	0.65	Skin - Not Sun Exposed (Suprapubic)
		ENSG00000247950.6	SEC24B-AS1	chr4_109472593_TACTC_T_b38	2.9e-11	0.42	Skin - Sun Exposed (Lower leg)
		ENSG00000138802.11	SEC24B	chr4_109472593_TACTC_T_b38	3.2e-10	0.29	Cells - Cultured fibroblasts
		ENSG00000247950.6	SEC24B-AS1	chr4_109472593_TACTC_T_b38	1.8e-9	0.41	Skin - Not Sun Exposed (Suprapubic)
		ENSG00000138802.11	SEC24B	chr4_109472593_TACTC_T_b38	6.1e-9	0.16	Artery - Tibial
		ENSG00000138802.11	SEC24B	chr4_109472593_TACTC_T_b38	3.1e-8	0.46	Ovary
		ENSG00000138802.11	SEC24B	chr4_109472593_TACTC_T_b38	0.0000051	0.22	Pancreas
		ENSG00000138802.11	SEC24B	chr4_109472593_TACTC_T_b38	0.0000092	0.13	Adipose - Subcutaneous
		ENSG00000138802.11	SEC24B	chr4_109472593_TACTC_T_b38	0.000024	0.11	Muscle - Skeletal
	rs67518796	ENSG00000249965.2	CDC42P4	chr4_109472593_TACTC_T_b38	0.000065	0.40	Adipose - Visceral (Omentum)
		ENSG00000123739.10	PLA2G12A	chr4_109473990_T_C_b38	6.3e-8	-0.19	Nerve - Tibial
		ENSG00000123739.10	PLA2G12A	chr4_109473990_T_C_b38	3.1e-7	-0.21	Esophagus - Mucosa
		ENSG00000138794.9	CASP6	chr4_109473990_T_C_b38	3.9e-7	-0.25	Heart - Atrial Appendage
		ENSG00000123739.10	PLA2G12A	chr4_109473990_T_C_b38	0.0000019	-0.30	Brain - Putamen (basal ganglia)
		ENSG00000123739.10	PLA2G12A	chr4_109473990_T_C_b38	0.0000045	-0.10	Cells - Cultured fibroblasts
		ENSG00000138802.11	SEC24B	chr4_109473990_T_C_b38	0.000011	-0.13	Cells - Cultured fibroblasts
		ENSG00000138802.11	SEC24B	chr4_109473990_T_C_b38	0.000043	-0.13	Artery - Aorta
	rs10516556	ENSG00000123739.10	PLA2G12A	chr4_109473990_T_C_b38	0.000068	-0.14	Thyroid
		ENSG00000249465.1	RBMXP4	chr4_109474250_G_A_b38	7.2e-12	-1.1	Testis
		ENSG00000249465.1	RBMXP4	chr4_109474250_G_A_b38	7.8e-10	-0.68	Nerve - Tibial
		ENSG00000249465.1	RBMXP4	chr4_109474250_G_A_b38	5.3e-7	-1.1	Brain - Cortex
		ENSG00000249465.1	RBMXP4	chr4_109474250_G_A_b38	0.0000018	-0.46	Adipose - Subcutaneous
		ENSG00000138802.11	SEC24B	chr4_109474250_G_A_b38	0.0000019	0.23	Esophagus - Mucosa
		ENSG00000249465.1	RBMXP4	chr4_109474250_G_A_b38	0.000016	-0.70	Heart - Atrial Appendage
	rs10516557	ENSG00000249465.1	RBMXP4	chr4_109474250_G_A_b38	0.000020	-0.95	Brain - Frontal Cortex (BA9)
		ENSG00000249465.1	RBMXP4	chr4_109476568_T_A_b38	6.3e-23	0.70	Skin - Sun Exposed (Lower leg)
		ENSG00000249465.1	RBMXP4	chr4_109476568_T_A_b38	6.0e-20	0.68	Skin - Not Sun Exposed (Suprapubic)
		ENSG00000247950.6	SEC24B-AS1	chr4_109476568_T_A_b38	1.3e-11	0.43	Skin - Sun Exposed (Lower leg)
		ENSG00000138802.11	SEC24B	chr4_109476568_T_A_b38	1.7e-10	0.30	Cells - Cultured fibroblasts
		ENSG00000247950.6	SEC24B-AS1	chr4_109476568_T_A_b38	1.8e-10	0.44	Skin - Not Sun Exposed (Suprapubic)
		ENSG00000138802.11	SEC24B	chr4_109476568_T_A_b38	1.4e-8	0.16	Artery - Tibial
		ENSG00000138802.11	SEC24B	chr4_109476568_T_A_b38	3.1e-8	0.46	Ovary
		ENSG00000138802.11	SEC24B	chr4_109476568_T_A_b38	0.0000032	0.24	Pancreas
		ENSG00000138802.11	SEC24B	chr4_109476568_T_A_b38	0.0000057	0.14	Adipose - Subcutaneous
		ENSG00000138802.11	SEC24B	chr4_109476568_T_A_b38	0.000019	0.11	Muscle - Skeletal
	rs34320670	ENSG00000249965.2	CDC42P4	chr4_109476568_T_A_b38	0.000088	0.40	Adipose - Visceral (Omentum)
	rs28580356	ENSG00000249465.1	RBMXP4	chr4_109477131_C_T_b38	0.000021	0.61	Skin - Not Sun Exposed (Suprapubic)
	**rs28541279**	ENSG00000249465.1	RBMXP4	chr4_109477734_T_G_b38	0.000031	0.59	Skin - Not Sun Exposed (Suprapubic)
		ENSG00000249465.1	RBMXP4	chr4_109478075_A_G_b38	6.3e-23	0.70	Skin - Sun Exposed (Lower leg)
		ENSG00000249465.1	RBMXP4	chr4_109478075_A_G_b38	6.0e-20	0.68	Skin - Not Sun Exposed (Suprapubic)
		ENSG00000247950.6	SEC24B-AS1	chr4_109478075_A_G_b38	1.3e-11	0.43	Skin - Sun Exposed (Lower leg)
	**rs35866656**	ENSG00000138802.11	SEC24B	chr4_109478075_A_G_b38	1.7e-10	0.30	Cells - Cultured fibroblasts
		ENSG00000247950.6	SEC24B-AS1	chr4_109478075_A_G_b38	1.8e-10	0.44	Skin - Not Sun Exposed (Suprapubic)
		ENSG00000138802.11	SEC24B	chr4_109478075_A_G_b38	1.4e-8	0.16	Artery - Tibial
		ENSG00000138802.11	SEC24B	chr4_109478075_A_G_b38	3.1e-8	0.46	Ovary

		ENSG00000138802.11	SEC24B	chr4_109478075_A_G_b38	0.0000032	0.24	Pancreas
		ENSG00000138802.11	SEC24B	chr4_109478075_A_G_b38	0.0000057	0.14	Adipose - Subcutaneous
		ENSG00000138802.11	SEC24B	chr4_109478075_A_G_b38	0.000019	0.11	Muscle - Skeletal
		ENSG00000249965.2	CDC42P4	chr4_109478075_A_G_b38	0.000088	0.40	Adipose - Visceral (Omentum)
	**rs28460762**	ENSG00000249465.1	RBMXP4	chr4_109478457_A_C_b38	0.000031	0.59	Skin - Not Sun Exposed (Suprapubic)
	**SNP Id**	**Gencode Id**	**eGene**	**Variant Id**	**P-Value**	**NES**	**Tissue**
		ENSG00000253404.1	AC034243.1	chr5_138782249_A_G_b38	1.3e-147	-1.2	Thyroid
		ENSG00000253404.1	AC034243.1	chr5_138782249_A_G_b38	3.2e-108	-1.1	Skin - Sun Exposed (Lower leg)
		ENSG00000253404.1	AC034243.1	chr5_138782249_A_G_b38	1.3e-100	-1.1	Adipose - Subcutaneous
		ENSG00000253404.1	AC034243.1	chr5_138782249_A_G_b38	1.0e-89	-1.1	Skin - Not Sun Exposed (Suprapubic)
		ENSG00000253404.1	AC034243.1	chr5_138782249_A_G_b38	2.5e-83	-1.1	Esophagus - Mucosa
		ENSG00000253404.1	AC034243.1	chr5_138782249_A_G_b38	1.7e-80	-0.98	Nerve - Tibial
		ENSG00000253404.1	AC034243.1	chr5_138782249_A_G_b38	8.2e-77	-0.98	Lung
		ENSG00000044115.20	CTNNA1	chr5_138782249_A_G_b38	4.0e-76	0.49	Whole Blood
		ENSG00000253404.1	AC034243.1	chr5_138782249_A_G_b38	4.7e-73	-1.1	Breast - Mammary Tissue
		ENSG00000253404.1	AC034243.1	chr5_138782249_A_G_b38	3.5e-68	-1.1	Heart - Atrial Appendage
		ENSG00000253404.1	AC034243.1	chr5_138782249_A_G_b38	9.4e-68	-0.97	Adipose - Visceral (Omentum)
		ENSG00000253404.1	AC034243.1	chr5_138782249_A_G_b38	1.5e-65	-0.97	Esophagus - Muscularis
		ENSG00000253404.1	AC034243.1	chr5_138782249_A_G_b38	1.3e-64	-1.2	Pancreas
		ENSG00000253404.1	AC034243.1	chr5_138782249_A_G_b38	2.8e-61	-1.2	Pituitary
		ENSG00000253404.1	AC034243.1	chr5_138782249_A_G_b38	3.4e-60	-0.97	Stomach
		ENSG00000253404.1	AC034243.1	chr5_138782249_A_G_b38	8.8e-55	-0.98	Heart - Left Ventricle
		ENSG00000253404.1	AC034243.1	chr5_138782249_A_G_b38	7.4e-54	-0.82	Cells - Cultured fibroblasts
		ENSG00000253404.1	AC034243.1	chr5_138782249_A_G_b38	1.8e-53	-1.2	Adrenal Gland
		ENSG00000253404.1	AC034243.1	chr5_138782249_A_G_b38	1.1e-52	-0.98	Colon - Transverse
		ENSG00000253404.1	AC034243.1	chr5_138782249_A_G_b38	1.9e-48	-0.98	Prostate
		ENSG00000044115.20	CTNNA1	chr5_138782249_A_G_b38	1.5e-43	0.27	Esophagus - Mucosa
		ENSG00000253404.1	AC034243.1	chr5_138782249_A_G_b38	9.5e-38	-0.94	Colon - Sigmoid
		ENSG00000253404.1	AC034243.1	chr5_138782249_A_G_b38	6.9e-37	-0.90	Esophagus - Gastroesophageal Junction
		ENSG00000253404.1	AC034243.1	chr5_138782249_A_G_b38	4.2e-35	-1.1	Spleen
		ENSG00000253404.1	AC034243.1	chr5_138782249_A_G_b38	1.1e-27	-1.2	Minor Salivary Gland
		ENSG00000253404.1	AC034243.1	chr5_138782249_A_G_b38	1.4e-27	-0.94	Liver
		ENSG00000253404.1	AC034243.1	chr5_138782249_A_G_b38	2.1e-26	-1.1	Brain - Nucleus accumbens (basal ganglia)
		ENSG00000253404.1	AC034243.1	chr5_138782249_A_G_b38	2.8e-26	-1.1	Ovary
		ENSG00000253404.1	AC034243.1	chr5_138782249_A_G_b38	7.7e-26	-0.98	Brain - Putamen (basal ganglia)
		ENSG00000253404.1	AC034243.1	chr5_138782249_A_G_b38	1.1e-25	-1.0	Brain - Caudate (basal ganglia)
		ENSG00000253404.1	AC034243.1	chr5_138782249_A_G_b38	1.6e-25	-0.50	Testis
		ENSG00000253404.1	AC034243.1	chr5_138782249_A_G_b38	1.0e-24	-0.91	Artery - Coronary
		ENSG00000253404.1	AC034243.1	chr5_138782249_A_G_b38	1.9e-22	-0.87	Brain - Cortex
		ENSG00000253404.1	AC034243.1	chr5_138782249_A_G_b38	1.6e-20	-1.1	Brain - Spinal cord (cervical c-1)
		ENSG00000253404.1	AC034243.1	chr5_138782249_A_G_b38	4.9e-20	-0.86	Small Intestine - Terminal Ileum
		ENSG00000253404.1	AC034243.1	chr5_138782249_A_G_b38	4.2e-19	-1.1	Uterus
		ENSG00000120725.12	SIL1	chr5_138782249_A_G_b38	9.6e-19	-0.34	Esophagus - Muscularis
	rs700626	ENSG00000253404.1	AC034243.1	chr5_138782249_A_G_b38	1.1e-18	-0.91	Brain - Hypothalamus
		ENSG00000253404.1	AC034243.1	chr5_138782249_A_G_b38	5.8e-18	-1.1	Brain - Amygdala
		ENSG00000253404.1	AC034243.1	chr5_138782249_A_G_b38	2.4e-17	-1.1	Vagina
		ENSG00000120725.12	SIL1	chr5_138782249_A_G_b38	3.4e-17	-0.22	Skin - Sun Exposed (Lower leg)
		ENSG00000253404.1	AC034243.1	chr5_138782249_A_G_b38	5.2e-17	-0.91	Brain - Hippocampus
		ENSG00000253404.1	AC034243.1	chr5_138782249_A_G_b38	9.7e-17	-0.97	Brain - Substantia nigra
		ENSG00000120725.12	SIL1	chr5_138782249_A_G_b38	1.0e-16	-0.20	Nerve - Tibial
		ENSG00000120725.12	SIL1	chr5_138782249_A_G_b38	1.6e-15	-0.28	Artery - Aorta
		ENSG00000253404.1	AC034243.1	chr5_138782249_A_G_b38	2.2e-15	-0.75	Brain - Frontal Cortex (BA9)
		ENSG00000120725.12	SIL1	chr5_138782249_A_G_b38	8.5e-15	-0.30	Stomach
		ENSG00000253404.1	AC034243.1	chr5_138782249_A_G_b38	1.9e-14	-0.75	Brain - Anterior cingulate cortex (BA24)
		ENSG00000120725.12	SIL1	chr5_138782249_A_G_b38	4.6e-14	-0.21	Skin - Not Sun Exposed (Suprapubic)
		ENSG00000120725.12	SIL1	chr5_138782249_A_G_b38	2.0e-10	-0.13	Thyroid
		ENSG00000253404.1	AC034243.1	chr5_138782249_A_G_b38	3.6e-10	-1.0	Kidney - Cortex
		ENSG00000044115.20	CTNNA1	chr5_138782249_A_G_b38	4.0e-10	0.14	Cells - Cultured fibroblasts
		ENSG00000044115.20	CTNNA1	chr5_138782249_A_G_b38	5.0e-10	0.12	Nerve - Tibial
		ENSG00000120725.12	SIL1	chr5_138782249_A_G_b38	6.9e-10	-0.15	Artery - Tibial
		ENSG00000120725.12	SIL1	chr5_138782249_A_G_b38	1.3e-9	-0.14	Lung
		ENSG00000120725.12	SIL1	chr5_138782249_A_G_b38	3.2e-9	-0.15	Adipose - Visceral (Omentum)
		ENSG00000120725.12	SIL1	chr5_138782249_A_G_b38	8.8e-9	-0.15	Adipose - Subcutaneous
		ENSG00000120725.12	SIL1	chr5_138782249_A_G_b38	3.6e-8	-0.17	Esophagus - Mucosa
		ENSG00000120725.12	SIL1	chr5_138782249_A_G_b38	5.0e-8	-0.27	Esophagus - Gastroesophageal Junction
		ENSG00000120725.12	SIL1	chr5_138782249_A_G_b38	5.4e-8	-0.12	Cells - Cultured fibroblasts
		ENSG00000120725.12	SIL1	chr5_138782249_A_G_b38	6.5e-8	-0.41	Brain - Frontal Cortex (BA9)
		ENSG00000120725.12	SIL1	chr5_138782249_A_G_b38	1.1e-7	-0.26	Pituitary
		ENSG00000044115.20	CTNNA1	chr5_138782249_A_G_b38	1.3e-7	0.14	Breast - Mammary Tissue
		ENSG00000120725.12	SIL1	chr5_138782249_A_G_b38	1.4e-7	-0.24	Adrenal Gland
		ENSG00000044115.20	CTNNA1	chr5_138782249_A_G_b38	2.3e-7	0.15	Colon - Transverse
		ENSG00000120725.12	SIL1	chr5_138782249_A_G_b38	2.7e-7	-0.33	Brain - Caudate (basal ganglia)
		ENSG00000044115.20	CTNNA1	chr5_138782249_A_G_b38	3.2e-7	0.095	Thyroid
		ENSG00000044115.20	CTNNA1	chr5_138782249_A_G_b38	4.2e-7	0.098	Skin - Not Sun Exposed (Suprapubic)
		ENSG00000044115.20	CTNNA1	chr5_138782249_A_G_b38	7.0e-7	-0.098	Testis
		ENSG00000044115.20	CTNNA1	chr5_138782249_A_G_b38	9.3e-7	0.15	Spleen
		ENSG00000120725.12	SIL1	chr5_138782249_A_G_b38	0.0000010	-0.30	Brain - Cortex
		ENSG00000044115.20	CTNNA1	chr5_138782249_A_G_b38	0.0000016	0.14	Muscle - Skeletal
		ENSG00000120725.12	SIL1	chr5_138782249_A_G_b38	0.0000023	-0.14	Breast - Mammary Tissue
		ENSG00000120725.12	SIL1	chr5_138782249_A_G_b38	0.0000035	-0.41	Brain - Substantia nigra
		ENSG00000120725.12	SIL1	chr5_138782249_A_G_b38	0.0000048	-0.26	Brain - Cerebellum
**CTNNA1**		ENSG00000120725.12	SIL1	chr5_138782249_A_G_b38	0.0000049	-0.33	Brain - Nucleus accumbens (basal ganglia)

		ENSG00000120725.12	SIL1	chr5_138782249_A_G_b38	0.0000064	-0.28	Artery - Coronary
		ENSG00000044115.20	CTNNA1	chr5_138782249_A_G_b38	0.0000088	0.080	Skin - Sun Exposed (Lower leg)
		ENSG00000120725.12	SIL1	chr5_138782249_A_G_b38	0.000011	-0.13	Heart - Left Ventricle
		ENSG00000120725.12	SIL1	chr5_138782249_A_G_b38	0.000012	-0.17	Colon - Transverse
		ENSG00000044115.20	CTNNA1	chr5_138782249_A_G_b38	0.000014	0.11	Heart - Atrial Appendage
		ENSG00000120725.12	SIL1	chr5_138782249_A_G_b38	0.000020	-0.15	Pancreas
		ENSG00000120725.12	SIL1	chr5_138782249_A_G_b38	0.000029	-0.20	Colon - Sigmoid
		ENSG00000120725.12	SIL1	chr5_138782249_A_G_b38	0.000056	-0.31	Brain - Anterior cingulate cortex (BA24)
		ENSG00000044115.20	CTNNA1	chr5_138782249_A_G_b38	0.000077	0.19	Brain - Cerebellar Hemisphere
		ENSG00000044115.20	CTNNA1	chr5_138782249_A_G_b38	0.000095	0.098	Adipose - Subcutaneous
	rs13173618	ENSG00000253404.1	AC034243.1	chr5_138804091_T_G_b38	7.3e-108	1.1	Thyroid
		ENSG00000253404.1	AC034243.1	chr5_138804091_T_G_b38	4.6e-81	1.1	Skin - Sun Exposed (Lower leg)
		ENSG00000253404.1	AC034243.1	chr5_138804091_T_G_b38	2.3e-69	0.97	Adipose - Subcutaneous
		ENSG00000044115.20	CTNNA1	chr5_138804091_T_G_b38	2.2e-63	-0.47	Whole Blood
		ENSG00000253404.1	AC034243.1	chr5_138804091_T_G_b38	1.3e-60	0.91	Nerve - Tibial
		ENSG00000253404.1	AC034243.1	chr5_138804091_T_G_b38	3.2e-60	1.0	Skin - Not Sun Exposed (Suprapubic)
		ENSG00000253404.1	AC034243.1	chr5_138804091_T_G_b38	6.3e-58	0.99	Esophagus - Mucosa
		ENSG00000253404.1	AC034243.1	chr5_138804091_T_G_b38	3.6e-55	0.87	Lung
		ENSG00000253404.1	AC034243.1	chr5_138804091_T_G_b38	7.4e-55	1.0	Breast - Mammary Tissue
		ENSG00000253404.1	AC034243.1	chr5_138804091_T_G_b38	3.8e-52	1.2	Pituitary
		ENSG00000253404.1	AC034243.1	chr5_138804091_T_G_b38	5.6e-52	0.91	Esophagus - Muscularis
		ENSG00000253404.1	AC034243.1	chr5_138804091_T_G_b38	2.3e-50	0.90	Adipose - Visceral (Omentum)
		ENSG00000253404.1	AC034243.1	chr5_138804091_T_G_b38	2.3e-48	1.0	Heart - Atrial Appendage
		ENSG00000253404.1	AC034243.1	chr5_138804091_T_G_b38	1.8e-44	1.1	Pancreas
		ENSG00000253404.1	AC034243.1	chr5_138804091_T_G_b38	6.8e-44	0.92	Heart - Left Ventricle
		ENSG00000253404.1	AC034243.1	chr5_138804091_T_G_b38	2.9e-43	0.90	Stomach
		ENSG00000253404.1	AC034243.1	chr5_138804091_T_G_b38	4.5e-40	0.74	Cells - Cultured fibroblasts
		ENSG00000253404.1	AC034243.1	chr5_138804091_T_G_b38	3.7e-37	1.1	Adrenal Gland
		ENSG00000253404.1	AC034243.1	chr5_138804091_T_G_b38	4.3e-34	0.85	Colon - Transverse
		ENSG00000044115.20	CTNNA1	chr5_138804091_T_G_b38	4.0e-33	-0.25	Esophagus - Mucosa
		ENSG00000253404.1	AC034243.1	chr5_138804091_T_G_b38	2.2e-32	0.88	Prostate
		ENSG00000253404.1	AC034243.1	chr5_138804091_T_G_b38	2.9e-25	1.0	Brain - Putamen (basal ganglia)
		ENSG00000253404.1	AC034243.1	chr5_138804091_T_G_b38	6.8e-25	0.79	Esophagus - Gastroesophageal Junction
		ENSG00000253404.1	AC034243.1	chr5_138804091_T_G_b38	4.1e-24	1.1	Brain - Nucleus accumbens (basal ganglia)
		ENSG00000253404.1	AC034243.1	chr5_138804091_T_G_b38	2.1e-23	1.0	Brain - Caudate (basal ganglia)
		ENSG00000253404.1	AC034243.1	chr5_138804091_T_G_b38	2.8e-23	0.81	Colon - Sigmoid
		ENSG00000253404.1	AC034243.1	chr5_138804091_T_G_b38	1.6e-21	0.90	Spleen
		ENSG00000253404.1	AC034243.1	chr5_138804091_T_G_b38	4.9e-21	0.87	Brain - Cortex
		ENSG00000253404.1	AC034243.1	chr5_138804091_T_G_b38	2.4e-20	0.87	Liver
		ENSG00000253404.1	AC034243.1	chr5_138804091_T_G_b38	7.2e-19	1.0	Brain - Spinal cord (cervical c-1)
		ENSG00000253404.1	AC034243.1	chr5_138804091_T_G_b38	1.6e-18	0.45	Testis
		ENSG00000253404.1	AC034243.1	chr5_138804091_T_G_b38	1.9e-18	0.83	Artery - Coronary
		ENSG00000253404.1	AC034243.1	chr5_138804091_T_G_b38	1.4e-17	0.98	Ovary
		ENSG00000253404.1	AC034243.1	chr5_138804091_T_G_b38	2.0e-17	0.97	Brain - Substantia nigra
		ENSG00000253404.1	AC034243.1	chr5_138804091_T_G_b38	2.4e-17	0.93	Brain - Hippocampus
		ENSG00000120725.12	SIL1	chr5_138804091_T_G_b38	5.7e-17	0.22	Skin - Sun Exposed (Lower leg)
		ENSG00000253404.1	AC034243.1	chr5_138804091_T_G_b38	1.1e-16	1.2	Brain - Amygdala
		ENSG00000253404.1	AC034243.1	chr5_138804091_T_G_b38	1.5e-16	0.81	Brain - Frontal Cortex (BA9)
		ENSG00000253404.1	AC034243.1	chr5_138804091_T_G_b38	1.7e-16	0.89	Brain - Hypothalamus
		ENSG00000253404.1	AC034243.1	chr5_138804091_T_G_b38	1.9e-16	1.0	Minor Salivary Gland
		ENSG00000120725.12	SIL1	chr5_138804091_T_G_b38	1.2e-15	0.23	Skin - Not Sun Exposed (Suprapubic)
		ENSG00000253404.1	AC034243.1	chr5_138804091_T_G_b38	1.5e-14	1.0	Uterus
		ENSG00000253404.1	AC034243.1	chr5_138804091_T_G_b38	2.3e-14	0.78	Brain - Anterior cingulate cortex (BA24)
		ENSG00000120725.12	SIL1	chr5_138804091_T_G_b38	4.4e-14	0.19	Nerve - Tibial
		ENSG00000120725.12	SIL1	chr5_138804091_T_G_b38	7.4e-14	0.28	Artery - Aorta
		ENSG00000120725.12	SIL1	chr5_138804091_T_G_b38	2.1e-13	0.29	Esophagus - Muscularis
		ENSG00000253404.1	AC034243.1	chr5_138804091_T_G_b38	3.4e-13	0.72	Small Intestine - Terminal Ileum
		ENSG00000253404.1	AC034243.1	chr5_138804091_T_G_b38	3.4e-12	0.94	Vagina
		ENSG00000120725.12	SIL1	chr5_138804091_T_G_b38	3.6e-11	0.27	Stomach
		ENSG00000120725.12	SIL1	chr5_138804091_T_G_b38	1.0e-10	0.14	Thyroid
		ENSG00000120725.12	SIL1	chr5_138804091_T_G_b38	1.8e-10	0.15	Lung
		ENSG00000253404.1	AC034243.1	chr5_138804091_T_G_b38	2.0e-10	1.0	Kidney - Cortex
		ENSG00000120725.12	SIL1	chr5_138804091_T_G_b38	3.3e-9	0.15	Artery - Tibial
		ENSG00000044115.20	CTNNA1	chr5_138804091_T_G_b38	3.3e-9	-0.14	Cells - Cultured fibroblasts
		ENSG00000044115.20	CTNNA1	chr5_138804091_T_G_b38	4.9e-9	-0.11	Nerve - Tibial
		ENSG00000120725.12	SIL1	chr5_138804091_T_G_b38	7.9e-8	0.14	Adipose - Visceral (Omentum)
		ENSG00000044115.20	CTNNA1	chr5_138804091_T_G_b38	1.1e-7	-0.14	Breast - Mammary Tissue
		ENSG00000120725.12	SIL1	chr5_138804091_T_G_b38	1.3e-7	0.27	Esophagus - Gastroesophageal Junction
		ENSG00000120725.12	SIL1	chr5_138804091_T_G_b38	1.4e-7	0.33	Brain - Cortex
		ENSG00000044115.20	CTNNA1	chr5_138804091_T_G_b38	3.2e-7	-0.098	Thyroid
		ENSG00000120725.12	SIL1	chr5_138804091_T_G_b38	4.7e-7	0.40	Brain - Frontal Cortex (BA9)
		ENSG00000044115.20	CTNNA1	chr5_138804091_T_G_b38	4.8e-7	-0.15	Colon - Transverse
		ENSG00000120725.12	SIL1	chr5_138804091_T_G_b38	4.9e-7	0.44	Brain - Substantia nigra
		ENSG00000120725.12	SIL1	chr5_138804091_T_G_b38	5.2e-7	0.25	Pituitary
		ENSG00000120725.12	SIL1	chr5_138804091_T_G_b38	5.2e-7	0.14	Adipose - Subcutaneous
		ENSG00000120725.12	SIL1	chr5_138804091_T_G_b38	5.9e-7	0.16	Esophagus - Mucosa
		ENSG00000120725.12	SIL1	chr5_138804091_T_G_b38	0.0000010	0.23	Adrenal Gland
		ENSG00000120725.12	SIL1	chr5_138804091_T_G_b38	0.0000011	0.33	Brain - Caudate (basal ganglia)
		ENSG00000120725.12	SIL1	chr5_138804091_T_G_b38	0.0000016	0.11	Cells - Cultured fibroblasts
		ENSG00000044115.20	CTNNA1	chr5_138804091_T_G_b38	0.0000017	0.099	Testis
		ENSG00000120725.12	SIL1	chr5_138804091_T_G_b38	0.0000030	0.28	Brain - Cerebellum
		ENSG00000120725.12	SIL1	chr5_138804091_T_G_b38	0.0000045	0.34	Brain - Nucleus accumbens (basal ganglia)

		ENSG00000120725.12	SIL1	chr5_138804091_T_G_b38	0.0000050	0.19	Colon - Transverse
		ENSG00000044115.20	CTNNA1	chr5_138804091_T_G_b38	0.0000085	-0.12	Heart - Atrial Appendage
		ENSG00000044115.20	CTNNA1	chr5_138804091_T_G_b38	0.0000097	-0.088	Skin - Not Sun Exposed (Suprapubic)
		ENSG00000044115.20	CTNNA1	chr5_138804091_T_G_b38	0.000011	-0.14	Spleen
		ENSG00000044115.20	CTNNA1	chr5_138804091_T_G_b38	0.000017	-0.13	Muscle - Skeletal
		ENSG00000120725.12	SIL1	chr5_138804091_T_G_b38	0.000044	0.32	Brain - Anterior cingulate cortex (BA24)
		ENSG00000120725.12	SIL1	chr5_138804091_T_G_b38	0.000047	0.26	Artery - Coronary
		ENSG00000120725.12	SIL1	chr5_138804091_T_G_b38	0.000066	0.28	Brain - Putamen (basal ganglia)
		ENSG00000044115.20	CTNNA1	chr5_138804091_T_G_b38	0.000068	-0.073	Skin - Sun Exposed (Lower leg)
		ENSG00000044115.20	CTNNA1	chr5_138804091_T_G_b38	0.000078	-0.097	Adipose - Visceral (Omentum)
	rs13154573	ENSG00000253404.1	AC034243.1	chr5_138812375_T_C_b38	5.0e-109	1.1	Thyroid
		ENSG00000253404.1	AC034243.1	chr5_138812375_T_C_b38	6.3e-82	1.1	Skin - Sun Exposed (Lower leg)
		ENSG00000253404.1	AC034243.1	chr5_138812375_T_C_b38	8.0e-70	0.98	Adipose - Subcutaneous
		ENSG00000044115.20	CTNNA1	chr5_138812375_T_C_b38	1.6e-63	-0.47	Whole Blood
		ENSG00000253404.1	AC034243.1	chr5_138812375_T_C_b38	3.9e-61	1.0	Skin - Not Sun Exposed (Suprapubic)
		ENSG00000253404.1	AC034243.1	chr5_138812375_T_C_b38	6.8e-61	0.91	Nerve - Tibial
		ENSG00000253404.1	AC034243.1	chr5_138812375_T_C_b38	7.5e-59	0.99	Esophagus - Mucosa
		ENSG00000253404.1	AC034243.1	chr5_138812375_T_C_b38	1.4e-56	0.88	Lung
		ENSG00000253404.1	AC034243.1	chr5_138812375_T_C_b38	5.5e-56	1.0	Breast - Mammary Tissue
		ENSG00000253404.1	AC034243.1	chr5_138812375_T_C_b38	9.8e-53	0.92	Esophagus - Muscularis
		ENSG00000253404.1	AC034243.1	chr5_138812375_T_C_b38	3.8e-52	1.2	Pituitary
		ENSG00000253404.1	AC034243.1	chr5_138812375_T_C_b38	2.3e-49	0.89	Adipose - Visceral (Omentum)
		ENSG00000253404.1	AC034243.1	chr5_138812375_T_C_b38	2.3e-48	1.0	Heart - Atrial Appendage
		ENSG00000253404.1	AC034243.1	chr5_138812375_T_C_b38	5.1e-45	1.1	Pancreas
		ENSG00000253404.1	AC034243.1	chr5_138812375_T_C_b38	2.1e-44	0.93	Heart - Left Ventricle
		ENSG00000253404.1	AC034243.1	chr5_138812375_T_C_b38	3.3e-44	0.91	Stomach
		ENSG00000253404.1	AC034243.1	chr5_138812375_T_C_b38	4.5e-40	0.74	Cells - Cultured fibroblasts
		ENSG00000253404.1	AC034243.1	chr5_138812375_T_C_b38	3.7e-37	1.1	Adrenal Gland
		ENSG00000253404.1	AC034243.1	chr5_138812375_T_C_b38	8.2e-36	0.87	Colon - Transverse
		ENSG00000044115.20	CTNNA1	chr5_138812375_T_C_b38	1.5e-33	-0.25	Esophagus - Mucosa
		ENSG00000253404.1	AC034243.1	chr5_138812375_T_C_b38	2.9e-33	0.89	Prostate
		ENSG00000253404.1	AC034243.1	chr5_138812375_T_C_b38	5.1e-26	0.81	Esophagus - Gastroesophageal Junction
		ENSG00000253404.1	AC034243.1	chr5_138812375_T_C_b38	2.9e-25	1.0	Brain - Putamen (basal ganglia)
		ENSG00000253404.1	AC034243.1	chr5_138812375_T_C_b38	4.1e-24	1.1	Brain - Nucleus accumbens (basal ganglia)
		ENSG00000253404.1	AC034243.1	chr5_138812375_T_C_b38	1.0e-23	0.81	Colon - Sigmoid
		ENSG00000253404.1	AC034243.1	chr5_138812375_T_C_b38	2.1e-23	1.0	Brain - Caudate (basal ganglia)
		ENSG00000253404.1	AC034243.1	chr5_138812375_T_C_b38	1.3e-22	0.92	Spleen
		ENSG00000253404.1	AC034243.1	chr5_138812375_T_C_b38	4.9e-21	0.87	Brain - Cortex
		ENSG00000253404.1	AC034243.1	chr5_138812375_T_C_b38	2.4e-20	0.87	Liver
		ENSG00000253404.1	AC034243.1	chr5_138812375_T_C_b38	3.1e-19	0.46	Testis
		ENSG00000253404.1	AC034243.1	chr5_138812375_T_C_b38	7.2e-19	1.0	Brain - Spinal cord (cervical c-1)
		ENSG00000253404.1	AC034243.1	chr5_138812375_T_C_b38	1.3e-18	1.0	Ovary
		ENSG00000253404.1	AC034243.1	chr5_138812375_T_C_b38	1.9e-18	0.83	Artery - Coronary
		ENSG00000253404.1	AC034243.1	chr5_138812375_T_C_b38	2.0e-17	0.97	Brain - Substantia nigra
		ENSG00000253404.1	AC034243.1	chr5_138812375_T_C_b38	2.4e-17	0.93	Brain - Hippocampus
		ENSG00000120725.12	SIL1	chr5_138812375_T_C_b38	7.5e-17	0.22	Skin - Sun Exposed (Lower leg)
		ENSG00000253404.1	AC034243.1	chr5_138812375_T_C_b38	1.1e-16	1.2	Brain - Amygdala
		ENSG00000253404.1	AC034243.1	chr5_138812375_T_C_b38	1.5e-16	0.81	Brain - Frontal Cortex (BA9)
		ENSG00000253404.1	AC034243.1	chr5_138812375_T_C_b38	1.7e-16	0.89	Brain - Hypothalamus
		ENSG00000253404.1	AC034243.1	chr5_138812375_T_C_b38	1.9e-16	1.0	Minor Salivary Gland
		ENSG00000120725.12	SIL1	chr5_138812375_T_C_b38	5.5e-16	0.23	Skin - Not Sun Exposed (Suprapubic)
		ENSG00000253404.1	AC034243.1	chr5_138812375_T_C_b38	1.5e-14	1.0	Uterus
		ENSG00000253404.1	AC034243.1	chr5_138812375_T_C_b38	2.3e-14	0.78	Brain - Anterior cingulate cortex (BA24)
		ENSG00000120725.12	SIL1	chr5_138812375_T_C_b38	2.8e-14	0.19	Nerve - Tibial
		ENSG00000120725.12	SIL1	chr5_138812375_T_C_b38	6.7e-14	0.30	Esophagus - Muscularis
		ENSG00000253404.1	AC034243.1	chr5_138812375_T_C_b38	6.9e-14	0.74	Small Intestine - Terminal Ileum
		ENSG00000120725.12	SIL1	chr5_138812375_T_C_b38	1.1e-13	0.27	Artery - Aorta
		ENSG00000253404.1	AC034243.1	chr5_138812375_T_C_b38	3.4e-12	0.94	Vagina
		ENSG00000120725.12	SIL1	chr5_138812375_T_C_b38	3.6e-11	0.27	Stomach
		ENSG00000120725.12	SIL1	chr5_138812375_T_C_b38	4.0e-11	0.14	Thyroid
		ENSG00000120725.12	SIL1	chr5_138812375_T_C_b38	1.9e-10	0.15	Lung
		ENSG00000253404.1	AC034243.1	chr5_138812375_T_C_b38	2.0e-10	1.0	Kidney - Cortex
		ENSG00000044115.20	CTNNA1	chr5_138812375_T_C_b38	3.3e-9	-0.14	Cells - Cultured fibroblasts
		ENSG00000120725.12	SIL1	chr5_138812375_T_C_b38	4.1e-9	0.15	Artery - Tibial
		ENSG00000044115.20	CTNNA1	chr5_138812375_T_C_b38	4.3e-9	-0.11	Nerve - Tibial
		ENSG00000120725.12	SIL1	chr5_138812375_T_C_b38	1.0e-8	0.30	Esophagus - Gastroesophageal Junction
		ENSG00000120725.12	SIL1	chr5_138812375_T_C_b38	4.5e-8	0.14	Adipose - Visceral (Omentum)
		ENSG00000120725.12	SIL1	chr5_138812375_T_C_b38	1.4e-7	0.33	Brain - Cortex
		ENSG00000044115.20	CTNNA1	chr5_138812375_T_C_b38	1.4e-7	-0.14	Breast - Mammary Tissue
		ENSG00000120725.12	SIL1	chr5_138812375_T_C_b38	2.0e-7	0.16	Esophagus - Mucosa
		ENSG00000044115.20	CTNNA1	chr5_138812375_T_C_b38	2.5e-7	-0.099	Thyroid
		ENSG00000120725.12	SIL1	chr5_138812375_T_C_b38	3.3e-7	0.14	Adipose - Subcutaneous
		ENSG00000120725.12	SIL1	chr5_138812375_T_C_b38	4.7e-7	0.40	Brain - Frontal Cortex (BA9)
		ENSG00000120725.12	SIL1	chr5_138812375_T_C_b38	4.9e-7	0.44	Brain - Substantia nigra
		ENSG00000120725.12	SIL1	chr5_138812375_T_C_b38	5.2e-7	0.25	Pituitary
		ENSG00000044115.20	CTNNA1	chr5_138812375_T_C_b38	6.7e-7	-0.15	Colon - Transverse
		ENSG00000120725.12	SIL1	chr5_138812375_T_C_b38	0.0000010	0.23	Adrenal Gland
		ENSG00000120725.12	SIL1	chr5_138812375_T_C_b38	0.0000011	0.33	Brain - Caudate (basal ganglia)
		ENSG00000044115.20	CTNNA1	chr5_138812375_T_C_b38	0.0000015	0.099	Testis
		ENSG00000120725.12	SIL1	chr5_138812375_T_C_b38	0.0000016	0.11	Cells - Cultured fibroblasts
		ENSG00000120725.12	SIL1	chr5_138812375_T_C_b38	0.0000030	0.28	Brain - Cerebellum
		ENSG00000120725.12	SIL1	chr5_138812375_T_C_b38	0.0000045	0.34	Brain - Nucleus accumbens (basal ganglia)

		ENSG00000120725.12	SIL1	chr5_138812375_T_C_b38	0.0000054	0.19	Colon - Transverse
		ENSG00000044115.20	CTNNA1	chr5_138812375_T_C_b38	0.0000060	-0.090	Skin - Not Sun Exposed (Suprapubic)
		ENSG00000044115.20	CTNNA1	chr5_138812375_T_C_b38	0.0000085	-0.12	Heart - Atrial Appendage
		ENSG00000044115.20	CTNNA1	chr5_138812375_T_C_b38	0.000011	-0.14	Spleen
		ENSG00000044115.20	CTNNA1	chr5_138812375_T_C_b38	0.000024	-0.13	Muscle - Skeletal
		ENSG00000044115.20	CTNNA1	chr5_138812375_T_C_b38	0.000039	-0.075	Skin - Sun Exposed (Lower leg)
		ENSG00000120725.12	SIL1	chr5_138812375_T_C_b38	0.000044	0.32	Brain - Anterior cingulate cortex (BA24)
		ENSG00000120725.12	SIL1	chr5_138812375_T_C_b38	0.000047	0.26	Artery - Coronary
		ENSG00000120725.12	SIL1	chr5_138812375_T_C_b38	0.000066	0.28	Brain - Putamen (basal ganglia)
	rs167009	ENSG00000253404.1	AC034243.1	chr5_138864768_G_C_b38	4.5e-131	-1.2	Thyroid
		ENSG00000253404.1	AC034243.1	chr5_138864768_G_C_b38	8.5e-93	-1.1	Skin - Sun Exposed (Lower leg)
		ENSG00000253404.1	AC034243.1	chr5_138864768_G_C_b38	6.0e-86	-1.0	Adipose - Subcutaneous
		ENSG00000253404.1	AC034243.1	chr5_138864768_G_C_b38	3.0e-74	-1.1	Esophagus - Mucosa
		ENSG00000044115.20	CTNNA1	chr5_138864768_G_C_b38	7.6e-74	0.49	Whole Blood
		ENSG00000253404.1	AC034243.1	chr5_138864768_G_C_b38	3.7e-70	-1.0	Skin - Not Sun Exposed (Suprapubic)
		ENSG00000253404.1	AC034243.1	chr5_138864768_G_C_b38	1.3e-69	-0.94	Nerve - Tibial
		ENSG00000253404.1	AC034243.1	chr5_138864768_G_C_b38	2.8e-65	-1.2	Pituitary
		ENSG00000253404.1	AC034243.1	chr5_138864768_G_C_b38	3.3e-65	-0.92	Lung
		ENSG00000253404.1	AC034243.1	chr5_138864768_G_C_b38	1.3e-63	-1.0	Breast - Mammary Tissue
		ENSG00000253404.1	AC034243.1	chr5_138864768_G_C_b38	3.3e-57	-0.92	Adipose - Visceral (Omentum)
		ENSG00000253404.1	AC034243.1	chr5_138864768_G_C_b38	3.7e-57	-0.92	Esophagus - Muscularis
		ENSG00000253404.1	AC034243.1	chr5_138864768_G_C_b38	8.0e-56	-1.2	Pancreas
		ENSG00000253404.1	AC034243.1	chr5_138864768_G_C_b38	2.2e-55	-1.0	Heart - Atrial Appendage
		ENSG00000253404.1	AC034243.1	chr5_138864768_G_C_b38	2.7e-54	-0.95	Stomach
		ENSG00000253404.1	AC034243.1	chr5_138864768_G_C_b38	1.4e-49	-0.95	Heart - Left Ventricle
		ENSG00000253404.1	AC034243.1	chr5_138864768_G_C_b38	3.4e-48	-1.2	Adrenal Gland
		ENSG00000253404.1	AC034243.1	chr5_138864768_G_C_b38	2.1e-46	-0.77	Cells - Cultured fibroblasts
		ENSG00000253404.1	AC034243.1	chr5_138864768_G_C_b38	6.3e-42	-0.89	Colon - Transverse
		ENSG00000044115.20	CTNNA1	chr5_138864768_G_C_b38	2.2e-40	0.27	Esophagus - Mucosa
		ENSG00000253404.1	AC034243.1	chr5_138864768_G_C_b38	4.6e-39	-0.93	Prostate
		ENSG00000253404.1	AC034243.1	chr5_138864768_G_C_b38	5.0e-34	-0.88	Esophagus - Gastroesophageal Junction
		ENSG00000253404.1	AC034243.1	chr5_138864768_G_C_b38	1.1e-30	-0.88	Colon - Sigmoid
		ENSG00000253404.1	AC034243.1	chr5_138864768_G_C_b38	8.0e-29	-1.0	Spleen
		ENSG00000253404.1	AC034243.1	chr5_138864768_G_C_b38	1.0e-28	-1.0	Brain - Putamen (basal ganglia)
		ENSG00000253404.1	AC034243.1	chr5_138864768_G_C_b38	1.2e-28	-1.1	Brain - Caudate (basal ganglia)
		ENSG00000253404.1	AC034243.1	chr5_138864768_G_C_b38	1.7e-26	-0.95	Liver
		ENSG00000253404.1	AC034243.1	chr5_138864768_G_C_b38	6.1e-26	-1.1	Ovary
		ENSG00000253404.1	AC034243.1	chr5_138864768_G_C_b38	1.2e-25	-1.0	Brain - Nucleus accumbens (basal ganglia)
		ENSG00000253404.1	AC034243.1	chr5_138864768_G_C_b38	8.6e-24	-0.49	Testis
		ENSG00000253404.1	AC034243.1	chr5_138864768_G_C_b38	9.2e-23	-0.87	Brain - Cortex
		ENSG00000253404.1	AC034243.1	chr5_138864768_G_C_b38	2.5e-22	-1.1	Minor Salivary Gland
		ENSG00000253404.1	AC034243.1	chr5_138864768_G_C_b38	1.5e-21	-0.87	Artery - Coronary
		ENSG00000253404.1	AC034243.1	chr5_138864768_G_C_b38	7.6e-20	-1.0	Brain - Spinal cord (cervical c-1)
		ENSG00000253404.1	AC034243.1	chr5_138864768_G_C_b38	3.2e-19	-0.94	Brain - Hippocampus
		ENSG00000253404.1	AC034243.1	chr5_138864768_G_C_b38	1.7e-18	-0.99	Brain - Substantia nigra
		ENSG00000253404.1	AC034243.1	chr5_138864768_G_C_b38	3.4e-18	-0.90	Brain - Hypothalamus
		ENSG00000120725.12	SIL1	chr5_138864768_G_C_b38	3.7e-18	-0.33	Esophagus - Muscularis
		ENSG00000120725.12	SIL1	chr5_138864768_G_C_b38	9.7e-18	-0.21	Nerve - Tibial
		ENSG00000253404.1	AC034243.1	chr5_138864768_G_C_b38	3.7e-17	-1.1	Uterus
		ENSG00000253404.1	AC034243.1	chr5_138864768_G_C_b38	5.7e-17	-1.1	Brain - Amygdala
		ENSG00000253404.1	AC034243.1	chr5_138864768_G_C_b38	5.8e-17	-0.79	Brain - Frontal Cortex (BA9)
		ENSG00000120725.12	SIL1	chr5_138864768_G_C_b38	6.4e-17	-0.22	Skin - Sun Exposed (Lower leg)
		ENSG00000253404.1	AC034243.1	chr5_138864768_G_C_b38	1.9e-16	-0.80	Small Intestine - Terminal Ileum
		ENSG00000253404.1	AC034243.1	chr5_138864768_G_C_b38	4.5e-16	-1.1	Vagina
		ENSG00000253404.1	AC034243.1	chr5_138864768_G_C_b38	5.3e-16	-0.79	Brain - Anterior cingulate cortex (BA24)
		ENSG00000120725.12	SIL1	chr5_138864768_G_C_b38	1.3e-15	-0.22	Skin - Not Sun Exposed (Suprapubic)
		ENSG00000120725.12	SIL1	chr5_138864768_G_C_b38	2.2e-15	-0.29	Artery - Aorta
		ENSG00000120725.12	SIL1	chr5_138864768_G_C_b38	4.1e-14	-0.29	Stomach
		ENSG00000253404.1	AC034243.1	chr5_138864768_G_C_b38	1.4e-12	-1.1	Kidney - Cortex
		ENSG00000120725.12	SIL1	chr5_138864768_G_C_b38	3.9e-12	-0.14	Thyroid
		ENSG00000120725.12	SIL1	chr5_138864768_G_C_b38	4.2e-10	-0.16	Artery - Tibial
		ENSG00000120725.12	SIL1	chr5_138864768_G_C_b38	4.8e-10	-0.17	Adipose - Subcutaneous
		ENSG00000120725.12	SIL1	chr5_138864768_G_C_b38	1.7e-9	-0.15	Adipose - Visceral (Omentum)
		ENSG00000120725.12	SIL1	chr5_138864768_G_C_b38	2.6e-9	-0.14	Lung
		ENSG00000044115.20	CTNNA1	chr5_138864768_G_C_b38	3.0e-9	0.11	Nerve - Tibial
		ENSG00000120725.12	SIL1	chr5_138864768_G_C_b38	3.4e-9	-0.30	Esophagus - Gastroesophageal Junction
		ENSG00000044115.20	CTNNA1	chr5_138864768_G_C_b38	6.8e-9	0.13	Cells - Cultured fibroblasts
		ENSG00000120725.12	SIL1	chr5_138864768_G_C_b38	7.5e-9	-0.43	Brain - Frontal Cortex (BA9)
		ENSG00000044115.20	CTNNA1	chr5_138864768_G_C_b38	6.1e-8	0.15	Breast - Mammary Tissue
		ENSG00000120725.12	SIL1	chr5_138864768_G_C_b38	6.6e-8	-0.26	Pituitary
		ENSG00000120725.12	SIL1	chr5_138864768_G_C_b38	7.5e-8	-0.12	Cells - Cultured fibroblasts
		ENSG00000044115.20	CTNNA1	chr5_138864768_G_C_b38	1.6e-7	0.15	Colon - Transverse
		ENSG00000044115.20	CTNNA1	chr5_138864768_G_C_b38	3.3e-7	-0.10	Testis
		ENSG00000120725.12	SIL1	chr5_138864768_G_C_b38	3.5e-7	-0.29	Brain - Cerebellum
		ENSG00000120725.12	SIL1	chr5_138864768_G_C_b38	3.9e-7	-0.31	Brain - Cortex
		ENSG00000120725.12	SIL1	chr5_138864768_G_C_b38	4.2e-7	-0.33	Brain - Caudate (basal ganglia)
		ENSG00000044115.20	CTNNA1	chr5_138864768_G_C_b38	4.6e-7	0.098	Skin - Not Sun Exposed (Suprapubic)
		ENSG00000044115.20	CTNNA1	chr5_138864768_G_C_b38	4.9e-7	0.095	Thyroid
		ENSG00000120725.12	SIL1	chr5_138864768_G_C_b38	7.5e-7	-0.15	Esophagus - Mucosa
		ENSG00000044115.20	CTNNA1	chr5_138864768_G_C_b38	0.0000013	0.21	Brain - Cerebellum
		ENSG00000044115.20	CTNNA1	chr5_138864768_G_C_b38	0.0000018	0.13	Heart - Atrial Appendage
		ENSG00000120725.12	SIL1	chr5_138864768_G_C_b38	0.0000024	-0.34	Brain - Nucleus accumbens (basal ganglia)

		ENSG00000120725.12	SIL1	chr5_138864768_G_C_b38	0.0000025	-0.22	Adrenal Gland
		ENSG00000044115.20	CTNNA1	chr5_138864768_G_C_b38	0.0000029	0.14	Muscle - Skeletal
		ENSG00000120725.12	SIL1	chr5_138864768_G_C_b38	0.0000034	-0.41	Brain - Substantia nigra
		ENSG00000044115.20	CTNNA1	chr5_138864768_G_C_b38	0.0000040	0.083	Skin - Sun Exposed (Lower leg)
		ENSG00000044115.20	CTNNA1	chr5_138864768_G_C_b38	0.0000044	0.14	Spleen
		ENSG00000120725.12	SIL1	chr5_138864768_G_C_b38	0.000010	-0.17	Colon - Transverse
		ENSG00000120725.12	SIL1	chr5_138864768_G_C_b38	0.000014	-0.13	Breast - Mammary Tissue
		ENSG00000120725.12	SIL1	chr5_138864768_G_C_b38	0.000023	-0.13	Heart - Left Ventricle
		ENSG00000120725.12	SIL1	chr5_138864768_G_C_b38	0.000024	-0.32	Brain - Anterior cingulate cortex (BA24)
		ENSG00000044115.20	CTNNA1	chr5_138864768_G_C_b38	0.000025	0.20	Brain - Cerebellar Hemisphere
		ENSG00000120725.12	SIL1	chr5_138864768_G_C_b38	0.000030	-0.26	Artery - Coronary
		ENSG00000044115.20	CTNNA1	chr5_138864768_G_C_b38	0.000039	0.12	Pancreas
		ENSG00000044115.20	CTNNA1	chr5_138864768_G_C_b38	0.000057	0.10	Adipose - Subcutaneous
		ENSG00000120725.12	SIL1	chr5_138864768_G_C_b38	0.000069	-0.15	Pancreas
		ENSG00000120725.12	SIL1	chr5_138864768_G_C_b38	0.000075	-0.26	Brain - Putamen (basal ganglia)
	rs2292268	ENSG00000250159.6	RP11-381K20.2	chr5_138868792_A_G_b38	8.0e-7	0.58	Pituitary
		ENSG00000253404.1	AC034243.1	chr5_138868792_A_G_b38	0.0000047	-0.44	Thyroid
		ENSG00000253404.1	AC034243.1	chr5_138868792_A_G_b38	0.0000052	-0.43	Skin - Sun Exposed (Lower leg)
		ENSG00000253404.1	AC034243.1	chr5_138868792_A_G_b38	0.000014	-0.43	Nerve - Tibial
		ENSG00000253404.1	AC034243.1	chr5_138868792_A_G_b38	0.000032	-0.46	Esophagus - Mucosa
		ENSG00000253404.1	AC034243.1	chr5_138868792_A_G_b38	0.000035	-0.60	Prostate
		ENSG00000253404.1	AC034243.1	chr5_138868792_A_G_b38	0.000065	-0.50	Breast - Mammary Tissue
		ENSG00000044115.20	CTNNA1	chr5_138868792_A_G_b38	0.000069	0.19	Whole Blood
		ENSG00000253404.1	AC034243.1	chr5_138868792_A_G_b38	0.000080	-0.53	Pancreas
		ENSG00000253404.1	AC034243.1	chr5_138868792_A_G_b38	0.000090	-0.40	Esophagus - Muscularis
	rs288040	ENSG00000253404.1	AC034243.1	chr5_138869205_A_T_b38	1.7e-129	1.2	Thyroid
		ENSG00000253404.1	AC034243.1	chr5_138869205_A_T_b38	8.5e-93	1.1	Skin - Sun Exposed (Lower leg)
		ENSG00000253404.1	AC034243.1	chr5_138869205_A_T_b38	5.2e-85	1.0	Adipose - Subcutaneous
		ENSG00000253404.1	AC034243.1	chr5_138869205_A_T_b38	2.9e-73	1.1	Esophagus - Mucosa
		ENSG00000044115.20	CTNNA1	chr5_138869205_A_T_b38	8.7e-73	-0.49	Whole Blood
		ENSG00000253404.1	AC034243.1	chr5_138869205_A_T_b38	3.7e-70	1.0	Skin - Not Sun Exposed (Suprapubic)
		ENSG00000253404.1	AC034243.1	chr5_138869205_A_T_b38	2.2e-69	0.94	Nerve - Tibial
		ENSG00000253404.1	AC034243.1	chr5_138869205_A_T_b38	3.3e-65	0.92	Lung
		ENSG00000253404.1	AC034243.1	chr5_138869205_A_T_b38	1.3e-63	1.0	Breast - Mammary Tissue
		ENSG00000253404.1	AC034243.1	chr5_138869205_A_T_b38	2.3e-63	1.2	Pituitary
		ENSG00000253404.1	AC034243.1	chr5_138869205_A_T_b38	3.7e-57	0.92	Esophagus - Muscularis
		ENSG00000253404.1	AC034243.1	chr5_138869205_A_T_b38	1.4e-56	0.91	Adipose - Visceral (Omentum)
		ENSG00000253404.1	AC034243.1	chr5_138869205_A_T_b38	8.0e-56	1.2	Pancreas
		ENSG00000253404.1	AC034243.1	chr5_138869205_A_T_b38	2.2e-55	1.0	Heart - Atrial Appendage
		ENSG00000253404.1	AC034243.1	chr5_138869205_A_T_b38	2.7e-54	0.95	Stomach
		ENSG00000253404.1	AC034243.1	chr5_138869205_A_T_b38	1.4e-49	0.95	Heart - Left Ventricle
		ENSG00000253404.1	AC034243.1	chr5_138869205_A_T_b38	3.4e-48	1.2	Adrenal Gland
		ENSG00000253404.1	AC034243.1	chr5_138869205_A_T_b38	5.4e-46	0.77	Cells - Cultured fibroblasts
		ENSG00000253404.1	AC034243.1	chr5_138869205_A_T_b38	6.3e-42	0.89	Colon - Transverse
		ENSG00000044115.20	CTNNA1	chr5_138869205_A_T_b38	1.2e-40	-0.27	Esophagus - Mucosa
		ENSG00000253404.1	AC034243.1	chr5_138869205_A_T_b38	4.6e-39	0.93	Prostate
		ENSG00000253404.1	AC034243.1	chr5_138869205_A_T_b38	5.8e-34	0.88	Esophagus - Gastroesophageal Junction
		ENSG00000253404.1	AC034243.1	chr5_138869205_A_T_b38	1.1e-30	0.88	Colon - Sigmoid
		ENSG00000253404.1	AC034243.1	chr5_138869205_A_T_b38	8.0e-29	1.0	Spleen
		ENSG00000253404.1	AC034243.1	chr5_138869205_A_T_b38	2.0e-28	1.1	Brain - Caudate (basal ganglia)
		ENSG00000253404.1	AC034243.1	chr5_138869205_A_T_b38	1.7e-27	1.0	Brain - Putamen (basal ganglia)
		ENSG00000253404.1	AC034243.1	chr5_138869205_A_T_b38	6.1e-26	1.1	Ovary
		ENSG00000253404.1	AC034243.1	chr5_138869205_A_T_b38	3.7e-25	0.93	Liver
		ENSG00000253404.1	AC034243.1	chr5_138869205_A_T_b38	7.3e-25	1.0	Brain - Nucleus accumbens (basal ganglia)
		ENSG00000253404.1	AC034243.1	chr5_138869205_A_T_b38	2.0e-23	0.48	Testis
		ENSG00000253404.1	AC034243.1	chr5_138869205_A_T_b38	1.2e-22	0.87	Brain - Cortex
		ENSG00000253404.1	AC034243.1	chr5_138869205_A_T_b38	2.5e-22	1.1	Minor Salivary Gland
		ENSG00000253404.1	AC034243.1	chr5_138869205_A_T_b38	1.5e-21	0.87	Artery - Coronary
		ENSG00000253404.1	AC034243.1	chr5_138869205_A_T_b38	7.6e-20	1.0	Brain - Spinal cord (cervical c-1)
		ENSG00000253404.1	AC034243.1	chr5_138869205_A_T_b38	9.7e-20	0.94	Brain - Hippocampus
		ENSG00000253404.1	AC034243.1	chr5_138869205_A_T_b38	1.7e-18	0.99	Brain - Substantia nigra
		ENSG00000120725.12	SIL1	chr5_138869205_A_T_b38	3.7e-18	0.33	Esophagus - Muscularis
		ENSG00000120725.12	SIL1	chr5_138869205_A_T_b38	1.3e-17	0.21	Nerve - Tibial
		ENSG00000253404.1	AC034243.1	chr5_138869205_A_T_b38	1.4e-17	0.88	Brain - Hypothalamus
		ENSG00000253404.1	AC034243.1	chr5_138869205_A_T_b38	3.7e-17	1.1	Uterus
		ENSG00000120725.12	SIL1	chr5_138869205_A_T_b38	6.4e-17	0.22	Skin - Sun Exposed (Lower leg)
		ENSG00000253404.1	AC034243.1	chr5_138869205_A_T_b38	1.2e-16	0.78	Brain - Frontal Cortex (BA9)
		ENSG00000253404.1	AC034243.1	chr5_138869205_A_T_b38	1.9e-16	0.80	Small Intestine - Terminal Ileum
		ENSG00000253404.1	AC034243.1	chr5_138869205_A_T_b38	2.1e-16	1.0	Brain - Amygdala
		ENSG00000253404.1	AC034243.1	chr5_138869205_A_T_b38	4.5e-16	1.1	Vagina
		ENSG00000253404.1	AC034243.1	chr5_138869205_A_T_b38	5.3e-16	0.79	Brain - Anterior cingulate cortex (BA24)
		ENSG00000120725.12	SIL1	chr5_138869205_A_T_b38	1.3e-15	0.22	Skin - Not Sun Exposed (Suprapubic)
		ENSG00000120725.12	SIL1	chr5_138869205_A_T_b38	2.2e-15	0.29	Artery - Aorta
		ENSG00000120725.12	SIL1	chr5_138869205_A_T_b38	4.1e-14	0.29	Stomach
		ENSG00000253404.1	AC034243.1	chr5_138869205_A_T_b38	1.4e-12	1.1	Kidney - Cortex
		ENSG00000120725.12	SIL1	chr5_138869205_A_T_b38	4.7e-12	0.14	Thyroid
		ENSG00000120725.12	SIL1	chr5_138869205_A_T_b38	4.2e-10	0.16	Artery - Tibial
		ENSG00000120725.12	SIL1	chr5_138869205_A_T_b38	6.0e-10	0.17	Adipose - Subcutaneous
		ENSG00000120725.12	SIL1	chr5_138869205_A_T_b38	2.6e-9	0.14	Lung
		ENSG00000120725.12	SIL1	chr5_138869205_A_T_b38	2.7e-9	0.15	Adipose - Visceral (Omentum)
		ENSG00000044115.20	CTNNA1	chr5_138869205_A_T_b38	2.8e-9	-0.11	Nerve - Tibial
		ENSG00000044115.20	CTNNA1	chr5_138869205_A_T_b38	3.5e-9	-0.13	Cells - Cultured fibroblasts

		ENSG00000120725.12	SIL1	chr5_138869205_A_T_b38	4.7e-9	0.30	Esophagus - Gastroesophageal Junction
		ENSG00000120725.12	SIL1	chr5_138869205_A_T_b38	8.6e-9	0.43	Brain - Frontal Cortex (BA9)
		ENSG00000120725.12	SIL1	chr5_138869205_A_T_b38	2.4e-8	0.27	Pituitary
		ENSG00000044115.20	CTNNA1	chr5_138869205_A_T_b38	6.1e-8	-0.15	Breast - Mammary Tissue
		ENSG00000120725.12	SIL1	chr5_138869205_A_T_b38	8.2e-8	0.12	Cells - Cultured fibroblasts
		ENSG00000044115.20	CTNNA1	chr5_138869205_A_T_b38	1.6e-7	-0.15	Colon - Transverse
		ENSG00000120725.12	SIL1	chr5_138869205_A_T_b38	3.4e-7	0.33	Brain - Caudate (basal ganglia)
		ENSG00000120725.12	SIL1	chr5_138869205_A_T_b38	4.1e-7	0.29	Brain - Cerebellum
		ENSG00000044115.20	CTNNA1	chr5_138869205_A_T_b38	4.3e-7	0.10	Testis
		ENSG00000044115.20	CTNNA1	chr5_138869205_A_T_b38	4.6e-7	-0.098	Skin - Not Sun Exposed (Suprapubic)
		ENSG00000044115.20	CTNNA1	chr5_138869205_A_T_b38	5.1e-7	-0.095	Thyroid
		ENSG00000120725.12	SIL1	chr5_138869205_A_T_b38	9.1e-7	0.30	Brain - Cortex
		ENSG00000120725.12	SIL1	chr5_138869205_A_T_b38	0.0000012	0.15	Esophagus - Mucosa
		ENSG00000044115.20	CTNNA1	chr5_138869205_A_T_b38	0.0000018	-0.13	Heart - Atrial Appendage
		ENSG00000120725.12	SIL1	chr5_138869205_A_T_b38	0.0000025	0.34	Brain - Nucleus accumbens (basal ganglia)
		ENSG00000120725.12	SIL1	chr5_138869205_A_T_b38	0.0000025	0.22	Adrenal Gland
		ENSG00000044115.20	CTNNA1	chr5_138869205_A_T_b38	0.0000029	-0.14	Muscle - Skeletal
		ENSG00000120725.12	SIL1	chr5_138869205_A_T_b38	0.0000034	0.41	Brain - Substantia nigra
		ENSG00000044115.20	CTNNA1	chr5_138869205_A_T_b38	0.0000034	-0.20	Brain - Cerebellum
		ENSG00000044115.20	CTNNA1	chr5_138869205_A_T_b38	0.0000040	-0.083	Skin - Sun Exposed (Lower leg)
		ENSG00000044115.20	CTNNA1	chr5_138869205_A_T_b38	0.0000044	-0.14	Spleen
		ENSG00000120725.12	SIL1	chr5_138869205_A_T_b38	0.000010	0.17	Colon - Transverse
		ENSG00000120725.12	SIL1	chr5_138869205_A_T_b38	0.000014	0.13	Breast - Mammary Tissue
		ENSG00000120725.12	SIL1	chr5_138869205_A_T_b38	0.000023	0.13	Heart - Left Ventricle
		ENSG00000120725.12	SIL1	chr5_138869205_A_T_b38	0.000024	0.32	Brain - Anterior cingulate cortex (BA24)
		ENSG00000044115.20	CTNNA1	chr5_138869205_A_T_b38	0.000030	-0.20	Brain - Cerebellar Hemisphere
		ENSG00000120725.12	SIL1	chr5_138869205_A_T_b38	0.000030	0.26	Artery - Coronary
		ENSG00000044115.20	CTNNA1	chr5_138869205_A_T_b38	0.000035	-0.10	Adipose - Subcutaneous
		ENSG00000044115.20	CTNNA1	chr5_138869205_A_T_b38	0.000039	-0.12	Pancreas
		ENSG00000120725.12	SIL1	chr5_138869205_A_T_b38	0.000069	0.15	Pancreas
		ENSG00000120725.12	SIL1	chr5_138869205_A_T_b38	0.000091	0.26	Brain - Putamen (basal ganglia)
	rs288037	ENSG00000253404.1	AC034243.1	chr5_138872752_A_G_b38	4.5e-131	1.2	Thyroid
		ENSG00000253404.1	AC034243.1	chr5_138872752_A_G_b38	8.5e-93	1.1	Skin - Sun Exposed (Lower leg)
		ENSG00000253404.1	AC034243.1	chr5_138872752_A_G_b38	6.0e-86	1.0	Adipose - Subcutaneous
		ENSG00000253404.1	AC034243.1	chr5_138872752_A_G_b38	3.0e-74	1.1	Esophagus - Mucosa
		ENSG00000044115.20	CTNNA1	chr5_138872752_A_G_b38	7.6e-74	-0.49	Whole Blood
		ENSG00000253404.1	AC034243.1	chr5_138872752_A_G_b38	3.7e-70	1.0	Skin - Not Sun Exposed (Suprapubic)
		ENSG00000253404.1	AC034243.1	chr5_138872752_A_G_b38	1.3e-69	0.94	Nerve - Tibial
		ENSG00000253404.1	AC034243.1	chr5_138872752_A_G_b38	2.8e-65	1.2	Pituitary
		ENSG00000253404.1	AC034243.1	chr5_138872752_A_G_b38	3.3e-65	0.92	Lung
		ENSG00000253404.1	AC034243.1	chr5_138872752_A_G_b38	1.3e-63	1.0	Breast - Mammary Tissue
		ENSG00000253404.1	AC034243.1	chr5_138872752_A_G_b38	3.3e-57	0.92	Adipose - Visceral (Omentum)
		ENSG00000253404.1	AC034243.1	chr5_138872752_A_G_b38	3.7e-57	0.92	Esophagus - Muscularis
		ENSG00000253404.1	AC034243.1	chr5_138872752_A_G_b38	8.0e-56	1.2	Pancreas
		ENSG00000253404.1	AC034243.1	chr5_138872752_A_G_b38	2.2e-55	1.0	Heart - Atrial Appendage
		ENSG00000253404.1	AC034243.1	chr5_138872752_A_G_b38	2.7e-54	0.95	Stomach
		ENSG00000253404.1	AC034243.1	chr5_138872752_A_G_b38	1.4e-49	0.95	Heart - Left Ventricle
		ENSG00000253404.1	AC034243.1	chr5_138872752_A_G_b38	3.4e-48	1.2	Adrenal Gland
		ENSG00000253404.1	AC034243.1	chr5_138872752_A_G_b38	2.1e-46	0.77	Cells - Cultured fibroblasts
		ENSG00000253404.1	AC034243.1	chr5_138872752_A_G_b38	6.3e-42	0.89	Colon - Transverse
		ENSG00000044115.20	CTNNA1	chr5_138872752_A_G_b38	2.2e-40	-0.27	Esophagus - Mucosa
		ENSG00000253404.1	AC034243.1	chr5_138872752_A_G_b38	4.6e-39	0.93	Prostate
		ENSG00000253404.1	AC034243.1	chr5_138872752_A_G_b38	5.0e-34	0.88	Esophagus - Gastroesophageal Junction
		ENSG00000253404.1	AC034243.1	chr5_138872752_A_G_b38	1.1e-30	0.88	Colon - Sigmoid
		ENSG00000253404.1	AC034243.1	chr5_138872752_A_G_b38	8.0e-29	1.0	Spleen
		ENSG00000253404.1	AC034243.1	chr5_138872752_A_G_b38	1.0e-28	1.0	Brain - Putamen (basal ganglia)
		ENSG00000253404.1	AC034243.1	chr5_138872752_A_G_b38	1.2e-28	1.1	Brain - Caudate (basal ganglia)
		ENSG00000253404.1	AC034243.1	chr5_138872752_A_G_b38	1.7e-26	0.95	Liver
		ENSG00000253404.1	AC034243.1	chr5_138872752_A_G_b38	6.1e-26	1.1	Ovary
		ENSG00000253404.1	AC034243.1	chr5_138872752_A_G_b38	1.2e-25	1.0	Brain - Nucleus accumbens (basal ganglia)
		ENSG00000253404.1	AC034243.1	chr5_138872752_A_G_b38	8.6e-24	0.49	Testis
		ENSG00000253404.1	AC034243.1	chr5_138872752_A_G_b38	9.2e-23	0.87	Brain - Cortex
		ENSG00000253404.1	AC034243.1	chr5_138872752_A_G_b38	2.5e-22	1.1	Minor Salivary Gland
		ENSG00000253404.1	AC034243.1	chr5_138872752_A_G_b38	1.5e-21	0.87	Artery - Coronary
		ENSG00000253404.1	AC034243.1	chr5_138872752_A_G_b38	7.6e-20	1.0	Brain - Spinal cord (cervical c-1)
		ENSG00000253404.1	AC034243.1	chr5_138872752_A_G_b38	3.2e-19	0.94	Brain - Hippocampus
		ENSG00000253404.1	AC034243.1	chr5_138872752_A_G_b38	1.7e-18	0.99	Brain - Substantia nigra
		ENSG00000253404.1	AC034243.1	chr5_138872752_A_G_b38	3.4e-18	0.90	Brain - Hypothalamus
		ENSG00000120725.12	SIL1	chr5_138872752_A_G_b38	3.7e-18	0.33	Esophagus - Muscularis
		ENSG00000120725.12	SIL1	chr5_138872752_A_G_b38	9.7e-18	0.21	Nerve - Tibial
		ENSG00000253404.1	AC034243.1	chr5_138872752_A_G_b38	3.7e-17	1.1	Uterus
		ENSG00000253404.1	AC034243.1	chr5_138872752_A_G_b38	5.7e-17	1.1	Brain - Amygdala
		ENSG00000253404.1	AC034243.1	chr5_138872752_A_G_b38	5.8e-17	0.79	Brain - Frontal Cortex (BA9)
		ENSG00000120725.12	SIL1	chr5_138872752_A_G_b38	6.4e-17	0.22	Skin - Sun Exposed (Lower leg)
		ENSG00000253404.1	AC034243.1	chr5_138872752_A_G_b38	1.9e-16	0.80	Small Intestine - Terminal Ileum
		ENSG00000253404.1	AC034243.1	chr5_138872752_A_G_b38	4.5e-16	1.1	Vagina
		ENSG00000253404.1	AC034243.1	chr5_138872752_A_G_b38	5.3e-16	0.79	Brain - Anterior cingulate cortex (BA24)
		ENSG00000120725.12	SIL1	chr5_138872752_A_G_b38	1.3e-15	0.22	Skin - Not Sun Exposed (Suprapubic)
		ENSG00000120725.12	SIL1	chr5_138872752_A_G_b38	2.2e-15	0.29	Artery - Aorta
		ENSG00000120725.12	SIL1	chr5_138872752_A_G_b38	4.1e-14	0.29	Stomach
		ENSG00000253404.1	AC034243.1	chr5_138872752_A_G_b38	1.4e-12	1.1	Kidney - Cortex
		ENSG00000120725.12	SIL1	chr5_138872752_A_G_b38	3.9e-12	0.14	Thyroid

		ENSG00000120725.12	SIL1	chr5_138872752_A_G_b38	4.2e-10	0.16	Artery - Tibial
		ENSG00000120725.12	SIL1	chr5_138872752_A_G_b38	4.8e-10	0.17	Adipose - Subcutaneous
		ENSG00000120725.12	SIL1	chr5_138872752_A_G_b38	1.7e-9	0.15	Adipose - Visceral (Omentum)
		ENSG00000120725.12	SIL1	chr5_138872752_A_G_b38	2.6e-9	0.14	Lung
		ENSG00000044115.20	CTNNA1	chr5_138872752_A_G_b38	3.0e-9	-0.11	Nerve - Tibial
		ENSG00000120725.12	SIL1	chr5_138872752_A_G_b38	3.4e-9	0.30	Esophagus - Gastroesophageal Junction
		ENSG00000044115.20	CTNNA1	chr5_138872752_A_G_b38	6.8e-9	-0.13	Cells - Cultured fibroblasts
		ENSG00000120725.12	SIL1	chr5_138872752_A_G_b38	7.5e-9	0.43	Brain - Frontal Cortex (BA9)
		ENSG00000044115.20	CTNNA1	chr5_138872752_A_G_b38	6.1e-8	-0.15	Breast - Mammary Tissue
		ENSG00000120725.12	SIL1	chr5_138872752_A_G_b38	6.6e-8	0.26	Pituitary
		ENSG00000120725.12	SIL1	chr5_138872752_A_G_b38	7.5e-8	0.12	Cells - Cultured fibroblasts
		ENSG00000044115.20	CTNNA1	chr5_138872752_A_G_b38	1.6e-7	-0.15	Colon - Transverse
		ENSG00000044115.20	CTNNA1	chr5_138872752_A_G_b38	3.3e-7	0.10	Testis
		ENSG00000120725.12	SIL1	chr5_138872752_A_G_b38	3.5e-7	0.29	Brain - Cerebellum
		ENSG00000120725.12	SIL1	chr5_138872752_A_G_b38	3.9e-7	0.31	Brain - Cortex
		ENSG00000120725.12	SIL1	chr5_138872752_A_G_b38	4.2e-7	0.33	Brain - Caudate (basal ganglia)
		ENSG00000044115.20	CTNNA1	chr5_138872752_A_G_b38	4.6e-7	-0.098	Skin - Not Sun Exposed (Suprapubic)
		ENSG00000044115.20	CTNNA1	chr5_138872752_A_G_b38	4.9e-7	-0.095	Thyroid
		ENSG00000120725.12	SIL1	chr5_138872752_A_G_b38	7.5e-7	0.15	Esophagus - Mucosa
		ENSG00000044115.20	CTNNA1	chr5_138872752_A_G_b38	0.0000013	-0.21	Brain - Cerebellum
		ENSG00000044115.20	CTNNA1	chr5_138872752_A_G_b38	0.0000018	-0.13	Heart - Atrial Appendage
		ENSG00000120725.12	SIL1	chr5_138872752_A_G_b38	0.0000024	0.34	Brain - Nucleus accumbens (basal ganglia)
		ENSG00000120725.12	SIL1	chr5_138872752_A_G_b38	0.0000025	0.22	Adrenal Gland
		ENSG00000044115.20	CTNNA1	chr5_138872752_A_G_b38	0.0000029	-0.14	Muscle - Skeletal
		ENSG00000120725.12	SIL1	chr5_138872752_A_G_b38	0.0000034	0.41	Brain - Substantia nigra
		ENSG00000044115.20	CTNNA1	chr5_138872752_A_G_b38	0.0000040	-0.083	Skin - Sun Exposed (Lower leg)
		ENSG00000044115.20	CTNNA1	chr5_138872752_A_G_b38	0.0000044	-0.14	Spleen
		ENSG00000120725.12	SIL1	chr5_138872752_A_G_b38	0.000010	0.17	Colon - Transverse
		ENSG00000120725.12	SIL1	chr5_138872752_A_G_b38	0.000014	0.13	Breast - Mammary Tissue
		ENSG00000120725.12	SIL1	chr5_138872752_A_G_b38	0.000023	0.13	Heart - Left Ventricle
		ENSG00000120725.12	SIL1	chr5_138872752_A_G_b38	0.000024	0.32	Brain - Anterior cingulate cortex (BA24)
		ENSG00000044115.20	CTNNA1	chr5_138872752_A_G_b38	0.000025	-0.20	Brain - Cerebellar Hemisphere
		ENSG00000120725.12	SIL1	chr5_138872752_A_G_b38	0.000030	0.26	Artery - Coronary
		ENSG00000044115.20	CTNNA1	chr5_138872752_A_G_b38	0.000039	-0.12	Pancreas
		ENSG00000044115.20	CTNNA1	chr5_138872752_A_G_b38	0.000057	-0.10	Adipose - Subcutaneous
		ENSG00000120725.12	SIL1	chr5_138872752_A_G_b38	0.000069	0.15	Pancreas
		ENSG00000120725.12	SIL1	chr5_138872752_A_G_b38	0.000075	0.26	Brain - Putamen (basal ganglia)
	rs288036	ENSG00000253404.1	AC034243.1	chr5_138875206_C_A_b38	4.5e-131	1.2	Thyroid
		ENSG00000253404.1	AC034243.1	chr5_138875206_C_A_b38	8.5e-93	1.1	Skin - Sun Exposed (Lower leg)
		ENSG00000253404.1	AC034243.1	chr5_138875206_C_A_b38	6.0e-86	1.0	Adipose - Subcutaneous
		ENSG00000253404.1	AC034243.1	chr5_138875206_C_A_b38	3.0e-74	1.1	Esophagus - Mucosa
		ENSG00000044115.20	CTNNA1	chr5_138875206_C_A_b38	7.6e-74	-0.49	Whole Blood
		ENSG00000253404.1	AC034243.1	chr5_138875206_C_A_b38	3.7e-70	1.0	Skin - Not Sun Exposed (Suprapubic)
		ENSG00000253404.1	AC034243.1	chr5_138875206_C_A_b38	1.3e-69	0.94	Nerve - Tibial
		ENSG00000253404.1	AC034243.1	chr5_138875206_C_A_b38	2.8e-65	1.2	Pituitary
		ENSG00000253404.1	AC034243.1	chr5_138875206_C_A_b38	3.3e-65	0.92	Lung
		ENSG00000253404.1	AC034243.1	chr5_138875206_C_A_b38	1.3e-63	1.0	Breast - Mammary Tissue
		ENSG00000253404.1	AC034243.1	chr5_138875206_C_A_b38	3.3e-57	0.92	Adipose - Visceral (Omentum)
		ENSG00000253404.1	AC034243.1	chr5_138875206_C_A_b38	3.7e-57	0.92	Esophagus - Muscularis
		ENSG00000253404.1	AC034243.1	chr5_138875206_C_A_b38	8.0e-56	1.2	Pancreas
		ENSG00000253404.1	AC034243.1	chr5_138875206_C_A_b38	2.2e-55	1.0	Heart - Atrial Appendage
		ENSG00000253404.1	AC034243.1	chr5_138875206_C_A_b38	2.7e-54	0.95	Stomach
		ENSG00000253404.1	AC034243.1	chr5_138875206_C_A_b38	1.4e-49	0.95	Heart - Left Ventricle
		ENSG00000253404.1	AC034243.1	chr5_138875206_C_A_b38	3.4e-48	1.2	Adrenal Gland
		ENSG00000253404.1	AC034243.1	chr5_138875206_C_A_b38	2.1e-46	0.77	Cells - Cultured fibroblasts
		ENSG00000253404.1	AC034243.1	chr5_138875206_C_A_b38	6.3e-42	0.89	Colon - Transverse
		ENSG00000044115.20	CTNNA1	chr5_138875206_C_A_b38	2.2e-40	-0.27	Esophagus - Mucosa
		ENSG00000253404.1	AC034243.1	chr5_138875206_C_A_b38	4.6e-39	0.93	Prostate
		ENSG00000253404.1	AC034243.1	chr5_138875206_C_A_b38	5.0e-34	0.88	Esophagus - Gastroesophageal Junction
		ENSG00000253404.1	AC034243.1	chr5_138875206_C_A_b38	1.1e-30	0.88	Colon - Sigmoid
		ENSG00000253404.1	AC034243.1	chr5_138875206_C_A_b38	8.0e-29	1.0	Spleen
		ENSG00000253404.1	AC034243.1	chr5_138875206_C_A_b38	1.0e-28	1.0	Brain - Putamen (basal ganglia)
		ENSG00000253404.1	AC034243.1	chr5_138875206_C_A_b38	1.2e-28	1.1	Brain - Caudate (basal ganglia)
		ENSG00000253404.1	AC034243.1	chr5_138875206_C_A_b38	1.7e-26	0.95	Liver
		ENSG00000253404.1	AC034243.1	chr5_138875206_C_A_b38	6.1e-26	1.1	Ovary
		ENSG00000253404.1	AC034243.1	chr5_138875206_C_A_b38	1.2e-25	1.0	Brain - Nucleus accumbens (basal ganglia)
		ENSG00000253404.1	AC034243.1	chr5_138875206_C_A_b38	8.6e-24	0.49	Testis
		ENSG00000253404.1	AC034243.1	chr5_138875206_C_A_b38	9.2e-23	0.87	Brain - Cortex
		ENSG00000253404.1	AC034243.1	chr5_138875206_C_A_b38	2.5e-22	1.1	Minor Salivary Gland
		ENSG00000253404.1	AC034243.1	chr5_138875206_C_A_b38	1.5e-21	0.87	Artery - Coronary
		ENSG00000253404.1	AC034243.1	chr5_138875206_C_A_b38	7.6e-20	1.0	Brain - Spinal cord (cervical c-1)
		ENSG00000253404.1	AC034243.1	chr5_138875206_C_A_b38	3.2e-19	0.94	Brain - Hippocampus
		ENSG00000253404.1	AC034243.1	chr5_138875206_C_A_b38	1.7e-18	0.99	Brain - Substantia nigra
		ENSG00000253404.1	AC034243.1	chr5_138875206_C_A_b38	3.4e-18	0.90	Brain - Hypothalamus
		ENSG00000120725.12	SIL1	chr5_138875206_C_A_b38	3.7e-18	0.33	Esophagus - Muscularis
		ENSG00000120725.12	SIL1	chr5_138875206_C_A_b38	9.7e-18	0.21	Nerve - Tibial
		ENSG00000253404.1	AC034243.1	chr5_138875206_C_A_b38	3.7e-17	1.1	Uterus
		ENSG00000253404.1	AC034243.1	chr5_138875206_C_A_b38	5.7e-17	1.1	Brain - Amygdala
		ENSG00000253404.1	AC034243.1	chr5_138875206_C_A_b38	5.8e-17	0.79	Brain - Frontal Cortex (BA9)
		ENSG00000120725.12	SIL1	chr5_138875206_C_A_b38	6.4e-17	0.22	Skin - Sun Exposed (Lower leg)
		ENSG00000253404.1	AC034243.1	chr5_138875206_C_A_b38	1.9e-16	0.80	Small Intestine - Terminal Ileum
		ENSG00000253404.1	AC034243.1	chr5_138875206_C_A_b38	4.5e-16	1.1	Vagina

		ENSG00000253404.1	AC034243.1	chr5_138875206_C_A_b38	5.3e-16	0.79	Brain - Anterior cingulate cortex (BA24)
		ENSG00000120725.12	SIL1	chr5_138875206_C_A_b38	1.3e-15	0.22	Skin - Not Sun Exposed (Suprapubic)
		ENSG00000120725.12	SIL1	chr5_138875206_C_A_b38	2.2e-15	0.29	Artery - Aorta
		ENSG00000120725.12	SIL1	chr5_138875206_C_A_b38	4.1e-14	0.29	Stomach
		ENSG00000253404.1	AC034243.1	chr5_138875206_C_A_b38	1.4e-12	1.1	Kidney - Cortex
		ENSG00000120725.12	SIL1	chr5_138875206_C_A_b38	3.9e-12	0.14	Thyroid
		ENSG00000120725.12	SIL1	chr5_138875206_C_A_b38	4.2e-10	0.16	Artery - Tibial
		ENSG00000120725.12	SIL1	chr5_138875206_C_A_b38	4.8e-10	0.17	Adipose - Subcutaneous
		ENSG00000120725.12	SIL1	chr5_138875206_C_A_b38	1.7e-9	0.15	Adipose - Visceral (Omentum)
		ENSG00000120725.12	SIL1	chr5_138875206_C_A_b38	2.6e-9	0.14	Lung
		ENSG00000044115.20	CTNNA1	chr5_138875206_C_A_b38	3.0e-9	-0.11	Nerve - Tibial
		ENSG00000120725.12	SIL1	chr5_138875206_C_A_b38	3.4e-9	0.30	Esophagus - Gastroesophageal Junction
		ENSG00000044115.20	CTNNA1	chr5_138875206_C_A_b38	6.8e-9	-0.13	Cells - Cultured fibroblasts
		ENSG00000120725.12	SIL1	chr5_138875206_C_A_b38	7.5e-9	0.43	Brain - Frontal Cortex (BA9)
		ENSG00000044115.20	CTNNA1	chr5_138875206_C_A_b38	6.1e-8	-0.15	Breast - Mammary Tissue
		ENSG00000120725.12	SIL1	chr5_138875206_C_A_b38	6.6e-8	0.26	Pituitary
		ENSG00000120725.12	SIL1	chr5_138875206_C_A_b38	7.5e-8	0.12	Cells - Cultured fibroblasts
		ENSG00000044115.20	CTNNA1	chr5_138875206_C_A_b38	1.6e-7	-0.15	Colon - Transverse
		ENSG00000044115.20	CTNNA1	chr5_138875206_C_A_b38	3.3e-7	0.10	Testis
		ENSG00000120725.12	SIL1	chr5_138875206_C_A_b38	3.5e-7	0.29	Brain - Cerebellum
		ENSG00000120725.12	SIL1	chr5_138875206_C_A_b38	3.9e-7	0.31	Brain - Cortex
		ENSG00000120725.12	SIL1	chr5_138875206_C_A_b38	4.2e-7	0.33	Brain - Caudate (basal ganglia)
		ENSG00000044115.20	CTNNA1	chr5_138875206_C_A_b38	4.6e-7	-0.098	Skin - Not Sun Exposed (Suprapubic)
		ENSG00000044115.20	CTNNA1	chr5_138875206_C_A_b38	4.9e-7	-0.095	Thyroid
		ENSG00000120725.12	SIL1	chr5_138875206_C_A_b38	7.5e-7	0.15	Esophagus - Mucosa
		ENSG00000044115.20	CTNNA1	chr5_138875206_C_A_b38	0.0000013	-0.21	Brain - Cerebellum
		ENSG00000044115.20	CTNNA1	chr5_138875206_C_A_b38	0.0000018	-0.13	Heart - Atrial Appendage
		ENSG00000120725.12	SIL1	chr5_138875206_C_A_b38	0.0000024	0.34	Brain - Nucleus accumbens (basal ganglia)
		ENSG00000120725.12	SIL1	chr5_138875206_C_A_b38	0.0000025	0.22	Adrenal Gland
		ENSG00000044115.20	CTNNA1	chr5_138875206_C_A_b38	0.0000029	-0.14	Muscle - Skeletal
		ENSG00000120725.12	SIL1	chr5_138875206_C_A_b38	0.0000034	0.41	Brain - Substantia nigra
		ENSG00000044115.20	CTNNA1	chr5_138875206_C_A_b38	0.0000040	-0.083	Skin - Sun Exposed (Lower leg)
		ENSG00000044115.20	CTNNA1	chr5_138875206_C_A_b38	0.0000044	-0.14	Spleen
		ENSG00000120725.12	SIL1	chr5_138875206_C_A_b38	0.000010	0.17	Colon - Transverse
		ENSG00000120725.12	SIL1	chr5_138875206_C_A_b38	0.000014	0.13	Breast - Mammary Tissue
		ENSG00000120725.12	SIL1	chr5_138875206_C_A_b38	0.000023	0.13	Heart - Left Ventricle
		ENSG00000120725.12	SIL1	chr5_138875206_C_A_b38	0.000024	0.32	Brain - Anterior cingulate cortex (BA24)
		ENSG00000044115.20	CTNNA1	chr5_138875206_C_A_b38	0.000025	-0.20	Brain - Cerebellar Hemisphere
		ENSG00000120725.12	SIL1	chr5_138875206_C_A_b38	0.000030	0.26	Artery - Coronary
		ENSG00000044115.20	CTNNA1	chr5_138875206_C_A_b38	0.000039	-0.12	Pancreas
		ENSG00000044115.20	CTNNA1	chr5_138875206_C_A_b38	0.000057	-0.10	Adipose - Subcutaneous
		ENSG00000120725.12	SIL1	chr5_138875206_C_A_b38	0.000069	0.15	Pancreas
		ENSG00000120725.12	SIL1	chr5_138875206_C_A_b38	0.000075	0.26	Brain - Putamen (basal ganglia)
	rs73790375	ENSG00000253404.1	AC034243.1	chr5_138876605_C_G_b38	1.3e-9	1.7	Skin - Not Sun Exposed (Suprapubic)
		ENSG00000253404.1	AC034243.1	chr5_138876605_C_G_b38	2.8e-8	1.6	Skin - Sun Exposed (Lower leg)
		ENSG00000253404.1	AC034243.1	chr5_138876605_C_G_b38	7.0e-8	2.0	Heart - Atrial Appendage
		ENSG00000253404.1	AC034243.1	chr5_138876605_C_G_b38	8.8e-7	1.3	Adipose - Subcutaneous
		ENSG00000253404.1	AC034243.1	chr5_138876605_C_G_b38	0.0000012	1.9	Heart - Left Ventricle
		ENSG00000253404.1	AC034243.1	chr5_138876605_C_G_b38	0.0000014	1.3	Lung
		ENSG00000253404.1	AC034243.1	chr5_138876605_C_G_b38	0.0000046	1.7	Esophagus - Gastroesophageal Junction
		ENSG00000253404.1	AC034243.1	chr5_138876605_C_G_b38	0.0000048	1.3	Thyroid
		ENSG00000253404.1	AC034243.1	chr5_138876605_C_G_b38	0.0000089	1.6	Colon - Sigmoid
		ENSG00000253404.1	AC034243.1	chr5_138876605_C_G_b38	0.000015	1.4	Colon - Transverse
		ENSG00000253404.1	AC034243.1	chr5_138876605_C_G_b38	0.000019	1.5	Breast - Mammary Tissue
		ENSG00000253404.1	AC034243.1	chr5_138876605_C_G_b38	0.000019	1.2	Adipose - Visceral (Omentum)
		ENSG00000253404.1	AC034243.1	chr5_138876605_C_G_b38	0.000028	1.5	Esophagus - Mucosa
		ENSG00000015479.18	MATR3	chr5_138876605_C_G_b38	0.000052	-1.1	Prostate
		ENSG00000253404.1	AC034243.1	chr5_138876605_C_G_b38	0.000056	1.1	Nerve - Tibial
	rs288035	ENSG00000253404.1	AC034243.1	chr5_138876728_A_T_b38	4.5e-131	1.2	Thyroid
		ENSG00000253404.1	AC034243.1	chr5_138876728_A_T_b38	8.5e-93	1.1	Skin - Sun Exposed (Lower leg)
		ENSG00000253404.1	AC034243.1	chr5_138876728_A_T_b38	6.0e-86	1.0	Adipose - Subcutaneous
		ENSG00000253404.1	AC034243.1	chr5_138876728_A_T_b38	3.0e-74	1.1	Esophagus - Mucosa
		ENSG00000044115.20	CTNNA1	chr5_138876728_A_T_b38	7.6e-74	-0.49	Whole Blood
		ENSG00000253404.1	AC034243.1	chr5_138876728_A_T_b38	3.7e-70	1.0	Skin - Not Sun Exposed (Suprapubic)
		ENSG00000253404.1	AC034243.1	chr5_138876728_A_T_b38	1.3e-69	0.94	Nerve - Tibial
		ENSG00000253404.1	AC034243.1	chr5_138876728_A_T_b38	2.8e-65	1.2	Pituitary
		ENSG00000253404.1	AC034243.1	chr5_138876728_A_T_b38	3.3e-65	0.92	Lung
		ENSG00000253404.1	AC034243.1	chr5_138876728_A_T_b38	1.3e-63	1.0	Breast - Mammary Tissue
		ENSG00000253404.1	AC034243.1	chr5_138876728_A_T_b38	3.3e-57	0.92	Adipose - Visceral (Omentum)
		ENSG00000253404.1	AC034243.1	chr5_138876728_A_T_b38	3.7e-57	0.92	Esophagus - Muscularis
		ENSG00000253404.1	AC034243.1	chr5_138876728_A_T_b38	8.0e-56	1.2	Pancreas
		ENSG00000253404.1	AC034243.1	chr5_138876728_A_T_b38	2.2e-55	1.0	Heart - Atrial Appendage
		ENSG00000253404.1	AC034243.1	chr5_138876728_A_T_b38	2.7e-54	0.95	Stomach
		ENSG00000253404.1	AC034243.1	chr5_138876728_A_T_b38	1.4e-49	0.95	Heart - Left Ventricle
		ENSG00000253404.1	AC034243.1	chr5_138876728_A_T_b38	3.4e-48	1.2	Adrenal Gland
		ENSG00000253404.1	AC034243.1	chr5_138876728_A_T_b38	2.1e-46	0.77	Cells - Cultured fibroblasts
		ENSG00000253404.1	AC034243.1	chr5_138876728_A_T_b38	6.3e-42	0.89	Colon - Transverse
		ENSG00000044115.20	CTNNA1	chr5_138876728_A_T_b38	2.2e-40	-0.27	Esophagus - Mucosa
		ENSG00000253404.1	AC034243.1	chr5_138876728_A_T_b38	4.6e-39	0.93	Prostate
		ENSG00000253404.1	AC034243.1	chr5_138876728_A_T_b38	5.0e-34	0.88	Esophagus - Gastroesophageal Junction
		ENSG00000253404.1	AC034243.1	chr5_138876728_A_T_b38	1.1e-30	0.88	Colon - Sigmoid
		ENSG00000253404.1	AC034243.1	chr5_138876728_A_T_b38	8.0e-29	1.0	Spleen

		ENSG00000253404.1	AC034243.1	chr5_138876728_A_T_b38	1.0e-28	1.0	Brain - Putamen (basal ganglia)
		ENSG00000253404.1	AC034243.1	chr5_138876728_A_T_b38	1.2e-28	1.1	Brain - Caudate (basal ganglia)
		ENSG00000253404.1	AC034243.1	chr5_138876728_A_T_b38	1.7e-26	0.95	Liver
		ENSG00000253404.1	AC034243.1	chr5_138876728_A_T_b38	6.1e-26	1.1	Ovary
		ENSG00000253404.1	AC034243.1	chr5_138876728_A_T_b38	1.2e-25	1.0	Brain - Nucleus accumbens (basal ganglia)
		ENSG00000253404.1	AC034243.1	chr5_138876728_A_T_b38	8.6e-24	0.49	Testis
		ENSG00000253404.1	AC034243.1	chr5_138876728_A_T_b38	9.2e-23	0.87	Brain - Cortex
		ENSG00000253404.1	AC034243.1	chr5_138876728_A_T_b38	2.5e-22	1.1	Minor Salivary Gland
		ENSG00000253404.1	AC034243.1	chr5_138876728_A_T_b38	1.5e-21	0.87	Artery - Coronary
		ENSG00000253404.1	AC034243.1	chr5_138876728_A_T_b38	7.6e-20	1.0	Brain - Spinal cord (cervical c-1)
		ENSG00000253404.1	AC034243.1	chr5_138876728_A_T_b38	3.2e-19	0.94	Brain - Hippocampus
		ENSG00000253404.1	AC034243.1	chr5_138876728_A_T_b38	1.7e-18	0.99	Brain - Substantia nigra
		ENSG00000253404.1	AC034243.1	chr5_138876728_A_T_b38	3.4e-18	0.90	Brain - Hypothalamus
		ENSG00000120725.12	SIL1	chr5_138876728_A_T_b38	3.7e-18	0.33	Esophagus - Muscularis
		ENSG00000120725.12	SIL1	chr5_138876728_A_T_b38	9.7e-18	0.21	Nerve - Tibial
		ENSG00000253404.1	AC034243.1	chr5_138876728_A_T_b38	3.7e-17	1.1	Uterus
		ENSG00000253404.1	AC034243.1	chr5_138876728_A_T_b38	5.7e-17	1.1	Brain - Amygdala
		ENSG00000253404.1	AC034243.1	chr5_138876728_A_T_b38	5.8e-17	0.79	Brain - Frontal Cortex (BA9)
		ENSG00000120725.12	SIL1	chr5_138876728_A_T_b38	6.4e-17	0.22	Skin - Sun Exposed (Lower leg)
		ENSG00000253404.1	AC034243.1	chr5_138876728_A_T_b38	1.9e-16	0.80	Small Intestine - Terminal Ileum
		ENSG00000253404.1	AC034243.1	chr5_138876728_A_T_b38	4.5e-16	1.1	Vagina
		ENSG00000253404.1	AC034243.1	chr5_138876728_A_T_b38	5.3e-16	0.79	Brain - Anterior cingulate cortex (BA24)
		ENSG00000120725.12	SIL1	chr5_138876728_A_T_b38	1.3e-15	0.22	Skin - Not Sun Exposed (Suprapubic)
		ENSG00000120725.12	SIL1	chr5_138876728_A_T_b38	2.2e-15	0.29	Artery - Aorta
		ENSG00000120725.12	SIL1	chr5_138876728_A_T_b38	4.1e-14	0.29	Stomach
		ENSG00000253404.1	AC034243.1	chr5_138876728_A_T_b38	1.4e-12	1.1	Kidney - Cortex
		ENSG00000120725.12	SIL1	chr5_138876728_A_T_b38	3.9e-12	0.14	Thyroid
		ENSG00000120725.12	SIL1	chr5_138876728_A_T_b38	4.2e-10	0.16	Artery - Tibial
		ENSG00000120725.12	SIL1	chr5_138876728_A_T_b38	4.8e-10	0.17	Adipose - Subcutaneous
		ENSG00000120725.12	SIL1	chr5_138876728_A_T_b38	1.7e-9	0.15	Adipose - Visceral (Omentum)
		ENSG00000120725.12	SIL1	chr5_138876728_A_T_b38	2.6e-9	0.14	Lung
		ENSG00000044115.20	CTNNA1	chr5_138876728_A_T_b38	3.0e-9	-0.11	Nerve - Tibial
		ENSG00000120725.12	SIL1	chr5_138876728_A_T_b38	3.4e-9	0.30	Esophagus - Gastroesophageal Junction
		ENSG00000044115.20	CTNNA1	chr5_138876728_A_T_b38	6.8e-9	-0.13	Cells - Cultured fibroblasts
		ENSG00000120725.12	SIL1	chr5_138876728_A_T_b38	7.5e-9	0.43	Brain - Frontal Cortex (BA9)
		ENSG00000044115.20	CTNNA1	chr5_138876728_A_T_b38	6.1e-8	-0.15	Breast - Mammary Tissue
		ENSG00000120725.12	SIL1	chr5_138876728_A_T_b38	6.6e-8	0.26	Pituitary
		ENSG00000120725.12	SIL1	chr5_138876728_A_T_b38	7.5e-8	0.12	Cells - Cultured fibroblasts
		ENSG00000044115.20	CTNNA1	chr5_138876728_A_T_b38	1.6e-7	-0.15	Colon - Transverse
		ENSG00000044115.20	CTNNA1	chr5_138876728_A_T_b38	3.3e-7	0.10	Testis
		ENSG00000120725.12	SIL1	chr5_138876728_A_T_b38	3.5e-7	0.29	Brain - Cerebellum
		ENSG00000120725.12	SIL1	chr5_138876728_A_T_b38	3.9e-7	0.31	Brain - Cortex
		ENSG00000120725.12	SIL1	chr5_138876728_A_T_b38	4.2e-7	0.33	Brain - Caudate (basal ganglia)
		ENSG00000044115.20	CTNNA1	chr5_138876728_A_T_b38	4.6e-7	-0.098	Skin - Not Sun Exposed (Suprapubic)
		ENSG00000044115.20	CTNNA1	chr5_138876728_A_T_b38	4.9e-7	-0.095	Thyroid
		ENSG00000120725.12	SIL1	chr5_138876728_A_T_b38	7.5e-7	0.15	Esophagus - Mucosa
		ENSG00000044115.20	CTNNA1	chr5_138876728_A_T_b38	0.0000013	-0.21	Brain - Cerebellum
		ENSG00000044115.20	CTNNA1	chr5_138876728_A_T_b38	0.0000018	-0.13	Heart - Atrial Appendage
		ENSG00000120725.12	SIL1	chr5_138876728_A_T_b38	0.0000024	0.34	Brain - Nucleus accumbens (basal ganglia)
		ENSG00000120725.12	SIL1	chr5_138876728_A_T_b38	0.0000025	0.22	Adrenal Gland
		ENSG00000044115.20	CTNNA1	chr5_138876728_A_T_b38	0.0000029	-0.14	Muscle - Skeletal
		ENSG00000120725.12	SIL1	chr5_138876728_A_T_b38	0.0000034	0.41	Brain - Substantia nigra
		ENSG00000044115.20	CTNNA1	chr5_138876728_A_T_b38	0.0000040	-0.083	Skin - Sun Exposed (Lower leg)
		ENSG00000044115.20	CTNNA1	chr5_138876728_A_T_b38	0.0000044	-0.14	Spleen
		ENSG00000120725.12	SIL1	chr5_138876728_A_T_b38	0.000010	0.17	Colon - Transverse
		ENSG00000120725.12	SIL1	chr5_138876728_A_T_b38	0.000014	0.13	Breast - Mammary Tissue
		ENSG00000120725.12	SIL1	chr5_138876728_A_T_b38	0.000023	0.13	Heart - Left Ventricle
		ENSG00000120725.12	SIL1	chr5_138876728_A_T_b38	0.000024	0.32	Brain - Anterior cingulate cortex (BA24)
		ENSG00000044115.20	CTNNA1	chr5_138876728_A_T_b38	0.000025	-0.20	Brain - Cerebellar Hemisphere
		ENSG00000120725.12	SIL1	chr5_138876728_A_T_b38	0.000030	0.26	Artery - Coronary
		ENSG00000044115.20	CTNNA1	chr5_138876728_A_T_b38	0.000039	-0.12	Pancreas
		ENSG00000044115.20	CTNNA1	chr5_138876728_A_T_b38	0.000057	-0.10	Adipose - Subcutaneous
		ENSG00000120725.12	SIL1	chr5_138876728_A_T_b38	0.000069	0.15	Pancreas
		ENSG00000120725.12	SIL1	chr5_138876728_A_T_b38	0.000075	0.26	Brain - Putamen (basal ganglia)
	rs288032	ENSG00000253404.1	AC034243.1	chr5_138879639_G_A_b38	4.5e-131	1.2	Thyroid
		ENSG00000253404.1	AC034243.1	chr5_138879639_G_A_b38	8.5e-93	1.1	Skin - Sun Exposed (Lower leg)
		ENSG00000253404.1	AC034243.1	chr5_138879639_G_A_b38	6.0e-86	1.0	Adipose - Subcutaneous
		ENSG00000253404.1	AC034243.1	chr5_138879639_G_A_b38	3.0e-74	1.1	Esophagus - Mucosa
		ENSG00000044115.20	CTNNA1	chr5_138879639_G_A_b38	7.6e-74	-0.49	Whole Blood
		ENSG00000253404.1	AC034243.1	chr5_138879639_G_A_b38	3.7e-70	1.0	Skin - Not Sun Exposed (Suprapubic)
		ENSG00000253404.1	AC034243.1	chr5_138879639_G_A_b38	1.3e-69	0.94	Nerve - Tibial
		ENSG00000253404.1	AC034243.1	chr5_138879639_G_A_b38	2.8e-65	1.2	Pituitary
		ENSG00000253404.1	AC034243.1	chr5_138879639_G_A_b38	3.3e-65	0.92	Lung
		ENSG00000253404.1	AC034243.1	chr5_138879639_G_A_b38	1.3e-63	1.0	Breast - Mammary Tissue
		ENSG00000253404.1	AC034243.1	chr5_138879639_G_A_b38	3.3e-57	0.92	Adipose - Visceral (Omentum)
		ENSG00000253404.1	AC034243.1	chr5_138879639_G_A_b38	3.7e-57	0.92	Esophagus - Muscularis
		ENSG00000253404.1	AC034243.1	chr5_138879639_G_A_b38	8.0e-56	1.2	Pancreas
		ENSG00000253404.1	AC034243.1	chr5_138879639_G_A_b38	2.2e-55	1.0	Heart - Atrial Appendage
		ENSG00000253404.1	AC034243.1	chr5_138879639_G_A_b38	2.7e-54	0.95	Stomach
		ENSG00000253404.1	AC034243.1	chr5_138879639_G_A_b38	1.4e-49	0.95	Heart - Left Ventricle
		ENSG00000253404.1	AC034243.1	chr5_138879639_G_A_b38	3.4e-48	1.2	Adrenal Gland
		ENSG00000253404.1	AC034243.1	chr5_138879639_G_A_b38	2.1e-46	0.77	Cells - Cultured fibroblasts

		ENSG00000253404.1	AC034243.1	chr5_138879639_G_A_b38	6.3e-42	0.89	Colon - Transverse
		ENSG00000044115.20	CTNNA1	chr5_138879639_G_A_b38	2.2e-40	-0.27	Esophagus - Mucosa
		ENSG00000253404.1	AC034243.1	chr5_138879639_G_A_b38	4.6e-39	0.93	Prostate
		ENSG00000253404.1	AC034243.1	chr5_138879639_G_A_b38	5.0e-34	0.88	Esophagus - Gastroesophageal Junction
		ENSG00000253404.1	AC034243.1	chr5_138879639_G_A_b38	1.1e-30	0.88	Colon - Sigmoid
		ENSG00000253404.1	AC034243.1	chr5_138879639_G_A_b38	8.0e-29	1.0	Spleen
		ENSG00000253404.1	AC034243.1	chr5_138879639_G_A_b38	1.0e-28	1.0	Brain - Putamen (basal ganglia)
		ENSG00000253404.1	AC034243.1	chr5_138879639_G_A_b38	1.2e-28	1.1	Brain - Caudate (basal ganglia)
		ENSG00000253404.1	AC034243.1	chr5_138879639_G_A_b38	1.7e-26	0.95	Liver
		ENSG00000253404.1	AC034243.1	chr5_138879639_G_A_b38	6.1e-26	1.1	Ovary
		ENSG00000253404.1	AC034243.1	chr5_138879639_G_A_b38	1.2e-25	1.0	Brain - Nucleus accumbens (basal ganglia)
		ENSG00000253404.1	AC034243.1	chr5_138879639_G_A_b38	8.6e-24	0.49	Testis
		ENSG00000253404.1	AC034243.1	chr5_138879639_G_A_b38	9.2e-23	0.87	Brain - Cortex
		ENSG00000253404.1	AC034243.1	chr5_138879639_G_A_b38	2.5e-22	1.1	Minor Salivary Gland
		ENSG00000253404.1	AC034243.1	chr5_138879639_G_A_b38	1.5e-21	0.87	Artery - Coronary
		ENSG00000253404.1	AC034243.1	chr5_138879639_G_A_b38	7.6e-20	1.0	Brain - Spinal cord (cervical c-1)
		ENSG00000253404.1	AC034243.1	chr5_138879639_G_A_b38	3.2e-19	0.94	Brain - Hippocampus
		ENSG00000253404.1	AC034243.1	chr5_138879639_G_A_b38	1.7e-18	0.99	Brain - Substantia nigra
		ENSG00000253404.1	AC034243.1	chr5_138879639_G_A_b38	3.4e-18	0.90	Brain - Hypothalamus
		ENSG00000120725.12	SIL1	chr5_138879639_G_A_b38	3.7e-18	0.33	Esophagus - Muscularis
		ENSG00000120725.12	SIL1	chr5_138879639_G_A_b38	9.7e-18	0.21	Nerve - Tibial
		ENSG00000253404.1	AC034243.1	chr5_138879639_G_A_b38	3.7e-17	1.1	Uterus
		ENSG00000253404.1	AC034243.1	chr5_138879639_G_A_b38	5.7e-17	1.1	Brain - Amygdala
		ENSG00000253404.1	AC034243.1	chr5_138879639_G_A_b38	5.8e-17	0.79	Brain - Frontal Cortex (BA9)
		ENSG00000120725.12	SIL1	chr5_138879639_G_A_b38	6.4e-17	0.22	Skin - Sun Exposed (Lower leg)
		ENSG00000253404.1	AC034243.1	chr5_138879639_G_A_b38	1.9e-16	0.80	Small Intestine - Terminal Ileum
		ENSG00000253404.1	AC034243.1	chr5_138879639_G_A_b38	4.5e-16	1.1	Vagina
		ENSG00000253404.1	AC034243.1	chr5_138879639_G_A_b38	5.3e-16	0.79	Brain - Anterior cingulate cortex (BA24)
		ENSG00000120725.12	SIL1	chr5_138879639_G_A_b38	1.3e-15	0.22	Skin - Not Sun Exposed (Suprapubic)
		ENSG00000120725.12	SIL1	chr5_138879639_G_A_b38	2.2e-15	0.29	Artery - Aorta
		ENSG00000120725.12	SIL1	chr5_138879639_G_A_b38	4.1e-14	0.29	Stomach
		ENSG00000253404.1	AC034243.1	chr5_138879639_G_A_b38	1.4e-12	1.1	Kidney - Cortex
		ENSG00000120725.12	SIL1	chr5_138879639_G_A_b38	3.9e-12	0.14	Thyroid
		ENSG00000120725.12	SIL1	chr5_138879639_G_A_b38	4.2e-10	0.16	Artery - Tibial
		ENSG00000120725.12	SIL1	chr5_138879639_G_A_b38	4.8e-10	0.17	Adipose - Subcutaneous
		ENSG00000120725.12	SIL1	chr5_138879639_G_A_b38	1.7e-9	0.15	Adipose - Visceral (Omentum)
		ENSG00000120725.12	SIL1	chr5_138879639_G_A_b38	2.6e-9	0.14	Lung
		ENSG00000044115.20	CTNNA1	chr5_138879639_G_A_b38	3.0e-9	-0.11	Nerve - Tibial
		ENSG00000120725.12	SIL1	chr5_138879639_G_A_b38	3.4e-9	0.30	Esophagus - Gastroesophageal Junction
		ENSG00000044115.20	CTNNA1	chr5_138879639_G_A_b38	6.8e-9	-0.13	Cells - Cultured fibroblasts
		ENSG00000120725.12	SIL1	chr5_138879639_G_A_b38	7.5e-9	0.43	Brain - Frontal Cortex (BA9)
		ENSG00000044115.20	CTNNA1	chr5_138879639_G_A_b38	6.1e-8	-0.15	Breast - Mammary Tissue
		ENSG00000120725.12	SIL1	chr5_138879639_G_A_b38	6.6e-8	0.26	Pituitary
		ENSG00000120725.12	SIL1	chr5_138879639_G_A_b38	7.5e-8	0.12	Cells - Cultured fibroblasts
		ENSG00000044115.20	CTNNA1	chr5_138879639_G_A_b38	1.6e-7	-0.15	Colon - Transverse
		ENSG00000044115.20	CTNNA1	chr5_138879639_G_A_b38	3.3e-7	0.10	Testis
		ENSG00000120725.12	SIL1	chr5_138879639_G_A_b38	3.5e-7	0.29	Brain - Cerebellum
		ENSG00000120725.12	SIL1	chr5_138879639_G_A_b38	3.9e-7	0.31	Brain - Cortex
		ENSG00000120725.12	SIL1	chr5_138879639_G_A_b38	4.2e-7	0.33	Brain - Caudate (basal ganglia)
		ENSG00000044115.20	CTNNA1	chr5_138879639_G_A_b38	4.6e-7	-0.098	Skin - Not Sun Exposed (Suprapubic)
		ENSG00000044115.20	CTNNA1	chr5_138879639_G_A_b38	4.9e-7	-0.095	Thyroid
		ENSG00000120725.12	SIL1	chr5_138879639_G_A_b38	7.5e-7	0.15	Esophagus - Mucosa
		ENSG00000044115.20	CTNNA1	chr5_138879639_G_A_b38	0.0000013	-0.21	Brain - Cerebellum
		ENSG00000044115.20	CTNNA1	chr5_138879639_G_A_b38	0.0000018	-0.13	Heart - Atrial Appendage
		ENSG00000120725.12	SIL1	chr5_138879639_G_A_b38	0.0000024	0.34	Brain - Nucleus accumbens (basal ganglia)
		ENSG00000120725.12	SIL1	chr5_138879639_G_A_b38	0.0000025	0.22	Adrenal Gland
		ENSG00000044115.20	CTNNA1	chr5_138879639_G_A_b38	0.0000029	-0.14	Muscle - Skeletal
		ENSG00000120725.12	SIL1	chr5_138879639_G_A_b38	0.0000034	0.41	Brain - Substantia nigra
		ENSG00000044115.20	CTNNA1	chr5_138879639_G_A_b38	0.0000040	-0.083	Skin - Sun Exposed (Lower leg)
		ENSG00000044115.20	CTNNA1	chr5_138879639_G_A_b38	0.0000044	-0.14	Spleen
		ENSG00000120725.12	SIL1	chr5_138879639_G_A_b38	0.000010	0.17	Colon - Transverse
		ENSG00000120725.12	SIL1	chr5_138879639_G_A_b38	0.000014	0.13	Breast - Mammary Tissue
		ENSG00000120725.12	SIL1	chr5_138879639_G_A_b38	0.000023	0.13	Heart - Left Ventricle
		ENSG00000120725.12	SIL1	chr5_138879639_G_A_b38	0.000024	0.32	Brain - Anterior cingulate cortex (BA24)
		ENSG00000044115.20	CTNNA1	chr5_138879639_G_A_b38	0.000025	-0.20	Brain - Cerebellar Hemisphere
		ENSG00000120725.12	SIL1	chr5_138879639_G_A_b38	0.000030	0.26	Artery - Coronary
		ENSG00000044115.20	CTNNA1	chr5_138879639_G_A_b38	0.000039	-0.12	Pancreas
		ENSG00000044115.20	CTNNA1	chr5_138879639_G_A_b38	0.000057	-0.10	Adipose - Subcutaneous
		ENSG00000120725.12	SIL1	chr5_138879639_G_A_b38	0.000069	0.15	Pancreas
		ENSG00000120725.12	SIL1	chr5_138879639_G_A_b38	0.000075	0.26	Brain - Putamen (basal ganglia)
	rs57948908	ENSG00000253404.1	AC034243.1	chr5_138880221_T_C_b38	1.3e-9	1.7	Skin - Not Sun Exposed (Suprapubic)
		ENSG00000253404.1	AC034243.1	chr5_138880221_T_C_b38	2.8e-8	1.6	Skin - Sun Exposed (Lower leg)
		ENSG00000253404.1	AC034243.1	chr5_138880221_T_C_b38	7.0e-8	2.0	Heart - Atrial Appendage
		ENSG00000253404.1	AC034243.1	chr5_138880221_T_C_b38	8.8e-7	1.3	Adipose - Subcutaneous
		ENSG00000253404.1	AC034243.1	chr5_138880221_T_C_b38	0.0000012	1.9	Heart - Left Ventricle
		ENSG00000253404.1	AC034243.1	chr5_138880221_T_C_b38	0.0000014	1.3	Lung
		ENSG00000253404.1	AC034243.1	chr5_138880221_T_C_b38	0.0000046	1.7	Esophagus - Gastroesophageal Junction
		ENSG00000253404.1	AC034243.1	chr5_138880221_T_C_b38	0.0000048	1.3	Thyroid
		ENSG00000253404.1	AC034243.1	chr5_138880221_T_C_b38	0.0000089	1.6	Colon - Sigmoid
		ENSG00000253404.1	AC034243.1	chr5_138880221_T_C_b38	0.000015	1.4	Colon - Transverse
		ENSG00000253404.1	AC034243.1	chr5_138880221_T_C_b38	0.000019	1.5	Breast - Mammary Tissue
		ENSG00000253404.1	AC034243.1	chr5_138880221_T_C_b38	0.000019	1.2	Adipose - Visceral (Omentum)

		ENSG00000253404.1	AC034243.1	chr5_138880221_T_C_b38	0.000028	1.5	Esophagus - Mucosa
		ENSG00000015479.18	MATR3	chr5_138880221_T_C_b38	0.000052	-1.1	Prostate
		ENSG00000253404.1	AC034243.1	chr5_138880221_T_C_b38	0.000056	1.1	Nerve - Tibial
		ENSG00000253404.1	AC034243.1	chr5_138881616_T_C_b38	4.0e-128	1.2	Thyroid
		ENSG00000253404.1	AC034243.1	chr5_138881616_T_C_b38	3.2e-90	1.1	Skin - Sun Exposed (Lower leg)
		ENSG00000253404.1	AC034243.1	chr5_138881616_T_C_b38	1.6e-83	1.0	Adipose - Subcutaneous
		ENSG00000253404.1	AC034243.1	chr5_138881616_T_C_b38	7.1e-74	1.1	Esophagus - Mucosa
		ENSG00000044115.20	CTNNA1	chr5_138881616_T_C_b38	8.4e-73	-0.49	Whole Blood
		ENSG00000253404.1	AC034243.1	chr5_138881616_T_C_b38	4.4e-70	0.95	Nerve - Tibial
		ENSG00000253404.1	AC034243.1	chr5_138881616_T_C_b38	2.5e-69	1.1	Skin - Not Sun Exposed (Suprapubic)
		ENSG00000253404.1	AC034243.1	chr5_138881616_T_C_b38	1.0e-63	1.0	Breast - Mammary Tissue
		ENSG00000253404.1	AC034243.1	chr5_138881616_T_C_b38	1.1e-62	0.91	Lung
		ENSG00000253404.1	AC034243.1	chr5_138881616_T_C_b38	7.3e-61	1.2	Pituitary
		ENSG00000253404.1	AC034243.1	chr5_138881616_T_C_b38	1.3e-58	0.94	Esophagus - Muscularis
		ENSG00000253404.1	AC034243.1	chr5_138881616_T_C_b38	4.0e-57	0.92	Adipose - Visceral (Omentum)
		ENSG00000253404.1	AC034243.1	chr5_138881616_T_C_b38	1.2e-55	1.1	Heart - Atrial Appendage
		ENSG00000253404.1	AC034243.1	chr5_138881616_T_C_b38	2.5e-54	1.1	Pancreas
		ENSG00000253404.1	AC034243.1	chr5_138881616_T_C_b38	3.5e-52	0.94	Stomach
		ENSG00000253404.1	AC034243.1	chr5_138881616_T_C_b38	1.3e-47	1.1	Adrenal Gland
		ENSG00000253404.1	AC034243.1	chr5_138881616_T_C_b38	6.2e-46	0.78	Cells - Cultured fibroblasts
		ENSG00000253404.1	AC034243.1	chr5_138881616_T_C_b38	1.0e-45	0.93	Heart - Left Ventricle
		ENSG00000253404.1	AC034243.1	chr5_138881616_T_C_b38	1.9e-42	0.91	Colon - Transverse
		ENSG00000044115.20	CTNNA1	chr5_138881616_T_C_b38	3.7e-40	-0.27	Esophagus - Mucosa
		ENSG00000253404.1	AC034243.1	chr5_138881616_T_C_b38	7.9e-40	0.93	Prostate
		ENSG00000253404.1	AC034243.1	chr5_138881616_T_C_b38	4.8e-31	0.89	Colon - Sigmoid
		ENSG00000253404.1	AC034243.1	chr5_138881616_T_C_b38	6.5e-31	0.86	Esophagus - Gastroesophageal Junction
		ENSG00000253404.1	AC034243.1	chr5_138881616_T_C_b38	1.6e-29	1.0	Spleen
		ENSG00000253404.1	AC034243.1	chr5_138881616_T_C_b38	2.0e-27	1.0	Brain - Putamen (basal ganglia)
		ENSG00000253404.1	AC034243.1	chr5_138881616_T_C_b38	1.6e-26	1.1	Brain - Caudate (basal ganglia)
		ENSG00000253404.1	AC034243.1	chr5_138881616_T_C_b38	1.8e-25	1.1	Brain - Nucleus accumbens (basal ganglia)
		ENSG00000253404.1	AC034243.1	chr5_138881616_T_C_b38	4.2e-25	0.92	Liver
		ENSG00000253404.1	AC034243.1	chr5_138881616_T_C_b38	7.8e-24	1.1	Ovary
		ENSG00000253404.1	AC034243.1	chr5_138881616_T_C_b38	1.5e-23	0.49	Testis
		ENSG00000253404.1	AC034243.1	chr5_138881616_T_C_b38	3.1e-22	0.87	Brain - Cortex
		ENSG00000253404.1	AC034243.1	chr5_138881616_T_C_b38	2.0e-21	0.88	Artery - Coronary
		ENSG00000253404.1	AC034243.1	chr5_138881616_T_C_b38	5.0e-21	1.1	Minor Salivary Gland
		ENSG00000120725.12	SIL1	chr5_138881616_T_C_b38	1.7e-19	0.35	Esophagus - Muscularis
		ENSG00000253404.1	AC034243.1	chr5_138881616_T_C_b38	3.8e-19	1.0	Brain - Spinal cord (cervical c-1)
		ENSG00000120725.12	SIL1	chr5_138881616_T_C_b38	1.1e-17	0.21	Nerve - Tibial
		ENSG00000253404.1	AC034243.1	chr5_138881616_T_C_b38	1.6e-17	0.89	Brain - Hypothalamus
		ENSG00000253404.1	AC034243.1	chr5_138881616_T_C_b38	2.3e-17	0.99	Brain - Substantia nigra
		ENSG00000253404.1	AC034243.1	chr5_138881616_T_C_b38	2.9e-17	1.1	Uterus
	rs288029	ENSG00000253404.1	AC034243.1	chr5_138881616_T_C_b38	5.1e-17	1.1	Brain - Amygdala
		ENSG00000253404.1	AC034243.1	chr5_138881616_T_C_b38	5.3e-17	0.92	Brain - Hippocampus
		ENSG00000120725.12	SIL1	chr5_138881616_T_C_b38	5.6e-17	0.22	Skin - Sun Exposed (Lower leg)
		ENSG00000253404.1	AC034243.1	chr5_138881616_T_C_b38	7.2e-16	0.77	Brain - Frontal Cortex (BA9)
		ENSG00000253404.1	AC034243.1	chr5_138881616_T_C_b38	1.1e-15	0.78	Small Intestine - Terminal Ileum
		ENSG00000253404.1	AC034243.1	chr5_138881616_T_C_b38	2.2e-15	1.0	Vagina
		ENSG00000120725.12	SIL1	chr5_138881616_T_C_b38	3.9e-15	0.29	Artery - Aorta
		ENSG00000120725.12	SIL1	chr5_138881616_T_C_b38	1.0e-14	0.22	Skin - Not Sun Exposed (Suprapubic)
		ENSG00000120725.12	SIL1	chr5_138881616_T_C_b38	1.7e-14	0.30	Stomach
		ENSG00000253404.1	AC034243.1	chr5_138881616_T_C_b38	2.3e-14	0.75	Brain - Anterior cingulate cortex (BA24)
		ENSG00000120725.12	SIL1	chr5_138881616_T_C_b38	9.5e-12	0.14	Thyroid
		ENSG00000120725.12	SIL1	chr5_138881616_T_C_b38	2.5e-10	0.16	Artery - Tibial
		ENSG00000120725.12	SIL1	chr5_138881616_T_C_b38	3.6e-10	0.15	Lung
		ENSG00000253404.1	AC034243.1	chr5_138881616_T_C_b38	3.6e-10	1.0	Kidney - Cortex
		ENSG00000120725.12	SIL1	chr5_138881616_T_C_b38	1.1e-9	0.16	Adipose - Subcutaneous
		ENSG00000044115.20	CTNNA1	chr5_138881616_T_C_b38	1.3e-9	-0.11	Nerve - Tibial
		ENSG00000044115.20	CTNNA1	chr5_138881616_T_C_b38	2.5e-9	-0.14	Cells - Cultured fibroblasts
		ENSG00000120725.12	SIL1	chr5_138881616_T_C_b38	2.9e-9	0.15	Adipose - Visceral (Omentum)
		ENSG00000120725.12	SIL1	chr5_138881616_T_C_b38	1.5e-8	0.29	Esophagus - Gastroesophageal Junction
		ENSG00000120725.12	SIL1	chr5_138881616_T_C_b38	4.9e-8	0.27	Pituitary
		ENSG00000120725.12	SIL1	chr5_138881616_T_C_b38	1.0e-7	0.40	Brain - Frontal Cortex (BA9)
		ENSG00000044115.20	CTNNA1	chr5_138881616_T_C_b38	1.3e-7	-0.10	Thyroid
		ENSG00000120725.12	SIL1	chr5_138881616_T_C_b38	1.8e-7	0.12	Cells - Cultured fibroblasts
		ENSG00000120725.12	SIL1	chr5_138881616_T_C_b38	2.3e-7	0.16	Esophagus - Mucosa
		ENSG00000044115.20	CTNNA1	chr5_138881616_T_C_b38	2.8e-7	0.10	Testis
		ENSG00000120725.12	SIL1	chr5_138881616_T_C_b38	3.1e-7	0.33	Brain - Caudate (basal ganglia)
		ENSG00000120725.12	SIL1	chr5_138881616_T_C_b38	3.2e-7	0.32	Brain - Cortex
		ENSG00000044115.20	CTNNA1	chr5_138881616_T_C_b38	4.6e-7	-0.099	Skin - Not Sun Exposed (Suprapubic)
		ENSG00000044115.20	CTNNA1	chr5_138881616_T_C_b38	5.6e-7	-0.14	Breast - Mammary Tissue
		ENSG00000044115.20	CTNNA1	chr5_138881616_T_C_b38	5.7e-7	-0.14	Colon - Transverse
		ENSG00000120725.12	SIL1	chr5_138881616_T_C_b38	9.0e-7	0.22	Adrenal Gland
		ENSG00000044115.20	CTNNA1	chr5_138881616_T_C_b38	0.0000024	-0.15	Spleen
		ENSG00000044115.20	CTNNA1	chr5_138881616_T_C_b38	0.0000025	-0.14	Muscle - Skeletal
		ENSG00000120725.12	SIL1	chr5_138881616_T_C_b38	0.0000039	0.33	Brain - Nucleus accumbens (basal ganglia)
		ENSG00000120725.12	SIL1	chr5_138881616_T_C_b38	0.0000039	0.42	Brain - Substantia nigra
		ENSG00000120725.12	SIL1	chr5_138881616_T_C_b38	0.0000048	0.14	Breast - Mammary Tissue
		ENSG00000120725.12	SIL1	chr5_138881616_T_C_b38	0.0000064	0.26	Brain - Cerebellum
		ENSG00000044115.20	CTNNA1	chr5_138881616_T_C_b38	0.0000075	-0.12	Heart - Atrial Appendage
		ENSG00000120725.12	SIL1	chr5_138881616_T_C_b38	0.0000096	0.18	Colon - Transverse
		ENSG00000044115.20	CTNNA1	chr5_138881616_T_C_b38	0.000011	-0.080	Skin - Sun Exposed (Lower leg)

		ENSG00000120725.12	SIL1	chr5_138881616_T_C_b38	0.000013	0.28	Artery - Coronary
		ENSG00000120725.12	SIL1	chr5_138881616_T_C_b38	0.000018	0.13	Heart - Left Ventricle
		ENSG00000120725.12	SIL1	chr5_138881616_T_C_b38	0.000019	0.16	Pancreas
		ENSG00000044115.20	CTNNA1	chr5_138881616_T_C_b38	0.000034	-0.20	Brain - Cerebellar Hemisphere
		ENSG00000120725.12	SIL1	chr5_138881616_T_C_b38	0.000050	0.31	Brain - Anterior cingulate cortex (BA24)
		ENSG00000044115.20	CTNNA1	chr5_138881616_T_C_b38	0.000060	-0.10	Adipose - Subcutaneous
		ENSG00000120725.12	SIL1	chr5_138881616_T_C_b38	0.000080	0.26	Brain - Putamen (basal ganglia)
		ENSG00000044115.20	CTNNA1	chr5_138881616_T_C_b38	0.000098	-0.17	Brain - Cerebellum
	rs288028	ENSG00000253404.1	AC034243.1	chr5_138886300_G_C_b38	2.9e-130	1.2	Thyroid
		ENSG00000253404.1	AC034243.1	chr5_138886300_G_C_b38	4.3e-92	1.1	Skin - Sun Exposed (Lower leg)
		ENSG00000253404.1	AC034243.1	chr5_138886300_G_C_b38	5.6e-85	1.0	Adipose - Subcutaneous
		ENSG00000253404.1	AC034243.1	chr5_138886300_G_C_b38	3.0e-74	1.1	Esophagus - Mucosa
		ENSG00000044115.20	CTNNA1	chr5_138886300_G_C_b38	9.9e-74	-0.49	Whole Blood
		ENSG00000253404.1	AC034243.1	chr5_138886300_G_C_b38	9.1e-70	1.0	Skin - Not Sun Exposed (Suprapubic)
		ENSG00000253404.1	AC034243.1	chr5_138886300_G_C_b38	9.7e-69	0.93	Nerve - Tibial
		ENSG00000253404.1	AC034243.1	chr5_138886300_G_C_b38	2.8e-65	1.2	Pituitary
		ENSG00000253404.1	AC034243.1	chr5_138886300_G_C_b38	8.3e-64	0.91	Lung
		ENSG00000253404.1	AC034243.1	chr5_138886300_G_C_b38	9.9e-63	1.0	Breast - Mammary Tissue
		ENSG00000253404.1	AC034243.1	chr5_138886300_G_C_b38	5.4e-57	0.92	Esophagus - Muscularis
		ENSG00000253404.1	AC034243.1	chr5_138886300_G_C_b38	2.3e-56	0.91	Adipose - Visceral (Omentum)
		ENSG00000253404.1	AC034243.1	chr5_138886300_G_C_b38	2.4e-55	1.2	Pancreas
		ENSG00000253404.1	AC034243.1	chr5_138886300_G_C_b38	9.5e-55	1.0	Heart - Atrial Appendage
		ENSG00000253404.1	AC034243.1	chr5_138886300_G_C_b38	1.4e-53	0.94	Stomach
		ENSG00000253404.1	AC034243.1	chr5_138886300_G_C_b38	1.3e-49	0.95	Heart - Left Ventricle
		ENSG00000253404.1	AC034243.1	chr5_138886300_G_C_b38	1.9e-47	1.1	Adrenal Gland
		ENSG00000253404.1	AC034243.1	chr5_138886300_G_C_b38	7.4e-46	0.77	Cells - Cultured fibroblasts
		ENSG00000253404.1	AC034243.1	chr5_138886300_G_C_b38	1.1e-41	0.89	Colon - Transverse
		ENSG00000044115.20	CTNNA1	chr5_138886300_G_C_b38	2.2e-40	-0.27	Esophagus - Mucosa
		ENSG00000253404.1	AC034243.1	chr5_138886300_G_C_b38	3.0e-37	0.91	Prostate
		ENSG00000253404.1	AC034243.1	chr5_138886300_G_C_b38	1.5e-34	0.89	Esophagus - Gastroesophageal Junction
		ENSG00000253404.1	AC034243.1	chr5_138886300_G_C_b38	3.1e-30	0.87	Colon - Sigmoid
		ENSG00000253404.1	AC034243.1	chr5_138886300_G_C_b38	1.0e-28	1.0	Brain - Putamen (basal ganglia)
		ENSG00000253404.1	AC034243.1	chr5_138886300_G_C_b38	1.2e-28	1.1	Brain - Caudate (basal ganglia)
		ENSG00000253404.1	AC034243.1	chr5_138886300_G_C_b38	2.6e-28	1.0	Spleen
		ENSG00000253404.1	AC034243.1	chr5_138886300_G_C_b38	6.1e-26	1.1	Ovary
		ENSG00000253404.1	AC034243.1	chr5_138886300_G_C_b38	9.0e-26	0.94	Liver
		ENSG00000253404.1	AC034243.1	chr5_138886300_G_C_b38	1.2e-25	1.0	Brain - Nucleus accumbens (basal ganglia)
		ENSG00000253404.1	AC034243.1	chr5_138886300_G_C_b38	5.6e-23	0.48	Testis
		ENSG00000253404.1	AC034243.1	chr5_138886300_G_C_b38	9.2e-23	0.87	Brain - Cortex
		ENSG00000253404.1	AC034243.1	chr5_138886300_G_C_b38	3.4e-21	1.1	Minor Salivary Gland
		ENSG00000253404.1	AC034243.1	chr5_138886300_G_C_b38	1.3e-20	0.85	Artery - Coronary
		ENSG00000253404.1	AC034243.1	chr5_138886300_G_C_b38	7.6e-20	1.0	Brain - Spinal cord (cervical c-1)
		ENSG00000253404.1	AC034243.1	chr5_138886300_G_C_b38	3.2e-19	0.94	Brain - Hippocampus
		ENSG00000253404.1	AC034243.1	chr5_138886300_G_C_b38	1.7e-18	0.99	Brain - Substantia nigra
		ENSG00000120725.12	SIL1	chr5_138886300_G_C_b38	2.7e-18	0.34	Esophagus - Muscularis
		ENSG00000253404.1	AC034243.1	chr5_138886300_G_C_b38	3.4e-18	0.90	Brain - Hypothalamus
		ENSG00000120725.12	SIL1	chr5_138886300_G_C_b38	6.3e-18	0.21	Nerve - Tibial
		ENSG00000253404.1	AC034243.1	chr5_138886300_G_C_b38	3.7e-17	1.1	Uterus
		ENSG00000253404.1	AC034243.1	chr5_138886300_G_C_b38	5.7e-17	1.1	Brain - Amygdala
		ENSG00000253404.1	AC034243.1	chr5_138886300_G_C_b38	5.8e-17	0.79	Brain - Frontal Cortex (BA9)
		ENSG00000120725.12	SIL1	chr5_138886300_G_C_b38	8.2e-17	0.22	Skin - Sun Exposed (Lower leg)
		ENSG00000253404.1	AC034243.1	chr5_138886300_G_C_b38	4.5e-16	1.1	Vagina
		ENSG00000253404.1	AC034243.1	chr5_138886300_G_C_b38	5.3e-16	0.79	Brain - Anterior cingulate cortex (BA24)
		ENSG00000253404.1	AC034243.1	chr5_138886300_G_C_b38	1.0e-15	0.78	Small Intestine - Terminal Ileum
		ENSG00000120725.12	SIL1	chr5_138886300_G_C_b38	1.3e-15	0.22	Skin - Not Sun Exposed (Suprapubic)
		ENSG00000120725.12	SIL1	chr5_138886300_G_C_b38	1.7e-15	0.29	Artery - Aorta
		ENSG00000120725.12	SIL1	chr5_138886300_G_C_b38	4.5e-14	0.29	Stomach
		ENSG00000253404.1	AC034243.1	chr5_138886300_G_C_b38	1.4e-12	1.1	Kidney - Cortex
		ENSG00000120725.12	SIL1	chr5_138886300_G_C_b38	1.5e-12	0.15	Thyroid
		ENSG00000120725.12	SIL1	chr5_138886300_G_C_b38	3.4e-10	0.16	Artery - Tibial
		ENSG00000120725.12	SIL1	chr5_138886300_G_C_b38	7.6e-10	0.16	Adipose - Subcutaneous
		ENSG00000120725.12	SIL1	chr5_138886300_G_C_b38	1.9e-9	0.30	Esophagus - Gastroesophageal Junction
		ENSG00000120725.12	SIL1	chr5_138886300_G_C_b38	3.6e-9	0.15	Adipose - Visceral (Omentum)
		ENSG00000120725.12	SIL1	chr5_138886300_G_C_b38	3.8e-9	0.14	Lung
		ENSG00000044115.20	CTNNA1	chr5_138886300_G_C_b38	4.0e-9	-0.11	Nerve - Tibial
		ENSG00000044115.20	CTNNA1	chr5_138886300_G_C_b38	5.2e-9	-0.13	Cells - Cultured fibroblasts
		ENSG00000120725.12	SIL1	chr5_138886300_G_C_b38	7.5e-9	0.43	Brain - Frontal Cortex (BA9)
		ENSG00000044115.20	CTNNA1	chr5_138886300_G_C_b38	2.1e-8	-0.15	Breast - Mammary Tissue
		ENSG00000120725.12	SIL1	chr5_138886300_G_C_b38	5.6e-8	0.12	Cells - Cultured fibroblasts
		ENSG00000120725.12	SIL1	chr5_138886300_G_C_b38	6.6e-8	0.26	Pituitary
		ENSG00000044115.20	CTNNA1	chr5_138886300_G_C_b38	1.9e-7	-0.15	Colon - Transverse
		ENSG00000044115.20	CTNNA1	chr5_138886300_G_C_b38	2.3e-7	0.10	Testis
		ENSG00000044115.20	CTNNA1	chr5_138886300_G_C_b38	2.6e-7	-0.097	Thyroid
		ENSG00000120725.12	SIL1	chr5_138886300_G_C_b38	3.5e-7	0.29	Brain - Cerebellum
		ENSG00000120725.12	SIL1	chr5_138886300_G_C_b38	3.9e-7	0.31	Brain - Cortex
		ENSG00000120725.12	SIL1	chr5_138886300_G_C_b38	4.2e-7	0.33	Brain - Caudate (basal ganglia)
		ENSG00000044115.20	CTNNA1	chr5_138886300_G_C_b38	4.8e-7	-0.097	Skin - Not Sun Exposed (Suprapubic)
		ENSG00000120725.12	SIL1	chr5_138886300_G_C_b38	7.5e-7	0.15	Esophagus - Mucosa
		ENSG00000044115.20	CTNNA1	chr5_138886300_G_C_b38	0.0000012	-0.13	Heart - Atrial Appendage
		ENSG00000044115.20	CTNNA1	chr5_138886300_G_C_b38	0.0000013	-0.21	Brain - Cerebellum
		ENSG00000120725.12	SIL1	chr5_138886300_G_C_b38	0.0000024	0.34	Brain - Nucleus accumbens (basal ganglia)
		ENSG00000120725.12	SIL1	chr5_138886300_G_C_b38	0.0000026	0.22	Adrenal Gland

		ENSG00000044115.20	CTNNA1	chr5_138886300_G_C_b38	0.0000030	-0.14	Spleen
		ENSG00000120725.12	SIL1	chr5_138886300_G_C_b38	0.0000034	0.41	Brain - Substantia nigra
		ENSG00000044115.20	CTNNA1	chr5_138886300_G_C_b38	0.0000047	-0.083	Skin - Sun Exposed (Lower leg)
		ENSG00000044115.20	CTNNA1	chr5_138886300_G_C_b38	0.0000054	-0.13	Muscle - Skeletal
		ENSG00000120725.12	SIL1	chr5_138886300_G_C_b38	0.0000081	0.18	Colon - Transverse
		ENSG00000120725.12	SIL1	chr5_138886300_G_C_b38	0.000012	0.13	Heart - Left Ventricle
		ENSG00000120725.12	SIL1	chr5_138886300_G_C_b38	0.000024	0.32	Brain - Anterior cingulate cortex (BA24)
		ENSG00000044115.20	CTNNA1	chr5_138886300_G_C_b38	0.000025	-0.20	Brain - Cerebellar Hemisphere
		ENSG00000120725.12	SIL1	chr5_138886300_G_C_b38	0.000039	0.26	Artery - Coronary
		ENSG00000044115.20	CTNNA1	chr5_138886300_G_C_b38	0.000042	-0.12	Pancreas
		ENSG00000120725.12	SIL1	chr5_138886300_G_C_b38	0.000047	0.12	Breast - Mammary Tissue
		ENSG00000044115.20	CTNNA1	chr5_138886300_G_C_b38	0.000050	-0.10	Adipose - Subcutaneous
		ENSG00000120725.12	SIL1	chr5_138886300_G_C_b38	0.000075	0.26	Brain - Putamen (basal ganglia)
	rs288026	ENSG00000253404.1	AC034243.1	chr5_138886905_A_G_b38	2.9e-130	1.2	Thyroid
		ENSG00000253404.1	AC034243.1	chr5_138886905_A_G_b38	4.3e-92	1.1	Skin - Sun Exposed (Lower leg)
		ENSG00000253404.1	AC034243.1	chr5_138886905_A_G_b38	5.6e-85	1.0	Adipose - Subcutaneous
		ENSG00000253404.1	AC034243.1	chr5_138886905_A_G_b38	3.0e-74	1.1	Esophagus - Mucosa
		ENSG00000044115.20	CTNNA1	chr5_138886905_A_G_b38	9.9e-74	-0.49	Whole Blood
		ENSG00000253404.1	AC034243.1	chr5_138886905_A_G_b38	9.1e-70	1.0	Skin - Not Sun Exposed (Suprapubic)
		ENSG00000253404.1	AC034243.1	chr5_138886905_A_G_b38	9.7e-69	0.93	Nerve - Tibial
		ENSG00000253404.1	AC034243.1	chr5_138886905_A_G_b38	2.8e-65	1.2	Pituitary
		ENSG00000253404.1	AC034243.1	chr5_138886905_A_G_b38	8.3e-64	0.91	Lung
		ENSG00000253404.1	AC034243.1	chr5_138886905_A_G_b38	9.9e-63	1.0	Breast - Mammary Tissue
		ENSG00000253404.1	AC034243.1	chr5_138886905_A_G_b38	5.4e-57	0.92	Esophagus - Muscularis
		ENSG00000253404.1	AC034243.1	chr5_138886905_A_G_b38	2.3e-56	0.91	Adipose - Visceral (Omentum)
		ENSG00000253404.1	AC034243.1	chr5_138886905_A_G_b38	2.4e-55	1.2	Pancreas
		ENSG00000253404.1	AC034243.1	chr5_138886905_A_G_b38	9.5e-55	1.0	Heart - Atrial Appendage
		ENSG00000253404.1	AC034243.1	chr5_138886905_A_G_b38	1.4e-53	0.94	Stomach
		ENSG00000253404.1	AC034243.1	chr5_138886905_A_G_b38	1.3e-49	0.95	Heart - Left Ventricle
		ENSG00000253404.1	AC034243.1	chr5_138886905_A_G_b38	1.9e-47	1.1	Adrenal Gland
		ENSG00000253404.1	AC034243.1	chr5_138886905_A_G_b38	7.4e-46	0.77	Cells - Cultured fibroblasts
		ENSG00000253404.1	AC034243.1	chr5_138886905_A_G_b38	1.1e-41	0.89	Colon - Transverse
		ENSG00000044115.20	CTNNA1	chr5_138886905_A_G_b38	2.2e-40	-0.27	Esophagus - Mucosa
		ENSG00000253404.1	AC034243.1	chr5_138886905_A_G_b38	3.0e-37	0.91	Prostate
		ENSG00000253404.1	AC034243.1	chr5_138886905_A_G_b38	1.5e-34	0.89	Esophagus - Gastroesophageal Junction
		ENSG00000253404.1	AC034243.1	chr5_138886905_A_G_b38	3.1e-30	0.87	Colon - Sigmoid
		ENSG00000253404.1	AC034243.1	chr5_138886905_A_G_b38	1.0e-28	1.0	Brain - Putamen (basal ganglia)
		ENSG00000253404.1	AC034243.1	chr5_138886905_A_G_b38	1.2e-28	1.1	Brain - Caudate (basal ganglia)
		ENSG00000253404.1	AC034243.1	chr5_138886905_A_G_b38	2.6e-28	1.0	Spleen
		ENSG00000253404.1	AC034243.1	chr5_138886905_A_G_b38	6.1e-26	1.1	Ovary
		ENSG00000253404.1	AC034243.1	chr5_138886905_A_G_b38	9.0e-26	0.94	Liver
		ENSG00000253404.1	AC034243.1	chr5_138886905_A_G_b38	1.2e-25	1.0	Brain - Nucleus accumbens (basal ganglia)
		ENSG00000253404.1	AC034243.1	chr5_138886905_A_G_b38	5.6e-23	0.48	Testis
		ENSG00000253404.1	AC034243.1	chr5_138886905_A_G_b38	9.2e-23	0.87	Brain - Cortex
		ENSG00000253404.1	AC034243.1	chr5_138886905_A_G_b38	3.4e-21	1.1	Minor Salivary Gland
		ENSG00000253404.1	AC034243.1	chr5_138886905_A_G_b38	1.3e-20	0.85	Artery - Coronary
		ENSG00000253404.1	AC034243.1	chr5_138886905_A_G_b38	7.6e-20	1.0	Brain - Spinal cord (cervical c-1)
		ENSG00000253404.1	AC034243.1	chr5_138886905_A_G_b38	3.2e-19	0.94	Brain - Hippocampus
		ENSG00000253404.1	AC034243.1	chr5_138886905_A_G_b38	1.7e-18	0.99	Brain - Substantia nigra
		ENSG00000120725.12	SIL1	chr5_138886905_A_G_b38	2.7e-18	0.34	Esophagus - Muscularis
		ENSG00000253404.1	AC034243.1	chr5_138886905_A_G_b38	3.4e-18	0.90	Brain - Hypothalamus
		ENSG00000120725.12	SIL1	chr5_138886905_A_G_b38	6.3e-18	0.21	Nerve - Tibial
		ENSG00000253404.1	AC034243.1	chr5_138886905_A_G_b38	3.7e-17	1.1	Uterus
		ENSG00000253404.1	AC034243.1	chr5_138886905_A_G_b38	5.7e-17	1.1	Brain - Amygdala
		ENSG00000253404.1	AC034243.1	chr5_138886905_A_G_b38	5.8e-17	0.79	Brain - Frontal Cortex (BA9)
		ENSG00000120725.12	SIL1	chr5_138886905_A_G_b38	8.2e-17	0.22	Skin - Sun Exposed (Lower leg)
		ENSG00000253404.1	AC034243.1	chr5_138886905_A_G_b38	4.5e-16	1.1	Vagina
		ENSG00000253404.1	AC034243.1	chr5_138886905_A_G_b38	5.3e-16	0.79	Brain - Anterior cingulate cortex (BA24)
		ENSG00000253404.1	AC034243.1	chr5_138886905_A_G_b38	1.0e-15	0.78	Small Intestine - Terminal Ileum
		ENSG00000120725.12	SIL1	chr5_138886905_A_G_b38	1.3e-15	0.22	Skin - Not Sun Exposed (Suprapubic)
		ENSG00000120725.12	SIL1	chr5_138886905_A_G_b38	1.7e-15	0.29	Artery - Aorta
		ENSG00000120725.12	SIL1	chr5_138886905_A_G_b38	4.5e-14	0.29	Stomach
		ENSG00000253404.1	AC034243.1	chr5_138886905_A_G_b38	1.4e-12	1.1	Kidney - Cortex
		ENSG00000120725.12	SIL1	chr5_138886905_A_G_b38	1.5e-12	0.15	Thyroid
		ENSG00000120725.12	SIL1	chr5_138886905_A_G_b38	3.4e-10	0.16	Artery - Tibial
		ENSG00000120725.12	SIL1	chr5_138886905_A_G_b38	7.6e-10	0.16	Adipose - Subcutaneous
		ENSG00000120725.12	SIL1	chr5_138886905_A_G_b38	1.9e-9	0.30	Esophagus - Gastroesophageal Junction
		ENSG00000120725.12	SIL1	chr5_138886905_A_G_b38	3.6e-9	0.15	Adipose - Visceral (Omentum)
		ENSG00000120725.12	SIL1	chr5_138886905_A_G_b38	3.8e-9	0.14	Lung
		ENSG00000044115.20	CTNNA1	chr5_138886905_A_G_b38	4.0e-9	-0.11	Nerve - Tibial
		ENSG00000044115.20	CTNNA1	chr5_138886905_A_G_b38	5.2e-9	-0.13	Cells - Cultured fibroblasts
		ENSG00000120725.12	SIL1	chr5_138886905_A_G_b38	7.5e-9	0.43	Brain - Frontal Cortex (BA9)
		ENSG00000044115.20	CTNNA1	chr5_138886905_A_G_b38	2.1e-8	-0.15	Breast - Mammary Tissue
		ENSG00000120725.12	SIL1	chr5_138886905_A_G_b38	5.6e-8	0.12	Cells - Cultured fibroblasts
		ENSG00000120725.12	SIL1	chr5_138886905_A_G_b38	6.6e-8	0.26	Pituitary
		ENSG00000044115.20	CTNNA1	chr5_138886905_A_G_b38	1.9e-7	-0.15	Colon - Transverse
		ENSG00000044115.20	CTNNA1	chr5_138886905_A_G_b38	2.3e-7	0.10	Testis
		ENSG00000044115.20	CTNNA1	chr5_138886905_A_G_b38	2.6e-7	-0.097	Thyroid
		ENSG00000120725.12	SIL1	chr5_138886905_A_G_b38	3.5e-7	0.29	Brain - Cerebellum
		ENSG00000120725.12	SIL1	chr5_138886905_A_G_b38	3.9e-7	0.31	Brain - Cortex
		ENSG00000120725.12	SIL1	chr5_138886905_A_G_b38	4.2e-7	0.33	Brain - Caudate (basal ganglia)
		ENSG00000044115.20	CTNNA1	chr5_138886905_A_G_b38	4.8e-7	-0.097	Skin - Not Sun Exposed (Suprapubic)

		ENSG00000120725.12	SIL1	chr5_138886905_A_G_b38	7.5e-7	0.15	Esophagus - Mucosa
		ENSG00000044115.20	CTNNA1	chr5_138886905_A_G_b38	0.0000012	-0.13	Heart - Atrial Appendage
		ENSG00000044115.20	CTNNA1	chr5_138886905_A_G_b38	0.0000013	-0.21	Brain - Cerebellum
		ENSG00000120725.12	SIL1	chr5_138886905_A_G_b38	0.0000024	0.34	Brain - Nucleus accumbens (basal ganglia)
		ENSG00000120725.12	SIL1	chr5_138886905_A_G_b38	0.0000026	0.22	Adrenal Gland
		ENSG00000044115.20	CTNNA1	chr5_138886905_A_G_b38	0.0000030	-0.14	Spleen
		ENSG00000120725.12	SIL1	chr5_138886905_A_G_b38	0.0000034	0.41	Brain - Substantia nigra
		ENSG00000044115.20	CTNNA1	chr5_138886905_A_G_b38	0.0000047	-0.083	Skin - Sun Exposed (Lower leg)
		ENSG00000044115.20	CTNNA1	chr5_138886905_A_G_b38	0.0000054	-0.13	Muscle - Skeletal
		ENSG00000120725.12	SIL1	chr5_138886905_A_G_b38	0.0000081	0.18	Colon - Transverse
		ENSG00000120725.12	SIL1	chr5_138886905_A_G_b38	0.000012	0.13	Heart - Left Ventricle
		ENSG00000120725.12	SIL1	chr5_138886905_A_G_b38	0.000024	0.32	Brain - Anterior cingulate cortex (BA24)
		ENSG00000044115.20	CTNNA1	chr5_138886905_A_G_b38	0.000025	-0.20	Brain - Cerebellar Hemisphere
		ENSG00000120725.12	SIL1	chr5_138886905_A_G_b38	0.000039	0.26	Artery - Coronary
		ENSG00000044115.20	CTNNA1	chr5_138886905_A_G_b38	0.000042	-0.12	Pancreas
		ENSG00000120725.12	SIL1	chr5_138886905_A_G_b38	0.000047	0.12	Breast - Mammary Tissue
		ENSG00000044115.20	CTNNA1	chr5_138886905_A_G_b38	0.000050	-0.10	Adipose - Subcutaneous
		ENSG00000120725.12	SIL1	chr5_138886905_A_G_b38	0.000075	0.26	Brain - Putamen (basal ganglia)
	rs288024	ENSG00000253404.1	AC034243.1	chr5_138888103_A_G_b38	1.6e-129	1.2	Thyroid
		ENSG00000253404.1	AC034243.1	chr5_138888103_A_G_b38	3.0e-92	1.1	Skin - Sun Exposed (Lower leg)
		ENSG00000253404.1	AC034243.1	chr5_138888103_A_G_b38	6.6e-85	1.0	Adipose - Subcutaneous
		ENSG00000044115.20	CTNNA1	chr5_138888103_A_G_b38	7.8e-74	-0.49	Whole Blood
		ENSG00000253404.1	AC034243.1	chr5_138888103_A_G_b38	2.8e-73	1.1	Esophagus - Mucosa
		ENSG00000253404.1	AC034243.1	chr5_138888103_A_G_b38	3.8e-69	1.0	Skin - Not Sun Exposed (Suprapubic)
		ENSG00000253404.1	AC034243.1	chr5_138888103_A_G_b38	2.8e-68	0.93	Nerve - Tibial
		ENSG00000253404.1	AC034243.1	chr5_138888103_A_G_b38	8.6e-65	1.2	Pituitary
		ENSG00000253404.1	AC034243.1	chr5_138888103_A_G_b38	5.8e-64	0.92	Lung
		ENSG00000253404.1	AC034243.1	chr5_138888103_A_G_b38	7.7e-62	1.0	Breast - Mammary Tissue
		ENSG00000253404.1	AC034243.1	chr5_138888103_A_G_b38	5.4e-57	0.92	Adipose - Visceral (Omentum)
		ENSG00000253404.1	AC034243.1	chr5_138888103_A_G_b38	8.3e-57	0.92	Esophagus - Muscularis
		ENSG00000253404.1	AC034243.1	chr5_138888103_A_G_b38	2.4e-55	1.2	Pancreas
		ENSG00000253404.1	AC034243.1	chr5_138888103_A_G_b38	9.5e-55	1.0	Heart - Atrial Appendage
		ENSG00000253404.1	AC034243.1	chr5_138888103_A_G_b38	1.4e-53	0.94	Stomach
		ENSG00000253404.1	AC034243.1	chr5_138888103_A_G_b38	3.8e-49	0.95	Heart - Left Ventricle
		ENSG00000253404.1	AC034243.1	chr5_138888103_A_G_b38	1.9e-47	1.1	Adrenal Gland
		ENSG00000253404.1	AC034243.1	chr5_138888103_A_G_b38	7.1e-46	0.77	Cells - Cultured fibroblasts
		ENSG00000253404.1	AC034243.1	chr5_138888103_A_G_b38	1.1e-41	0.89	Colon - Transverse
		ENSG00000044115.20	CTNNA1	chr5_138888103_A_G_b38	5.8e-40	-0.27	Esophagus - Mucosa
		ENSG00000253404.1	AC034243.1	chr5_138888103_A_G_b38	3.0e-37	0.91	Prostate
		ENSG00000253404.1	AC034243.1	chr5_138888103_A_G_b38	1.5e-34	0.89	Esophagus - Gastroesophageal Junction
		ENSG00000253404.1	AC034243.1	chr5_138888103_A_G_b38	3.1e-30	0.87	Colon - Sigmoid
		ENSG00000253404.1	AC034243.1	chr5_138888103_A_G_b38	4.1e-29	1.0	Brain - Putamen (basal ganglia)
		ENSG00000253404.1	AC034243.1	chr5_138888103_A_G_b38	5.5e-29	1.1	Brain - Caudate (basal ganglia)
		ENSG00000253404.1	AC034243.1	chr5_138888103_A_G_b38	9.3e-29	1.0	Spleen
		ENSG00000253404.1	AC034243.1	chr5_138888103_A_G_b38	4.9e-26	0.94	Liver
		ENSG00000253404.1	AC034243.1	chr5_138888103_A_G_b38	6.2e-26	1.1	Ovary
		ENSG00000253404.1	AC034243.1	chr5_138888103_A_G_b38	8.8e-26	1.1	Brain - Nucleus accumbens (basal ganglia)
		ENSG00000253404.1	AC034243.1	chr5_138888103_A_G_b38	5.6e-23	0.48	Testis
		ENSG00000253404.1	AC034243.1	chr5_138888103_A_G_b38	7.9e-23	0.87	Brain - Cortex
		ENSG00000253404.1	AC034243.1	chr5_138888103_A_G_b38	3.4e-21	1.1	Minor Salivary Gland
		ENSG00000253404.1	AC034243.1	chr5_138888103_A_G_b38	1.3e-20	0.85	Artery - Coronary
		ENSG00000253404.1	AC034243.1	chr5_138888103_A_G_b38	7.6e-20	1.0	Brain - Spinal cord (cervical c-1)
		ENSG00000253404.1	AC034243.1	chr5_138888103_A_G_b38	3.6e-19	0.94	Brain - Hippocampus
		ENSG00000253404.1	AC034243.1	chr5_138888103_A_G_b38	1.7e-18	0.99	Brain - Substantia nigra
		ENSG00000120725.12	SIL1	chr5_138888103_A_G_b38	2.5e-18	0.34	Esophagus - Muscularis
		ENSG00000253404.1	AC034243.1	chr5_138888103_A_G_b38	3.4e-18	0.90	Brain - Hypothalamus
		ENSG00000120725.12	SIL1	chr5_138888103_A_G_b38	5.7e-18	0.21	Nerve - Tibial
		ENSG00000253404.1	AC034243.1	chr5_138888103_A_G_b38	2.9e-17	0.80	Brain - Frontal Cortex (BA9)
		ENSG00000253404.1	AC034243.1	chr5_138888103_A_G_b38	3.7e-17	1.1	Uterus
		ENSG00000120725.12	SIL1	chr5_138888103_A_G_b38	7.6e-17	0.22	Skin - Sun Exposed (Lower leg)
		ENSG00000253404.1	AC034243.1	chr5_138888103_A_G_b38	1.0e-16	1.1	Brain - Amygdala
		ENSG00000253404.1	AC034243.1	chr5_138888103_A_G_b38	4.5e-16	1.1	Vagina
		ENSG00000253404.1	AC034243.1	chr5_138888103_A_G_b38	5.2e-16	0.79	Brain - Anterior cingulate cortex (BA24)
		ENSG00000253404.1	AC034243.1	chr5_138888103_A_G_b38	1.0e-15	0.78	Small Intestine - Terminal Ileum
		ENSG00000120725.12	SIL1	chr5_138888103_A_G_b38	1.5e-15	0.22	Skin - Not Sun Exposed (Suprapubic)
		ENSG00000120725.12	SIL1	chr5_138888103_A_G_b38	1.7e-15	0.29	Artery - Aorta
		ENSG00000120725.12	SIL1	chr5_138888103_A_G_b38	4.5e-14	0.29	Stomach
		ENSG00000253404.1	AC034243.1	chr5_138888103_A_G_b38	5.9e-13	1.1	Kidney - Cortex
		ENSG00000120725.12	SIL1	chr5_138888103_A_G_b38	1.3e-12	0.15	Thyroid
		ENSG00000120725.12	SIL1	chr5_138888103_A_G_b38	3.1e-10	0.16	Artery - Tibial
		ENSG00000120725.12	SIL1	chr5_138888103_A_G_b38	6.7e-10	0.17	Adipose - Subcutaneous
		ENSG00000120725.12	SIL1	chr5_138888103_A_G_b38	1.9e-9	0.30	Esophagus - Gastroesophageal Junction
		ENSG00000120725.12	SIL1	chr5_138888103_A_G_b38	3.3e-9	0.14	Lung
		ENSG00000044115.20	CTNNA1	chr5_138888103_A_G_b38	3.3e-9	-0.11	Nerve - Tibial
		ENSG00000120725.12	SIL1	chr5_138888103_A_G_b38	3.9e-9	0.15	Adipose - Visceral (Omentum)
		ENSG00000120725.12	SIL1	chr5_138888103_A_G_b38	6.4e-9	0.44	Brain - Frontal Cortex (BA9)
		ENSG00000044115.20	CTNNA1	chr5_138888103_A_G_b38	7.5e-9	-0.13	Cells - Cultured fibroblasts
		ENSG00000044115.20	CTNNA1	chr5_138888103_A_G_b38	2.4e-8	-0.15	Breast - Mammary Tissue
		ENSG00000120725.12	SIL1	chr5_138888103_A_G_b38	4.0e-8	0.13	Cells - Cultured fibroblasts
		ENSG00000120725.12	SIL1	chr5_138888103_A_G_b38	4.5e-8	0.27	Pituitary
		ENSG00000044115.20	CTNNA1	chr5_138888103_A_G_b38	1.9e-7	-0.15	Colon - Transverse
		ENSG00000044115.20	CTNNA1	chr5_138888103_A_G_b38	2.3e-7	0.10	Testis

		ENSG00000044115.20	CTNNA1	chr5_138888103_A_G_b38	3.2e-7	-0.096	Thyroid
		ENSG00000120725.12	SIL1	chr5_138888103_A_G_b38	4.7e-7	0.31	Brain - Cortex
		ENSG00000044115.20	CTNNA1	chr5_138888103_A_G_b38	4.9e-7	-0.097	Skin - Not Sun Exposed (Suprapubic)
		ENSG00000120725.12	SIL1	chr5_138888103_A_G_b38	5.0e-7	0.33	Brain - Caudate (basal ganglia)
		ENSG00000120725.12	SIL1	chr5_138888103_A_G_b38	6.1e-7	0.15	Esophagus - Mucosa
		ENSG00000120725.12	SIL1	chr5_138888103_A_G_b38	7.1e-7	0.28	Brain - Cerebellum
		ENSG00000044115.20	CTNNA1	chr5_138888103_A_G_b38	9.2e-7	-0.21	Brain - Cerebellum
		ENSG00000044115.20	CTNNA1	chr5_138888103_A_G_b38	0.0000012	-0.13	Heart - Atrial Appendage
		ENSG00000120725.12	SIL1	chr5_138888103_A_G_b38	0.0000026	0.22	Adrenal Gland
		ENSG00000120725.12	SIL1	chr5_138888103_A_G_b38	0.0000028	0.34	Brain - Nucleus accumbens (basal ganglia)
		ENSG00000044115.20	CTNNA1	chr5_138888103_A_G_b38	0.0000033	-0.14	Spleen
		ENSG00000120725.12	SIL1	chr5_138888103_A_G_b38	0.0000034	0.41	Brain - Substantia nigra
		ENSG00000044115.20	CTNNA1	chr5_138888103_A_G_b38	0.0000063	-0.082	Skin - Sun Exposed (Lower leg)
		ENSG00000044115.20	CTNNA1	chr5_138888103_A_G_b38	0.0000070	-0.13	Muscle - Skeletal
		ENSG00000120725.12	SIL1	chr5_138888103_A_G_b38	0.0000081	0.18	Colon - Transverse
		ENSG00000120725.12	SIL1	chr5_138888103_A_G_b38	0.000011	0.13	Heart - Left Ventricle
		ENSG00000044115.20	CTNNA1	chr5_138888103_A_G_b38	0.000026	-0.20	Brain - Cerebellar Hemisphere
		ENSG00000120725.12	SIL1	chr5_138888103_A_G_b38	0.000035	0.31	Brain - Anterior cingulate cortex (BA24)
		ENSG00000120725.12	SIL1	chr5_138888103_A_G_b38	0.000037	0.12	Breast - Mammary Tissue
		ENSG00000120725.12	SIL1	chr5_138888103_A_G_b38	0.000039	0.26	Artery - Coronary
		ENSG00000044115.20	CTNNA1	chr5_138888103_A_G_b38	0.000042	-0.12	Pancreas
		ENSG00000044115.20	CTNNA1	chr5_138888103_A_G_b38	0.000070	-0.10	Adipose - Subcutaneous
	rs288021	ENSG00000253404.1	AC034243.1	chr5_138888594_C_T_b38	2.9e-130	1.2	Thyroid
		ENSG00000253404.1	AC034243.1	chr5_138888594_C_T_b38	4.3e-92	1.1	Skin - Sun Exposed (Lower leg)
		ENSG00000253404.1	AC034243.1	chr5_138888594_C_T_b38	5.6e-85	1.0	Adipose - Subcutaneous
		ENSG00000253404.1	AC034243.1	chr5_138888594_C_T_b38	3.0e-74	1.1	Esophagus - Mucosa
		ENSG00000044115.20	CTNNA1	chr5_138888594_C_T_b38	9.9e-74	-0.49	Whole Blood
		ENSG00000253404.1	AC034243.1	chr5_138888594_C_T_b38	9.1e-70	1.0	Skin - Not Sun Exposed (Suprapubic)
		ENSG00000253404.1	AC034243.1	chr5_138888594_C_T_b38	9.7e-69	0.93	Nerve - Tibial
		ENSG00000253404.1	AC034243.1	chr5_138888594_C_T_b38	2.8e-65	1.2	Pituitary
		ENSG00000253404.1	AC034243.1	chr5_138888594_C_T_b38	8.3e-64	0.91	Lung
		ENSG00000253404.1	AC034243.1	chr5_138888594_C_T_b38	9.9e-63	1.0	Breast - Mammary Tissue
		ENSG00000253404.1	AC034243.1	chr5_138888594_C_T_b38	5.4e-57	0.92	Esophagus - Muscularis
		ENSG00000253404.1	AC034243.1	chr5_138888594_C_T_b38	2.3e-56	0.91	Adipose - Visceral (Omentum)
		ENSG00000253404.1	AC034243.1	chr5_138888594_C_T_b38	2.4e-55	1.2	Pancreas
		ENSG00000253404.1	AC034243.1	chr5_138888594_C_T_b38	9.5e-55	1.0	Heart - Atrial Appendage
		ENSG00000253404.1	AC034243.1	chr5_138888594_C_T_b38	1.4e-53	0.94	Stomach
		ENSG00000253404.1	AC034243.1	chr5_138888594_C_T_b38	1.3e-49	0.95	Heart - Left Ventricle
		ENSG00000253404.1	AC034243.1	chr5_138888594_C_T_b38	1.9e-47	1.1	Adrenal Gland
		ENSG00000253404.1	AC034243.1	chr5_138888594_C_T_b38	7.4e-46	0.77	Cells - Cultured fibroblasts
		ENSG00000253404.1	AC034243.1	chr5_138888594_C_T_b38	1.1e-41	0.89	Colon - Transverse
		ENSG00000044115.20	CTNNA1	chr5_138888594_C_T_b38	2.2e-40	-0.27	Esophagus - Mucosa
		ENSG00000253404.1	AC034243.1	chr5_138888594_C_T_b38	3.0e-37	0.91	Prostate
		ENSG00000253404.1	AC034243.1	chr5_138888594_C_T_b38	1.5e-34	0.89	Esophagus - Gastroesophageal Junction
		ENSG00000253404.1	AC034243.1	chr5_138888594_C_T_b38	3.1e-30	0.87	Colon - Sigmoid
		ENSG00000253404.1	AC034243.1	chr5_138888594_C_T_b38	1.0e-28	1.0	Brain - Putamen (basal ganglia)
		ENSG00000253404.1	AC034243.1	chr5_138888594_C_T_b38	1.2e-28	1.1	Brain - Caudate (basal ganglia)
		ENSG00000253404.1	AC034243.1	chr5_138888594_C_T_b38	2.6e-28	1.0	Spleen
		ENSG00000253404.1	AC034243.1	chr5_138888594_C_T_b38	6.1e-26	1.1	Ovary
		ENSG00000253404.1	AC034243.1	chr5_138888594_C_T_b38	9.0e-26	0.94	Liver
		ENSG00000253404.1	AC034243.1	chr5_138888594_C_T_b38	1.2e-25	1.0	Brain - Nucleus accumbens (basal ganglia)
		ENSG00000253404.1	AC034243.1	chr5_138888594_C_T_b38	5.6e-23	0.48	Testis
		ENSG00000253404.1	AC034243.1	chr5_138888594_C_T_b38	9.2e-23	0.87	Brain - Cortex
		ENSG00000253404.1	AC034243.1	chr5_138888594_C_T_b38	3.4e-21	1.1	Minor Salivary Gland
		ENSG00000253404.1	AC034243.1	chr5_138888594_C_T_b38	1.3e-20	0.85	Artery - Coronary
		ENSG00000253404.1	AC034243.1	chr5_138888594_C_T_b38	7.6e-20	1.0	Brain - Spinal cord (cervical c-1)
		ENSG00000253404.1	AC034243.1	chr5_138888594_C_T_b38	3.2e-19	0.94	Brain - Hippocampus
		ENSG00000253404.1	AC034243.1	chr5_138888594_C_T_b38	1.7e-18	0.99	Brain - Substantia nigra
		ENSG00000120725.12	SIL1	chr5_138888594_C_T_b38	2.7e-18	0.34	Esophagus - Muscularis
		ENSG00000253404.1	AC034243.1	chr5_138888594_C_T_b38	3.4e-18	0.90	Brain - Hypothalamus
		ENSG00000120725.12	SIL1	chr5_138888594_C_T_b38	6.3e-18	0.21	Nerve - Tibial
		ENSG00000253404.1	AC034243.1	chr5_138888594_C_T_b38	3.7e-17	1.1	Uterus
		ENSG00000253404.1	AC034243.1	chr5_138888594_C_T_b38	5.7e-17	1.1	Brain - Amygdala
		ENSG00000253404.1	AC034243.1	chr5_138888594_C_T_b38	5.8e-17	0.79	Brain - Frontal Cortex (BA9)
		ENSG00000120725.12	SIL1	chr5_138888594_C_T_b38	8.2e-17	0.22	Skin - Sun Exposed (Lower leg)
		ENSG00000253404.1	AC034243.1	chr5_138888594_C_T_b38	4.5e-16	1.1	Vagina
		ENSG00000253404.1	AC034243.1	chr5_138888594_C_T_b38	5.3e-16	0.79	Brain - Anterior cingulate cortex (BA24)
		ENSG00000253404.1	AC034243.1	chr5_138888594_C_T_b38	1.0e-15	0.78	Small Intestine - Terminal Ileum
		ENSG00000120725.12	SIL1	chr5_138888594_C_T_b38	1.3e-15	0.22	Skin - Not Sun Exposed (Suprapubic)
		ENSG00000120725.12	SIL1	chr5_138888594_C_T_b38	1.7e-15	0.29	Artery - Aorta
		ENSG00000120725.12	SIL1	chr5_138888594_C_T_b38	4.5e-14	0.29	Stomach
		ENSG00000253404.1	AC034243.1	chr5_138888594_C_T_b38	1.4e-12	1.1	Kidney - Cortex
		ENSG00000120725.12	SIL1	chr5_138888594_C_T_b38	1.5e-12	0.15	Thyroid
		ENSG00000120725.12	SIL1	chr5_138888594_C_T_b38	3.4e-10	0.16	Artery - Tibial
		ENSG00000120725.12	SIL1	chr5_138888594_C_T_b38	7.6e-10	0.16	Adipose - Subcutaneous
		ENSG00000120725.12	SIL1	chr5_138888594_C_T_b38	1.9e-9	0.30	Esophagus - Gastroesophageal Junction
		ENSG00000120725.12	SIL1	chr5_138888594_C_T_b38	3.6e-9	0.15	Adipose - Visceral (Omentum)
		ENSG00000120725.12	SIL1	chr5_138888594_C_T_b38	3.8e-9	0.14	Lung
		ENSG00000044115.20	CTNNA1	chr5_138888594_C_T_b38	4.0e-9	-0.11	Nerve - Tibial
		ENSG00000044115.20	CTNNA1	chr5_138888594_C_T_b38	5.2e-9	-0.13	Cells - Cultured fibroblasts
		ENSG00000120725.12	SIL1	chr5_138888594_C_T_b38	7.5e-9	0.43	Brain - Frontal Cortex (BA9)
		ENSG00000044115.20	CTNNA1	chr5_138888594_C_T_b38	2.1e-8	-0.15	Breast - Mammary Tissue

		ENSG00000120725.12	SIL1	chr5_138888594_C_T_b38	5.6e-8	0.12	Cells - Cultured fibroblasts
		ENSG00000120725.12	SIL1	chr5_138888594_C_T_b38	6.6e-8	0.26	Pituitary
		ENSG00000044115.20	CTNNA1	chr5_138888594_C_T_b38	1.9e-7	-0.15	Colon - Transverse
		ENSG00000044115.20	CTNNA1	chr5_138888594_C_T_b38	2.3e-7	0.10	Testis
		ENSG00000044115.20	CTNNA1	chr5_138888594_C_T_b38	2.6e-7	-0.097	Thyroid
		ENSG00000120725.12	SIL1	chr5_138888594_C_T_b38	3.5e-7	0.29	Brain - Cerebellum
		ENSG00000120725.12	SIL1	chr5_138888594_C_T_b38	3.9e-7	0.31	Brain - Cortex
		ENSG00000120725.12	SIL1	chr5_138888594_C_T_b38	4.2e-7	0.33	Brain - Caudate (basal ganglia)
		ENSG00000044115.20	CTNNA1	chr5_138888594_C_T_b38	4.8e-7	-0.097	Skin - Not Sun Exposed (Suprapubic)
		ENSG00000120725.12	SIL1	chr5_138888594_C_T_b38	7.5e-7	0.15	Esophagus - Mucosa
		ENSG00000044115.20	CTNNA1	chr5_138888594_C_T_b38	0.0000012	-0.13	Heart - Atrial Appendage
		ENSG00000044115.20	CTNNA1	chr5_138888594_C_T_b38	0.0000013	-0.21	Brain - Cerebellum
		ENSG00000120725.12	SIL1	chr5_138888594_C_T_b38	0.0000024	0.34	Brain - Nucleus accumbens (basal ganglia)
		ENSG00000120725.12	SIL1	chr5_138888594_C_T_b38	0.0000026	0.22	Adrenal Gland
		ENSG00000044115.20	CTNNA1	chr5_138888594_C_T_b38	0.0000030	-0.14	Spleen
		ENSG00000120725.12	SIL1	chr5_138888594_C_T_b38	0.0000034	0.41	Brain - Substantia nigra
		ENSG00000044115.20	CTNNA1	chr5_138888594_C_T_b38	0.0000047	-0.083	Skin - Sun Exposed (Lower leg)
		ENSG00000044115.20	CTNNA1	chr5_138888594_C_T_b38	0.0000054	-0.13	Muscle - Skeletal
		ENSG00000120725.12	SIL1	chr5_138888594_C_T_b38	0.0000081	0.18	Colon - Transverse
		ENSG00000120725.12	SIL1	chr5_138888594_C_T_b38	0.000012	0.13	Heart - Left Ventricle
		ENSG00000120725.12	SIL1	chr5_138888594_C_T_b38	0.000024	0.32	Brain - Anterior cingulate cortex (BA24)
		ENSG00000044115.20	CTNNA1	chr5_138888594_C_T_b38	0.000025	-0.20	Brain - Cerebellar Hemisphere
		ENSG00000120725.12	SIL1	chr5_138888594_C_T_b38	0.000039	0.26	Artery - Coronary
		ENSG00000044115.20	CTNNA1	chr5_138888594_C_T_b38	0.000042	-0.12	Pancreas
		ENSG00000120725.12	SIL1	chr5_138888594_C_T_b38	0.000047	0.12	Breast - Mammary Tissue
		ENSG00000044115.20	CTNNA1	chr5_138888594_C_T_b38	0.000050	-0.10	Adipose - Subcutaneous
		ENSG00000120725.12	SIL1	chr5_138888594_C_T_b38	0.000075	0.26	Brain - Putamen (basal ganglia)
	rs76723662	ENSG00000253404.1	AC034243.1	chr5_138891947_G_A_b38	1.3e-9	1.7	Skin - Not Sun Exposed (Suprapubic)
		ENSG00000253404.1	AC034243.1	chr5_138891947_G_A_b38	2.8e-8	1.6	Skin - Sun Exposed (Lower leg)
		ENSG00000253404.1	AC034243.1	chr5_138891947_G_A_b38	7.0e-8	2.0	Heart - Atrial Appendage
		ENSG00000253404.1	AC034243.1	chr5_138891947_G_A_b38	8.8e-7	1.3	Adipose - Subcutaneous
		ENSG00000253404.1	AC034243.1	chr5_138891947_G_A_b38	0.0000012	1.9	Heart - Left Ventricle
		ENSG00000253404.1	AC034243.1	chr5_138891947_G_A_b38	0.0000014	1.3	Lung
		ENSG00000253404.1	AC034243.1	chr5_138891947_G_A_b38	0.0000046	1.7	Esophagus - Gastroesophageal Junction
		ENSG00000253404.1	AC034243.1	chr5_138891947_G_A_b38	0.0000048	1.3	Thyroid
		ENSG00000253404.1	AC034243.1	chr5_138891947_G_A_b38	0.0000089	1.6	Colon - Sigmoid
		ENSG00000253404.1	AC034243.1	chr5_138891947_G_A_b38	0.000015	1.4	Colon - Transverse
		ENSG00000253404.1	AC034243.1	chr5_138891947_G_A_b38	0.000019	1.5	Breast - Mammary Tissue
		ENSG00000253404.1	AC034243.1	chr5_138891947_G_A_b38	0.000019	1.2	Adipose - Visceral (Omentum)
		ENSG00000253404.1	AC034243.1	chr5_138891947_G_A_b38	0.000028	1.5	Esophagus - Mucosa
		ENSG00000015479.18	MATR3	chr5_138891947_G_A_b38	0.000052	-1.1	Prostate
		ENSG00000253404.1	AC034243.1	chr5_138891947_G_A_b38	0.000056	1.1	Nerve - Tibial
	rs288009	ENSG00000253404.1	AC034243.1	chr5_138892973_G_A_b38	2.9e-130	1.2	Thyroid
		ENSG00000253404.1	AC034243.1	chr5_138892973_G_A_b38	4.3e-92	1.1	Skin - Sun Exposed (Lower leg)
		ENSG00000253404.1	AC034243.1	chr5_138892973_G_A_b38	5.6e-85	1.0	Adipose - Subcutaneous
		ENSG00000253404.1	AC034243.1	chr5_138892973_G_A_b38	3.0e-74	1.1	Esophagus - Mucosa
		ENSG00000044115.20	CTNNA1	chr5_138892973_G_A_b38	9.9e-74	-0.49	Whole Blood
		ENSG00000253404.1	AC034243.1	chr5_138892973_G_A_b38	9.1e-70	1.0	Skin - Not Sun Exposed (Suprapubic)
		ENSG00000253404.1	AC034243.1	chr5_138892973_G_A_b38	9.7e-69	0.93	Nerve - Tibial
		ENSG00000253404.1	AC034243.1	chr5_138892973_G_A_b38	2.8e-65	1.2	Pituitary
		ENSG00000253404.1	AC034243.1	chr5_138892973_G_A_b38	8.3e-64	0.91	Lung
		ENSG00000253404.1	AC034243.1	chr5_138892973_G_A_b38	9.9e-63	1.0	Breast - Mammary Tissue
		ENSG00000253404.1	AC034243.1	chr5_138892973_G_A_b38	5.4e-57	0.92	Esophagus - Muscularis
		ENSG00000253404.1	AC034243.1	chr5_138892973_G_A_b38	2.3e-56	0.91	Adipose - Visceral (Omentum)
		ENSG00000253404.1	AC034243.1	chr5_138892973_G_A_b38	2.4e-55	1.2	Pancreas
		ENSG00000253404.1	AC034243.1	chr5_138892973_G_A_b38	9.5e-55	1.0	Heart - Atrial Appendage
		ENSG00000253404.1	AC034243.1	chr5_138892973_G_A_b38	1.4e-53	0.94	Stomach
		ENSG00000253404.1	AC034243.1	chr5_138892973_G_A_b38	1.3e-49	0.95	Heart - Left Ventricle
		ENSG00000253404.1	AC034243.1	chr5_138892973_G_A_b38	1.9e-47	1.1	Adrenal Gland
		ENSG00000253404.1	AC034243.1	chr5_138892973_G_A_b38	7.4e-46	0.77	Cells - Cultured fibroblasts
		ENSG00000253404.1	AC034243.1	chr5_138892973_G_A_b38	1.1e-41	0.89	Colon - Transverse
		ENSG00000044115.20	CTNNA1	chr5_138892973_G_A_b38	2.2e-40	-0.27	Esophagus - Mucosa
		ENSG00000253404.1	AC034243.1	chr5_138892973_G_A_b38	3.0e-37	0.91	Prostate
		ENSG00000253404.1	AC034243.1	chr5_138892973_G_A_b38	1.5e-34	0.89	Esophagus - Gastroesophageal Junction
		ENSG00000253404.1	AC034243.1	chr5_138892973_G_A_b38	3.1e-30	0.87	Colon - Sigmoid
		ENSG00000253404.1	AC034243.1	chr5_138892973_G_A_b38	1.0e-28	1.0	Brain - Putamen (basal ganglia)
		ENSG00000253404.1	AC034243.1	chr5_138892973_G_A_b38	1.2e-28	1.1	Brain - Caudate (basal ganglia)
		ENSG00000253404.1	AC034243.1	chr5_138892973_G_A_b38	2.6e-28	1.0	Spleen
		ENSG00000253404.1	AC034243.1	chr5_138892973_G_A_b38	6.1e-26	1.1	Ovary
		ENSG00000253404.1	AC034243.1	chr5_138892973_G_A_b38	9.0e-26	0.94	Liver
		ENSG00000253404.1	AC034243.1	chr5_138892973_G_A_b38	1.2e-25	1.0	Brain - Nucleus accumbens (basal ganglia)
		ENSG00000253404.1	AC034243.1	chr5_138892973_G_A_b38	5.6e-23	0.48	Testis
		ENSG00000253404.1	AC034243.1	chr5_138892973_G_A_b38	9.2e-23	0.87	Brain - Cortex
		ENSG00000253404.1	AC034243.1	chr5_138892973_G_A_b38	3.4e-21	1.1	Minor Salivary Gland
		ENSG00000253404.1	AC034243.1	chr5_138892973_G_A_b38	1.3e-20	0.85	Artery - Coronary
		ENSG00000253404.1	AC034243.1	chr5_138892973_G_A_b38	7.6e-20	1.0	Brain - Spinal cord (cervical c-1)
		ENSG00000253404.1	AC034243.1	chr5_138892973_G_A_b38	3.2e-19	0.94	Brain - Hippocampus
		ENSG00000253404.1	AC034243.1	chr5_138892973_G_A_b38	1.7e-18	0.99	Brain - Substantia nigra
		ENSG00000120725.12	SIL1	chr5_138892973_G_A_b38	2.7e-18	0.34	Esophagus - Muscularis
		ENSG00000253404.1	AC034243.1	chr5_138892973_G_A_b38	3.4e-18	0.90	Brain - Hypothalamus
		ENSG00000120725.12	SIL1	chr5_138892973_G_A_b38	6.3e-18	0.21	Nerve - Tibial
		ENSG00000253404.1	AC034243.1	chr5_138892973_G_A_b38	3.7e-17	1.1	Uterus

		ENSG00000253404.1	AC034243.1	chr5_138892973_G_A_b38	5.7e-17	1.1	Brain - Amygdala
		ENSG00000253404.1	AC034243.1	chr5_138892973_G_A_b38	5.8e-17	0.79	Brain - Frontal Cortex (BA9)
		ENSG00000120725.12	SIL1	chr5_138892973_G_A_b38	8.2e-17	0.22	Skin - Sun Exposed (Lower leg)
		ENSG00000253404.1	AC034243.1	chr5_138892973_G_A_b38	4.5e-16	1.1	Vagina
		ENSG00000253404.1	AC034243.1	chr5_138892973_G_A_b38	5.3e-16	0.79	Brain - Anterior cingulate cortex (BA24)
		ENSG00000253404.1	AC034243.1	chr5_138892973_G_A_b38	1.0e-15	0.78	Small Intestine - Terminal Ileum
		ENSG00000120725.12	SIL1	chr5_138892973_G_A_b38	1.3e-15	0.22	Skin - Not Sun Exposed (Suprapubic)
		ENSG00000120725.12	SIL1	chr5_138892973_G_A_b38	1.7e-15	0.29	Artery - Aorta
		ENSG00000120725.12	SIL1	chr5_138892973_G_A_b38	4.5e-14	0.29	Stomach
		ENSG00000253404.1	AC034243.1	chr5_138892973_G_A_b38	1.4e-12	1.1	Kidney - Cortex
		ENSG00000120725.12	SIL1	chr5_138892973_G_A_b38	1.5e-12	0.15	Thyroid
		ENSG00000120725.12	SIL1	chr5_138892973_G_A_b38	3.4e-10	0.16	Artery - Tibial
		ENSG00000120725.12	SIL1	chr5_138892973_G_A_b38	7.6e-10	0.16	Adipose - Subcutaneous
		ENSG00000120725.12	SIL1	chr5_138892973_G_A_b38	1.9e-9	0.30	Esophagus - Gastroesophageal Junction
		ENSG00000120725.12	SIL1	chr5_138892973_G_A_b38	3.6e-9	0.15	Adipose - Visceral (Omentum)
		ENSG00000120725.12	SIL1	chr5_138892973_G_A_b38	3.8e-9	0.14	Lung
		ENSG00000044115.20	CTNNA1	chr5_138892973_G_A_b38	4.0e-9	-0.11	Nerve - Tibial
		ENSG00000044115.20	CTNNA1	chr5_138892973_G_A_b38	5.2e-9	-0.13	Cells - Cultured fibroblasts
		ENSG00000120725.12	SIL1	chr5_138892973_G_A_b38	7.5e-9	0.43	Brain - Frontal Cortex (BA9)
		ENSG00000044115.20	CTNNA1	chr5_138892973_G_A_b38	2.1e-8	-0.15	Breast - Mammary Tissue
		ENSG00000120725.12	SIL1	chr5_138892973_G_A_b38	5.6e-8	0.12	Cells - Cultured fibroblasts
		ENSG00000120725.12	SIL1	chr5_138892973_G_A_b38	6.6e-8	0.26	Pituitary
		ENSG00000044115.20	CTNNA1	chr5_138892973_G_A_b38	1.9e-7	-0.15	Colon - Transverse
		ENSG00000044115.20	CTNNA1	chr5_138892973_G_A_b38	2.3e-7	0.10	Testis
		ENSG00000044115.20	CTNNA1	chr5_138892973_G_A_b38	2.6e-7	-0.097	Thyroid
		ENSG00000120725.12	SIL1	chr5_138892973_G_A_b38	3.5e-7	0.29	Brain - Cerebellum
		ENSG00000120725.12	SIL1	chr5_138892973_G_A_b38	3.9e-7	0.31	Brain - Cortex
		ENSG00000120725.12	SIL1	chr5_138892973_G_A_b38	4.2e-7	0.33	Brain - Caudate (basal ganglia)
		ENSG00000044115.20	CTNNA1	chr5_138892973_G_A_b38	4.8e-7	-0.097	Skin - Not Sun Exposed (Suprapubic)
		ENSG00000120725.12	SIL1	chr5_138892973_G_A_b38	7.5e-7	0.15	Esophagus - Mucosa
		ENSG00000044115.20	CTNNA1	chr5_138892973_G_A_b38	0.0000012	-0.13	Heart - Atrial Appendage
		ENSG00000044115.20	CTNNA1	chr5_138892973_G_A_b38	0.0000013	-0.21	Brain - Cerebellum
		ENSG00000120725.12	SIL1	chr5_138892973_G_A_b38	0.0000024	0.34	Brain - Nucleus accumbens (basal ganglia)
		ENSG00000120725.12	SIL1	chr5_138892973_G_A_b38	0.0000026	0.22	Adrenal Gland
		ENSG00000044115.20	CTNNA1	chr5_138892973_G_A_b38	0.0000030	-0.14	Spleen
		ENSG00000120725.12	SIL1	chr5_138892973_G_A_b38	0.0000034	0.41	Brain - Substantia nigra
		ENSG00000044115.20	CTNNA1	chr5_138892973_G_A_b38	0.0000047	-0.083	Skin - Sun Exposed (Lower leg)
		ENSG00000044115.20	CTNNA1	chr5_138892973_G_A_b38	0.0000054	-0.13	Muscle - Skeletal
		ENSG00000120725.12	SIL1	chr5_138892973_G_A_b38	0.0000081	0.18	Colon - Transverse
		ENSG00000120725.12	SIL1	chr5_138892973_G_A_b38	0.000012	0.13	Heart - Left Ventricle
		ENSG00000120725.12	SIL1	chr5_138892973_G_A_b38	0.000024	0.32	Brain - Anterior cingulate cortex (BA24)
		ENSG00000044115.20	CTNNA1	chr5_138892973_G_A_b38	0.000025	-0.20	Brain - Cerebellar Hemisphere
		ENSG00000120725.12	SIL1	chr5_138892973_G_A_b38	0.000039	0.26	Artery - Coronary
		ENSG00000044115.20	CTNNA1	chr5_138892973_G_A_b38	0.000042	-0.12	Pancreas
		ENSG00000120725.12	SIL1	chr5_138892973_G_A_b38	0.000047	0.12	Breast - Mammary Tissue
		ENSG00000044115.20	CTNNA1	chr5_138892973_G_A_b38	0.000050	-0.10	Adipose - Subcutaneous
		ENSG00000120725.12	SIL1	chr5_138892973_G_A_b38	0.000075	0.26	Brain - Putamen (basal ganglia)
	rs111232820	ENSG00000253404.1	AC034243.1	chr5_138893515_A_G_b38	6.3e-9	1.6	Skin - Not Sun Exposed (Suprapubic)
		ENSG00000253404.1	AC034243.1	chr5_138893515_A_G_b38	2.0e-8	2.0	Heart - Atrial Appendage
		ENSG00000253404.1	AC034243.1	chr5_138893515_A_G_b38	3.8e-7	1.5	Skin - Sun Exposed (Lower leg)
		ENSG00000253404.1	AC034243.1	chr5_138893515_A_G_b38	8.1e-7	1.4	Adipose - Subcutaneous
		ENSG00000253404.1	AC034243.1	chr5_138893515_A_G_b38	0.0000014	1.3	Lung
		ENSG00000253404.1	AC034243.1	chr5_138893515_A_G_b38	0.0000080	1.9	Heart - Left Ventricle
		ENSG00000253404.1	AC034243.1	chr5_138893515_A_G_b38	0.000028	1.5	Esophagus - Mucosa
		ENSG00000253404.1	AC034243.1	chr5_138893515_A_G_b38	0.000030	1.4	Breast - Mammary Tissue
		ENSG00000253404.1	AC034243.1	chr5_138893515_A_G_b38	0.000047	1.5	Colon - Sigmoid
		ENSG00000253404.1	AC034243.1	chr5_138893515_A_G_b38	0.000048	1.5	Esophagus - Gastroesophageal Junction
		ENSG00000253404.1	AC034243.1	chr5_138893515_A_G_b38	0.000052	1.2	Thyroid
		ENSG00000015479.18	MATR3	chr5_138893515_A_G_b38	0.000052	-1.1	Prostate
		ENSG00000253404.1	AC034243.1	chr5_138893515_A_G_b38	0.000057	1.3	Colon - Transverse
		ENSG00000228672.3	PROB1	chr5_138893515_A_G_b38	0.000058	0.38	Muscle - Skeletal
	rs423469	ENSG00000253404.1	AC034243.1	chr5_138898427_G_C_b38	2.6e-145	1.2	Thyroid
		ENSG00000253404.1	AC034243.1	chr5_138898427_G_C_b38	2.4e-100	1.1	Skin - Sun Exposed (Lower leg)
		ENSG00000253404.1	AC034243.1	chr5_138898427_G_C_b38	1.9e-91	1.0	Adipose - Subcutaneous
		ENSG00000044115.20	CTNNA1	chr5_138898427_G_C_b38	1.2e-84	-0.51	Whole Blood
		ENSG00000253404.1	AC034243.1	chr5_138898427_G_C_b38	7.9e-83	1.1	Skin - Not Sun Exposed (Suprapubic)
		ENSG00000253404.1	AC034243.1	chr5_138898427_G_C_b38	5.4e-81	1.1	Esophagus - Mucosa
		ENSG00000253404.1	AC034243.1	chr5_138898427_G_C_b38	8.1e-78	0.95	Nerve - Tibial
		ENSG00000253404.1	AC034243.1	chr5_138898427_G_C_b38	7.8e-71	0.93	Lung
		ENSG00000253404.1	AC034243.1	chr5_138898427_G_C_b38	2.1e-66	1.0	Breast - Mammary Tissue
		ENSG00000253404.1	AC034243.1	chr5_138898427_G_C_b38	1.6e-65	0.94	Adipose - Visceral (Omentum)
		ENSG00000253404.1	AC034243.1	chr5_138898427_G_C_b38	1.7e-65	1.0	Heart - Atrial Appendage
		ENSG00000253404.1	AC034243.1	chr5_138898427_G_C_b38	3.6e-63	0.94	Esophagus - Muscularis
		ENSG00000253404.1	AC034243.1	chr5_138898427_G_C_b38	1.1e-62	1.1	Pancreas
		ENSG00000253404.1	AC034243.1	chr5_138898427_G_C_b38	3.9e-57	0.97	Heart - Left Ventricle
		ENSG00000253404.1	AC034243.1	chr5_138898427_G_C_b38	2.3e-55	0.92	Stomach
		ENSG00000253404.1	AC034243.1	chr5_138898427_G_C_b38	6.4e-55	1.2	Adrenal Gland
		ENSG00000253404.1	AC034243.1	chr5_138898427_G_C_b38	1.4e-54	1.2	Pituitary
		ENSG00000253404.1	AC034243.1	chr5_138898427_G_C_b38	9.3e-47	0.90	Colon - Transverse
		ENSG00000253404.1	AC034243.1	chr5_138898427_G_C_b38	3.7e-46	0.77	Cells - Cultured fibroblasts
		ENSG00000044115.20	CTNNA1	chr5_138898427_G_C_b38	3.6e-43	-0.27	Esophagus - Mucosa
		ENSG00000253404.1	AC034243.1	chr5_138898427_G_C_b38	5.1e-40	0.92	Esophagus - Gastroesophageal Junction

		ENSG00000253404.1	AC034243.1	chr5_138898427_G_C_b38	1.9e-38	0.90	Prostate
		ENSG00000253404.1	AC034243.1	chr5_138898427_G_C_b38	3.1e-34	0.89	Colon - Sigmoid
		ENSG00000253404.1	AC034243.1	chr5_138898427_G_C_b38	1.7e-29	0.97	Spleen
		ENSG00000253404.1	AC034243.1	chr5_138898427_G_C_b38	2.4e-29	0.96	Liver
		ENSG00000253404.1	AC034243.1	chr5_138898427_G_C_b38	6.4e-27	1.1	Ovary
		ENSG00000253404.1	AC034243.1	chr5_138898427_G_C_b38	2.1e-25	1.0	Brain - Nucleus accumbens (basal ganglia)
		ENSG00000253404.1	AC034243.1	chr5_138898427_G_C_b38	3.9e-25	1.1	Minor Salivary Gland
		ENSG00000253404.1	AC034243.1	chr5_138898427_G_C_b38	1.0e-24	1.0	Brain - Caudate (basal ganglia)
		ENSG00000253404.1	AC034243.1	chr5_138898427_G_C_b38	1.3e-24	0.98	Brain - Putamen (basal ganglia)
		ENSG00000253404.1	AC034243.1	chr5_138898427_G_C_b38	1.7e-23	0.86	Artery - Coronary
		ENSG00000253404.1	AC034243.1	chr5_138898427_G_C_b38	7.0e-23	0.47	Testis
		ENSG00000253404.1	AC034243.1	chr5_138898427_G_C_b38	6.0e-21	0.84	Brain - Cortex
		ENSG00000253404.1	AC034243.1	chr5_138898427_G_C_b38	1.4e-20	1.0	Brain - Substantia nigra
		ENSG00000253404.1	AC034243.1	chr5_138898427_G_C_b38	1.7e-20	0.98	Brain - Hypothalamus
		ENSG00000253404.1	AC034243.1	chr5_138898427_G_C_b38	6.2e-20	0.85	Small Intestine - Terminal Ileum
		ENSG00000253404.1	AC034243.1	chr5_138898427_G_C_b38	1.7e-19	0.85	Brain - Frontal Cortex (BA9)
		ENSG00000253404.1	AC034243.1	chr5_138898427_G_C_b38	2.3e-19	0.98	Brain - Hippocampus
		ENSG00000120725.12	SIL1	chr5_138898427_G_C_b38	2.7e-19	0.21	Nerve - Tibial
		ENSG00000120725.12	SIL1	chr5_138898427_G_C_b38	5.7e-19	0.34	Esophagus - Muscularis
		ENSG00000253404.1	AC034243.1	chr5_138898427_G_C_b38	5.7e-19	1.0	Brain - Amygdala
		ENSG00000253404.1	AC034243.1	chr5_138898427_G_C_b38	1.8e-18	0.99	Brain - Spinal cord (cervical c-1)
		ENSG00000253404.1	AC034243.1	chr5_138898427_G_C_b38	3.3e-18	1.0	Vagina
		ENSG00000120725.12	SIL1	chr5_138898427_G_C_b38	4.3e-16	0.22	Skin - Not Sun Exposed (Suprapubic)
		ENSG00000253404.1	AC034243.1	chr5_138898427_G_C_b38	6.6e-16	0.98	Uterus
		ENSG00000120725.12	SIL1	chr5_138898427_G_C_b38	1.0e-15	0.28	Artery - Aorta
		ENSG00000120725.12	SIL1	chr5_138898427_G_C_b38	1.4e-15	0.20	Skin - Sun Exposed (Lower leg)
		ENSG00000253404.1	AC034243.1	chr5_138898427_G_C_b38	1.7e-14	0.76	Brain - Anterior cingulate cortex (BA24)
		ENSG00000120725.12	SIL1	chr5_138898427_G_C_b38	2.9e-13	0.28	Stomach
		ENSG00000044115.20	CTNNA1	chr5_138898427_G_C_b38	4.7e-13	-0.16	Cells - Cultured fibroblasts
		ENSG00000253404.1	AC034243.1	chr5_138898427_G_C_b38	2.4e-11	1.0	Kidney - Cortex
		ENSG00000120725.12	SIL1	chr5_138898427_G_C_b38	1.6e-10	0.13	Thyroid
		ENSG00000120725.12	SIL1	chr5_138898427_G_C_b38	7.8e-10	0.30	Esophagus - Gastroesophageal Junction
		ENSG00000120725.12	SIL1	chr5_138898427_G_C_b38	1.2e-9	0.14	Cells - Cultured fibroblasts
		ENSG00000120725.12	SIL1	chr5_138898427_G_C_b38	2.4e-9	0.13	Lung
		ENSG00000120725.12	SIL1	chr5_138898427_G_C_b38	3.1e-9	0.15	Adipose - Subcutaneous
		ENSG00000044115.20	CTNNA1	chr5_138898427_G_C_b38	4.3e-9	-0.11	Nerve - Tibial
		ENSG00000044115.20	CTNNA1	chr5_138898427_G_C_b38	5.5e-9	-0.15	Breast - Mammary Tissue
		ENSG00000120725.12	SIL1	chr5_138898427_G_C_b38	1.4e-8	0.14	Adipose - Visceral (Omentum)
		ENSG00000120725.12	SIL1	chr5_138898427_G_C_b38	1.8e-8	0.43	Brain - Frontal Cortex (BA9)
		ENSG00000120725.12	SIL1	chr5_138898427_G_C_b38	2.3e-8	0.14	Artery - Tibial
		ENSG00000044115.20	CTNNA1	chr5_138898427_G_C_b38	4.9e-8	-0.10	Skin - Not Sun Exposed (Suprapubic)
		ENSG00000120725.12	SIL1	chr5_138898427_G_C_b38	1.1e-7	0.16	Esophagus - Mucosa
		ENSG00000120725.12	SIL1	chr5_138898427_G_C_b38	1.1e-7	0.32	Brain - Cortex
		ENSG00000120725.12	SIL1	chr5_138898427_G_C_b38	1.7e-7	0.34	Brain - Caudate (basal ganglia)
		ENSG00000044115.20	CTNNA1	chr5_138898427_G_C_b38	1.9e-7	-0.15	Muscle - Skeletal
		ENSG00000120725.12	SIL1	chr5_138898427_G_C_b38	2.1e-7	0.25	Pituitary
		ENSG00000120725.12	SIL1	chr5_138898427_G_C_b38	2.3e-7	0.29	Brain - Cerebellum
		ENSG00000120725.12	SIL1	chr5_138898427_G_C_b38	3.2e-7	0.36	Brain - Nucleus accumbens (basal ganglia)
		ENSG00000044115.20	CTNNA1	chr5_138898427_G_C_b38	0.0000012	-0.085	Skin - Sun Exposed (Lower leg)
		ENSG00000044115.20	CTNNA1	chr5_138898427_G_C_b38	0.0000012	-0.12	Heart - Atrial Appendage
		ENSG00000044115.20	CTNNA1	chr5_138898427_G_C_b38	0.0000013	-0.13	Colon - Transverse
		ENSG00000120725.12	SIL1	chr5_138898427_G_C_b38	0.0000013	0.42	Brain - Substantia nigra
		ENSG00000044115.20	CTNNA1	chr5_138898427_G_C_b38	0.0000014	0.095	Testis
		ENSG00000044115.20	CTNNA1	chr5_138898427_G_C_b38	0.0000016	-0.14	Spleen
		ENSG00000044115.20	CTNNA1	chr5_138898427_G_C_b38	0.0000017	-0.088	Thyroid
		ENSG00000120725.12	SIL1	chr5_138898427_G_C_b38	0.0000046	0.20	Adrenal Gland
		ENSG00000044115.20	CTNNA1	chr5_138898427_G_C_b38	0.0000052	-0.19	Brain - Cerebellum
		ENSG00000120725.12	SIL1	chr5_138898427_G_C_b38	0.0000078	0.13	Heart - Left Ventricle
		ENSG00000120725.12	SIL1	chr5_138898427_G_C_b38	0.000013	0.34	Brain - Spinal cord (cervical c-1)
		ENSG00000120725.12	SIL1	chr5_138898427_G_C_b38	0.000014	0.13	Breast - Mammary Tissue
		ENSG00000120725.12	SIL1	chr5_138898427_G_C_b38	0.000020	0.25	Artery - Coronary
		ENSG00000120725.12	SIL1	chr5_138898427_G_C_b38	0.000023	0.32	Brain - Anterior cingulate cortex (BA24)
		ENSG00000120725.12	SIL1	chr5_138898427_G_C_b38	0.000048	0.16	Colon - Transverse
		ENSG00000120725.12	SIL1	chr5_138898427_G_C_b38	0.000058	0.14	Pancreas
		ENSG00000120725.12	SIL1	chr5_138898427_G_C_b38	0.000061	0.26	Brain - Hippocampus
		ENSG00000044115.20	CTNNA1	chr5_138898427_G_C_b38	0.000071	-0.15	Colon - Sigmoid
		ENSG00000044115.20	CTNNA1	chr5_138898427_G_C_b38	0.000078	-0.096	Adipose - Subcutaneous
	rs10043722	ENSG00000253404.1	AC034243.1	chr5_138901252_A_G_b38	3.0e-85	0.92	Thyroid
		ENSG00000253404.1	AC034243.1	chr5_138901252_A_G_b38	7.1e-58	0.81	Skin - Sun Exposed (Lower leg)
		ENSG00000253404.1	AC034243.1	chr5_138901252_A_G_b38	1.2e-54	0.77	Adipose - Subcutaneous
		ENSG00000253404.1	AC034243.1	chr5_138901252_A_G_b38	4.0e-54	0.78	Nerve - Tibial
		ENSG00000253404.1	AC034243.1	chr5_138901252_A_G_b38	6.5e-49	0.81	Skin - Not Sun Exposed (Suprapubic)
		ENSG00000044115.20	CTNNA1	chr5_138901252_A_G_b38	1.5e-48	-0.38	Whole Blood
		ENSG00000253404.1	AC034243.1	chr5_138901252_A_G_b38	2.4e-46	0.85	Esophagus - Mucosa
		ENSG00000253404.1	AC034243.1	chr5_138901252_A_G_b38	2.5e-45	0.74	Lung
		ENSG00000253404.1	AC034243.1	chr5_138901252_A_G_b38	5.4e-43	0.84	Breast - Mammary Tissue
		ENSG00000253404.1	AC034243.1	chr5_138901252_A_G_b38	2.7e-41	0.86	Heart - Atrial Appendage
		ENSG00000253404.1	AC034243.1	chr5_138901252_A_G_b38	3.8e-37	0.73	Stomach
		ENSG00000253404.1	AC034243.1	chr5_138901252_A_G_b38	8.3e-37	0.86	Pancreas
		ENSG00000253404.1	AC034243.1	chr5_138901252_A_G_b38	1.1e-36	0.70	Esophagus - Muscularis
		ENSG00000253404.1	AC034243.1	chr5_138901252_A_G_b38	6.9e-35	0.70	Adipose - Visceral (Omentum)
		ENSG00000253404.1	AC034243.1	chr5_138901252_A_G_b38	5.1e-34	0.76	Heart - Left Ventricle

		ENSG00000253404.1	AC034243.1	chr5_138901252_A_G_b38	3.5e-28	0.58	Cells - Cultured fibroblasts
		ENSG00000044115.20	CTNNA1	chr5_138901252_A_G_b38	1.4e-27	-0.22	Esophagus - Mucosa
		ENSG00000253404.1	AC034243.1	chr5_138901252_A_G_b38	2.5e-26	0.67	Colon - Transverse
		ENSG00000253404.1	AC034243.1	chr5_138901252_A_G_b38	4.2e-26	0.71	Esophagus - Gastroesophageal Junction
		ENSG00000253404.1	AC034243.1	chr5_138901252_A_G_b38	1.0e-25	0.81	Adrenal Gland
		ENSG00000253404.1	AC034243.1	chr5_138901252_A_G_b38	4.1e-23	0.72	Prostate
		ENSG00000253404.1	AC034243.1	chr5_138901252_A_G_b38	7.0e-23	0.82	Pituitary
		ENSG00000253404.1	AC034243.1	chr5_138901252_A_G_b38	8.2e-22	0.69	Colon - Sigmoid
		ENSG00000253404.1	AC034243.1	chr5_138901252_A_G_b38	1.8e-21	0.79	Liver
		ENSG00000253404.1	AC034243.1	chr5_138901252_A_G_b38	6.7e-20	0.76	Artery - Coronary
		ENSG00000253404.1	AC034243.1	chr5_138901252_A_G_b38	9.0e-18	0.86	Ovary
		ENSG00000253404.1	AC034243.1	chr5_138901252_A_G_b38	7.4e-17	0.39	Testis
		ENSG00000253404.1	AC034243.1	chr5_138901252_A_G_b38	1.2e-16	0.71	Brain - Cortex
		ENSG00000253404.1	AC034243.1	chr5_138901252_A_G_b38	2.7e-16	0.71	Spleen
		ENSG00000253404.1	AC034243.1	chr5_138901252_A_G_b38	3.3e-16	0.88	Minor Salivary Gland
		ENSG00000253404.1	AC034243.1	chr5_138901252_A_G_b38	8.2e-16	0.75	Small Intestine - Terminal Ileum
		ENSG00000253404.1	AC034243.1	chr5_138901252_A_G_b38	1.0e-15	0.76	Brain - Nucleus accumbens (basal ganglia)
		ENSG00000120725.12	SIL1	chr5_138901252_A_G_b38	2.0e-15	0.28	Esophagus - Muscularis
		ENSG00000253404.1	AC034243.1	chr5_138901252_A_G_b38	7.9e-14	0.76	Brain - Caudate (basal ganglia)
		ENSG00000120725.12	SIL1	chr5_138901252_A_G_b38	9.6e-14	0.19	Skin - Not Sun Exposed (Suprapubic)
		ENSG00000253404.1	AC034243.1	chr5_138901252_A_G_b38	2.2e-12	0.65	Brain - Putamen (basal ganglia)
		ENSG00000120725.12	SIL1	chr5_138901252_A_G_b38	2.6e-12	0.16	Nerve - Tibial
		ENSG00000253404.1	AC034243.1	chr5_138901252_A_G_b38	5.2e-12	0.74	Brain - Spinal cord (cervical c-1)
		ENSG00000120725.12	SIL1	chr5_138901252_A_G_b38	6.8e-12	0.16	Skin - Sun Exposed (Lower leg)
		ENSG00000120725.12	SIL1	chr5_138901252_A_G_b38	4.6e-11	0.22	Artery - Aorta
		ENSG00000120725.12	SIL1	chr5_138901252_A_G_b38	1.2e-10	0.22	Stomach
		ENSG00000253404.1	AC034243.1	chr5_138901252_A_G_b38	1.5e-10	0.81	Brain - Amygdala
		ENSG00000253404.1	AC034243.1	chr5_138901252_A_G_b38	1.5e-10	0.62	Brain - Frontal Cortex (BA9)
		ENSG00000253404.1	AC034243.1	chr5_138901252_A_G_b38	2.1e-10	0.67	Brain - Hippocampus
		ENSG00000253404.1	AC034243.1	chr5_138901252_A_G_b38	2.2e-10	0.67	Brain - Hypothalamus
		ENSG00000253404.1	AC034243.1	chr5_138901252_A_G_b38	1.6e-9	0.72	Brain - Substantia nigra
		ENSG00000044115.20	CTNNA1	chr5_138901252_A_G_b38	3.6e-9	-0.12	Cells - Cultured fibroblasts
		ENSG00000120725.12	SIL1	chr5_138901252_A_G_b38	1.1e-8	0.11	Thyroid
		ENSG00000253404.1	AC034243.1	chr5_138901252_A_G_b38	1.8e-8	0.55	Brain - Anterior cingulate cortex (BA24)
		ENSG00000253404.1	AC034243.1	chr5_138901252_A_G_b38	3.0e-8	0.63	Vagina
		ENSG00000253404.1	AC034243.1	chr5_138901252_A_G_b38	3.8e-8	0.80	Kidney - Cortex
		ENSG00000120725.12	SIL1	chr5_138901252_A_G_b38	5.7e-8	0.12	Cells - Cultured fibroblasts
		ENSG00000253404.1	AC034243.1	chr5_138901252_A_G_b38	8.2e-8	0.66	Uterus
		ENSG00000044115.20	CTNNA1	chr5_138901252_A_G_b38	1.6e-7	-0.090	Skin - Not Sun Exposed (Suprapubic)
		ENSG00000044115.20	CTNNA1	chr5_138901252_A_G_b38	3.1e-7	-0.13	Colon - Transverse
		ENSG00000120725.12	SIL1	chr5_138901252_A_G_b38	8.0e-7	0.23	Esophagus - Gastroesophageal Junction
		ENSG00000120725.12	SIL1	chr5_138901252_A_G_b38	8.4e-7	0.12	Adipose - Subcutaneous
		ENSG00000120725.12	SIL1	chr5_138901252_A_G_b38	9.7e-7	0.15	Esophagus - Mucosa
		ENSG00000044115.20	CTNNA1	chr5_138901252_A_G_b38	0.0000026	-0.079	Thyroid
		ENSG00000120725.12	SIL1	chr5_138901252_A_G_b38	0.0000031	0.11	Artery - Tibial
		ENSG00000120725.12	SIL1	chr5_138901252_A_G_b38	0.0000044	0.34	Brain - Frontal Cortex (BA9)
		ENSG00000120725.12	SIL1	chr5_138901252_A_G_b38	0.0000045	0.099	Lung
		ENSG00000044115.20	CTNNA1	chr5_138901252_A_G_b38	0.0000047	-0.11	Breast - Mammary Tissue
		ENSG00000120725.12	SIL1	chr5_138901252_A_G_b38	0.0000048	0.16	Colon - Transverse
		ENSG00000120725.12	SIL1	chr5_138901252_A_G_b38	0.000011	0.11	Adipose - Visceral (Omentum)
		ENSG00000120725.12	SIL1	chr5_138901252_A_G_b38	0.000011	0.27	Brain - Caudate (basal ganglia)
		ENSG00000044115.20	CTNNA1	chr5_138901252_A_G_b38	0.000013	-0.075	Nerve - Tibial
		ENSG00000044115.20	CTNNA1	chr5_138901252_A_G_b38	0.000015	-0.069	Skin - Sun Exposed (Lower leg)
		ENSG00000120725.12	SIL1	chr5_138901252_A_G_b38	0.000019	0.23	Brain - Cerebellum
		ENSG00000170464.9	DNAJC18	chr5_138901252_A_G_b38	0.000048	-0.16	Muscle - Skeletal
		ENSG00000120725.12	SIL1	chr5_138901252_A_G_b38	0.000048	0.26	Brain - Nucleus accumbens (basal ganglia)
		ENSG00000044115.20	CTNNA1	chr5_138901252_A_G_b38	0.000063	-0.14	Colon - Sigmoid
		ENSG00000221947.7	XKR9	chr8_70335018_T_G_b38	0.0000091	-0.33	Esophagus - Muscularis
		ENSG00000221947.7	XKR9	chr8_70335018_T_G_b38	0.000019	-0.25	Skin - Sun Exposed (Lower leg)
	**rs35045592**	ENSG00000221947.7	XKR9	chr8_70335018_T_G_b38	0.000020	-0.29	Cells - Cultured fibroblasts
		ENSG00000221947.7	XKR9	chr8_70335018_T_G_b38	0.000042	-0.28	Skin - Not Sun Exposed (Suprapubic)
		ENSG00000221947.7	XKR9	chr8_70335018_T_G_b38	0.000054	-0.22	Muscle - Skeletal
		ENSG00000221947.7	XKR9	chr8_70335018_T_G_b38	0.000064	-0.38	Pancreas
	**rs1870649**	ENSG00000221947.7	XKR9	chr8_70348750_C_G_b38	0.000035	-0.30	Esophagus - Muscularis
**NCOA2**		ENSG00000221947.7	XKR9	chr8_70348750_C_G_b38	0.000055	-0.37	Pancreas
		ENSG00000262681.2	RP11-311F12.1	chr17_19649935_G_T_b38	7.4e-11	0.42	Brain - Caudate (basal ganglia)
		ENSG00000072210.18	ALDH3A2	chr17_19649935_G_T_b38	2.4e-10	0.17	Testis
		ENSG00000262681.2	RP11-311F12.1	chr17_19649935_G_T_b38	1.4e-8	0.38	Brain - Nucleus accumbens (basal ganglia)
		ENSG00000262681.2	RP11-311F12.1	chr17_19649935_G_T_b38	3.3e-8	0.42	Brain - Putamen (basal ganglia)
		ENSG00000142494.13	SLC47A1	chr17_19649935_G_T_b38	2.5e-7	0.22	Thyroid
		ENSG00000262681.2	RP11-311F12.1	chr17_19649935_G_T_b38	3.7e-7	0.19	Muscle - Skeletal
		ENSG00000072210.18	ALDH3A2	chr17_19649935_G_T_b38	0.0000010	0.13	Thyroid
		ENSG00000262681.2	RP11-311F12.1	chr17_19649935_G_T_b38	0.0000011	0.29	Nerve - Tibial
	**rs8069478**	ENSG00000262681.2	RP11-311F12.1	chr17_19649935_G_T_b38	0.0000029	0.23	Thyroid
		ENSG00000262681.2	RP11-311F12.1	chr17_19649935_G_T_b38	0.0000039	0.39	Brain - Hippocampus
		ENSG00000228983.9	SLC47A1P1	chr17_19649935_G_T_b38	0.0000056	-0.19	Adipose - Subcutaneous
		ENSG00000228983.9	SLC47A1P1	chr17_19649935_G_T_b38	0.0000062	-0.22	Nerve - Tibial
		ENSG00000262681.2	RP11-311F12.1	chr17_19649935_G_T_b38	0.0000070	0.37	Brain - Cortex
		ENSG00000262681.2	RP11-311F12.1	chr17_19649935_G_T_b38	0.0000079	0.42	Brain - Frontal Cortex (BA9)
		ENSG00000180638.17	SLC47A2	chr17_19649935_G_T_b38	0.000022	0.19	Thyroid
		ENSG00000072210.18	ALDH3A2	chr17_19649935_G_T_b38	0.000026	0.27	Brain - Cerebellum
**ALDH3A2**		ENSG00000262681.2	RP11-311F12.1	chr17_19649935_G_T_b38	0.000053	0.45	Brain - Cerebellar Hemisphere

	**rs4925036**	ENSG00000262681.2	RP11-311F12.1	chr17_19650346_A_G_b38	1.9e-11	0.44	Brain - Caudate (basal ganglia)
		ENSG00000072210.18	ALDH3A2	chr17_19650346_A_G_b38	1.4e-10	0.18	Testis
		ENSG00000262681.2	RP11-311F12.1	chr17_19650346_A_G_b38	5.2e-9	0.44	Brain - Putamen (basal ganglia)
		ENSG00000262681.2	RP11-311F12.1	chr17_19650346_A_G_b38	1.4e-8	0.38	Brain - Nucleus accumbens (basal ganglia)
		ENSG00000142494.13	SLC47A1	chr17_19650346_A_G_b38	2.3e-7	0.22	Thyroid
		ENSG00000072210.18	ALDH3A2	chr17_19650346_A_G_b38	3.9e-7	0.14	Thyroid
		ENSG00000262681.2	RP11-311F12.1	chr17_19650346_A_G_b38	4.1e-7	0.19	Muscle - Skeletal
		ENSG00000262681.2	RP11-311F12.1	chr17_19650346_A_G_b38	0.0000013	0.24	Thyroid
		ENSG00000262681.2	RP11-311F12.1	chr17_19650346_A_G_b38	0.0000015	0.28	Nerve - Tibial
		ENSG00000262681.2	RP11-311F12.1	chr17_19650346_A_G_b38	0.0000017	0.41	Brain - Hippocampus
		ENSG00000262681.2	RP11-311F12.1	chr17_19650346_A_G_b38	0.0000033	0.44	Brain - Frontal Cortex (BA9)
		ENSG00000262681.2	RP11-311F12.1	chr17_19650346_A_G_b38	0.0000037	0.38	Brain - Cortex
		ENSG00000228983.9	SLC47A1P1	chr17_19650346_A_G_b38	0.0000059	-0.22	Nerve - Tibial
		ENSG00000180638.17	SLC47A2	chr17_19650346_A_G_b38	0.0000085	0.20	Thyroid
		ENSG00000072210.18	ALDH3A2	chr17_19650346_A_G_b38	0.000015	0.28	Brain - Cerebellum
		ENSG00000228983.9	SLC47A1P1	chr17_19650346_A_G_b38	0.000016	-0.19	Adipose - Subcutaneous
		ENSG00000072210.18	ALDH3A2	chr17_19650346_A_G_b38	0.000038	0.14	Lung
		ENSG00000189423.11	USP32P3	chr17_19650346_A_G_b38	0.000050	-0.18	Nerve - Tibial
	**rs962800**	ENSG00000262681.2	RP11-311F12.1	chr17_19656155_A_G_b38	1.9e-11	-0.44	Brain - Caudate (basal ganglia)
		ENSG00000072210.18	ALDH3A2	chr17_19656155_A_G_b38	1.4e-10	-0.18	Testis
		ENSG00000262681.2	RP11-311F12.1	chr17_19656155_A_G_b38	5.2e-9	-0.44	Brain - Putamen (basal ganglia)
		ENSG00000262681.2	RP11-311F12.1	chr17_19656155_A_G_b38	1.4e-8	-0.38	Brain - Nucleus accumbens (basal ganglia)
		ENSG00000142494.13	SLC47A1	chr17_19656155_A_G_b38	2.3e-7	-0.22	Thyroid
		ENSG00000072210.18	ALDH3A2	chr17_19656155_A_G_b38	3.9e-7	-0.14	Thyroid
		ENSG00000262681.2	RP11-311F12.1	chr17_19656155_A_G_b38	4.1e-7	-0.19	Muscle - Skeletal
		ENSG00000262681.2	RP11-311F12.1	chr17_19656155_A_G_b38	0.0000013	-0.24	Thyroid
		ENSG00000262681.2	RP11-311F12.1	chr17_19656155_A_G_b38	0.0000015	-0.28	Nerve - Tibial
		ENSG00000262681.2	RP11-311F12.1	chr17_19656155_A_G_b38	0.0000017	-0.41	Brain - Hippocampus
		ENSG00000262681.2	RP11-311F12.1	chr17_19656155_A_G_b38	0.0000033	-0.44	Brain - Frontal Cortex (BA9)
		ENSG00000262681.2	RP11-311F12.1	chr17_19656155_A_G_b38	0.0000037	-0.38	Brain - Cortex
		ENSG00000228983.9	SLC47A1P1	chr17_19656155_A_G_b38	0.0000059	0.22	Nerve - Tibial
		ENSG00000180638.17	SLC47A2	chr17_19656155_A_G_b38	0.0000085	-0.20	Thyroid
		ENSG00000072210.18	ALDH3A2	chr17_19656155_A_G_b38	0.000015	-0.28	Brain - Cerebellum
		ENSG00000228983.9	SLC47A1P1	chr17_19656155_A_G_b38	0.000016	0.19	Adipose - Subcutaneous
		ENSG00000072210.18	ALDH3A2	chr17_19656155_A_G_b38	0.000038	-0.14	Lung
		ENSG00000189423.11	USP32P3	chr17_19656155_A_G_b38	0.000050	0.18	Nerve - Tibial
	**rs2386145**	ENSG00000262681.2	RP11-311F12.1	chr17_19659705_C_G_b38	1.9e-11	-0.44	Brain - Caudate (basal ganglia)
		ENSG00000072210.18	ALDH3A2	chr17_19659705_C_G_b38	1.4e-10	-0.18	Testis
		ENSG00000262681.2	RP11-311F12.1	chr17_19659705_C_G_b38	5.2e-9	-0.44	Brain - Putamen (basal ganglia)
		ENSG00000262681.2	RP11-311F12.1	chr17_19659705_C_G_b38	1.4e-8	-0.38	Brain - Nucleus accumbens (basal ganglia)
		ENSG00000142494.13	SLC47A1	chr17_19659705_C_G_b38	2.3e-7	-0.22	Thyroid
		ENSG00000072210.18	ALDH3A2	chr17_19659705_C_G_b38	3.9e-7	-0.14	Thyroid
		ENSG00000262681.2	RP11-311F12.1	chr17_19659705_C_G_b38	4.1e-7	-0.19	Muscle - Skeletal
		ENSG00000262681.2	RP11-311F12.1	chr17_19659705_C_G_b38	0.0000013	-0.24	Thyroid
		ENSG00000262681.2	RP11-311F12.1	chr17_19659705_C_G_b38	0.0000015	-0.28	Nerve - Tibial
		ENSG00000262681.2	RP11-311F12.1	chr17_19659705_C_G_b38	0.0000017	-0.41	Brain - Hippocampus
		ENSG00000262681.2	RP11-311F12.1	chr17_19659705_C_G_b38	0.0000033	-0.44	Brain - Frontal Cortex (BA9)
		ENSG00000262681.2	RP11-311F12.1	chr17_19659705_C_G_b38	0.0000037	-0.38	Brain - Cortex
		ENSG00000228983.9	SLC47A1P1	chr17_19659705_C_G_b38	0.0000059	0.22	Nerve - Tibial
		ENSG00000180638.17	SLC47A2	chr17_19659705_C_G_b38	0.0000085	-0.20	Thyroid
		ENSG00000072210.18	ALDH3A2	chr17_19659705_C_G_b38	0.000015	-0.28	Brain - Cerebellum
		ENSG00000228983.9	SLC47A1P1	chr17_19659705_C_G_b38	0.000016	0.19	Adipose - Subcutaneous
		ENSG00000072210.18	ALDH3A2	chr17_19659705_C_G_b38	0.000038	-0.14	Lung
		ENSG00000189423.11	USP32P3	chr17_19659705_C_G_b38	0.000050	0.18	Nerve - Tibial
	**rs2108971**	ENSG00000262681.2	RP11-311F12.1	chr17_19662632_G_A_b38	1.9e-11	-0.44	Brain - Caudate (basal ganglia)
		ENSG00000072210.18	ALDH3A2	chr17_19662632_G_A_b38	1.4e-10	-0.18	Testis
		ENSG00000262681.2	RP11-311F12.1	chr17_19662632_G_A_b38	5.2e-9	-0.44	Brain - Putamen (basal ganglia)
		ENSG00000262681.2	RP11-311F12.1	chr17_19662632_G_A_b38	1.4e-8	-0.38	Brain - Nucleus accumbens (basal ganglia)
		ENSG00000142494.13	SLC47A1	chr17_19662632_G_A_b38	2.3e-7	-0.22	Thyroid
		ENSG00000072210.18	ALDH3A2	chr17_19662632_G_A_b38	3.9e-7	-0.14	Thyroid
		ENSG00000262681.2	RP11-311F12.1	chr17_19662632_G_A_b38	4.1e-7	-0.19	Muscle - Skeletal
		ENSG00000262681.2	RP11-311F12.1	chr17_19662632_G_A_b38	0.0000013	-0.24	Thyroid
		ENSG00000262681.2	RP11-311F12.1	chr17_19662632_G_A_b38	0.0000015	-0.28	Nerve - Tibial
		ENSG00000262681.2	RP11-311F12.1	chr17_19662632_G_A_b38	0.0000017	-0.41	Brain - Hippocampus
		ENSG00000262681.2	RP11-311F12.1	chr17_19662632_G_A_b38	0.0000033	-0.44	Brain - Frontal Cortex (BA9)
		ENSG00000262681.2	RP11-311F12.1	chr17_19662632_G_A_b38	0.0000037	-0.38	Brain - Cortex
		ENSG00000228983.9	SLC47A1P1	chr17_19662632_G_A_b38	0.0000059	0.22	Nerve - Tibial
		ENSG00000180638.17	SLC47A2	chr17_19662632_G_A_b38	0.0000085	-0.20	Thyroid
		ENSG00000072210.18	ALDH3A2	chr17_19662632_G_A_b38	0.000015	-0.28	Brain - Cerebellum
		ENSG00000228983.9	SLC47A1P1	chr17_19662632_G_A_b38	0.000016	0.19	Adipose - Subcutaneous
		ENSG00000072210.18	ALDH3A2	chr17_19662632_G_A_b38	0.000038	-0.14	Lung
		ENSG00000189423.11	USP32P3	chr17_19662632_G_A_b38	0.000050	0.18	Nerve - Tibial
	**rs59755039**	ENSG00000262681.2	RP11-311F12.1	chr17_19665173_CT_C_b38	1.9e-11	-0.44	Brain - Caudate (basal ganglia)
		ENSG00000072210.18	ALDH3A2	chr17_19665173_CT_C_b38	1.4e-10	-0.18	Testis
		ENSG00000262681.2	RP11-311F12.1	chr17_19665173_CT_C_b38	5.2e-9	-0.44	Brain - Putamen (basal ganglia)
		ENSG00000262681.2	RP11-311F12.1	chr17_19665173_CT_C_b38	1.4e-8	-0.38	Brain - Nucleus accumbens (basal ganglia)
		ENSG00000142494.13	SLC47A1	chr17_19665173_CT_C_b38	2.3e-7	-0.22	Thyroid
		ENSG00000072210.18	ALDH3A2	chr17_19665173_CT_C_b38	3.9e-7	-0.14	Thyroid
		ENSG00000262681.2	RP11-311F12.1	chr17_19665173_CT_C_b38	4.1e-7	-0.19	Muscle - Skeletal
		ENSG00000262681.2	RP11-311F12.1	chr17_19665173_CT_C_b38	0.0000013	-0.24	Thyroid
		ENSG00000262681.2	RP11-311F12.1	chr17_19665173_CT_C_b38	0.0000015	-0.28	Nerve - Tibial
		ENSG00000262681.2	RP11-311F12.1	chr17_19665173_CT_C_b38	0.0000017	-0.41	Brain - Hippocampus

		ENSG00000262681.2	RP11-311F12.1	chr17_19665173_CT_C_b38	0.0000033	-0.44	Brain - Frontal Cortex (BA9)
		ENSG00000262681.2	RP11-311F12.1	chr17_19665173_CT_C_b38	0.0000037	-0.38	Brain - Cortex
		ENSG00000228983.9	SLC47A1P1	chr17_19665173_CT_C_b38	0.0000059	0.22	Nerve - Tibial
		ENSG00000180638.17	SLC47A2	chr17_19665173_CT_C_b38	0.0000085	-0.20	Thyroid
		ENSG00000072210.18	ALDH3A2	chr17_19665173_CT_C_b38	0.000015	-0.28	Brain - Cerebellum
		ENSG00000228983.9	SLC47A1P1	chr17_19665173_CT_C_b38	0.000016	0.19	Adipose - Subcutaneous
		ENSG00000072210.18	ALDH3A2	chr17_19665173_CT_C_b38	0.000038	-0.14	Lung
		ENSG00000189423.11	USP32P3	chr17_19665173_CT_C_b38	0.000050	0.18	Nerve - Tibial
	**rs8069576**	ENSG00000262681.2	RP11-311F12.1	chr17_19667007_A_G_b38	1.9e-11	-0.44	Brain - Caudate (basal ganglia)
		ENSG00000072210.18	ALDH3A2	chr17_19667007_A_G_b38	1.4e-10	-0.18	Testis
		ENSG00000262681.2	RP11-311F12.1	chr17_19667007_A_G_b38	5.2e-9	-0.44	Brain - Putamen (basal ganglia)
		ENSG00000262681.2	RP11-311F12.1	chr17_19667007_A_G_b38	1.4e-8	-0.38	Brain - Nucleus accumbens (basal ganglia)
		ENSG00000142494.13	SLC47A1	chr17_19667007_A_G_b38	2.3e-7	-0.22	Thyroid
		ENSG00000072210.18	ALDH3A2	chr17_19667007_A_G_b38	3.9e-7	-0.14	Thyroid
		ENSG00000262681.2	RP11-311F12.1	chr17_19667007_A_G_b38	4.1e-7	-0.19	Muscle - Skeletal
		ENSG00000262681.2	RP11-311F12.1	chr17_19667007_A_G_b38	0.0000013	-0.24	Thyroid
		ENSG00000262681.2	RP11-311F12.1	chr17_19667007_A_G_b38	0.0000015	-0.28	Nerve - Tibial
		ENSG00000262681.2	RP11-311F12.1	chr17_19667007_A_G_b38	0.0000017	-0.41	Brain - Hippocampus
		ENSG00000262681.2	RP11-311F12.1	chr17_19667007_A_G_b38	0.0000033	-0.44	Brain - Frontal Cortex (BA9)
		ENSG00000262681.2	RP11-311F12.1	chr17_19667007_A_G_b38	0.0000037	-0.38	Brain - Cortex
		ENSG00000228983.9	SLC47A1P1	chr17_19667007_A_G_b38	0.0000059	0.22	Nerve - Tibial
		ENSG00000180638.17	SLC47A2	chr17_19667007_A_G_b38	0.0000085	-0.20	Thyroid
		ENSG00000072210.18	ALDH3A2	chr17_19667007_A_G_b38	0.000015	-0.28	Brain - Cerebellum
		ENSG00000228983.9	SLC47A1P1	chr17_19667007_A_G_b38	0.000016	0.19	Adipose - Subcutaneous
		ENSG00000072210.18	ALDH3A2	chr17_19667007_A_G_b38	0.000038	-0.14	Lung
		ENSG00000189423.11	USP32P3	chr17_19667007_A_G_b38	0.000050	0.18	Nerve - Tibial
